# Nanoscale covalent organic frameworks as theranostic platforms for oncotherapy: synthesis, functionalization, and applications

**DOI:** 10.1039/d0na00537a

**Published:** 2020-07-16

**Authors:** Qun Guan, Guang-Bo Wang, Le-Le Zhou, Wen-Yan Li, Yu-Bin Dong

**Affiliations:** College of Chemistry, Chemical Engineering and Materials Science, Collaborative Innovation Center of Functionalized Probes for Chemical Imaging in Universities of Shandong, Key Laboratory of Molecular and Nano Probes, Ministry of Education, Shandong Normal University Jinan 250014 P. R. China guangbo.wang@sdnu.edu.cn yubindong@sdnu.edu.cn

## Abstract

Cancer nanomedicine is one of the most promising domains that has emerged in the continuing search for cancer diagnosis and treatment. The rapid development of nanomaterials and nanotechnology provide a vast array of materials for use in cancer nanomedicine. Among the various nanomaterials, covalent organic frameworks (COFs) are becoming an attractive class of upstarts owing to their high crystallinity, structural regularity, inherent porosity, extensive functionality, design flexibility, and good biocompatibility. In this comprehensive review, recent developments and key achievements of COFs are provided, including their structural design, synthesis methods, nanocrystallization, and functionalization strategies. Subsequently, a systematic overview of the potential oncotherapy applications achieved till date in the fast-growing field of COFs is provided with the aim to inspire further contributions and developments to this nascent but promising field. Finally, development opportunities, critical challenges, and some personal perspectives for COF-based cancer therapeutics are presented.

## Abstract 2

We outline the latest developments in COF-based nanomedicines for use in oncotherapy, including material synthesis, nanocrystallization, and functionalization strategies, as well as their therapeutics applications.

## Graphical abstract

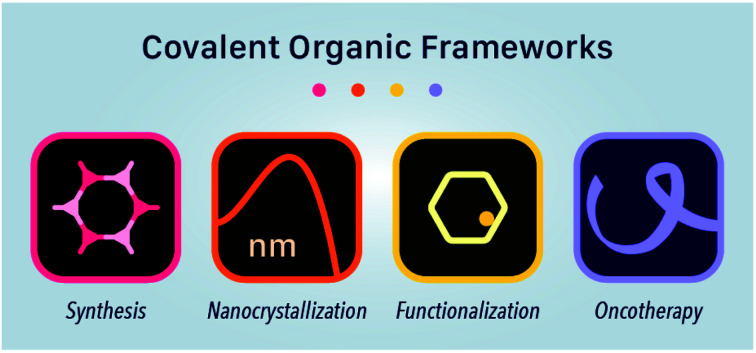

## 1 Introduction

Cancer remains a worldwide public health issue with high morbidity and mortality rates.^1^ It is estimated that by 2030, the number of cancer cases will increase to 24.6 million, while the number of cancer deaths can reach around 13 million.^2^ In recent years, increasing number of researchers in the fields of chemistry, materials science, biology, and medicine have turned their research interest towards rational designing and preparation of nanopharmaceuticals for tumor diagnosis and treatment^3–6^ due to drawbacks in conventional therapies, such as chemotherapy, radiotherapy, and surgical resection.^7^ Nanoparticle-based drug delivery, which integrates emerging nanotechnologies with traditional chemotherapeutic drugs to get rid of drawbacks in traditional therapies as well as offer new possibilities to optimize cancer treatment, has always been one of the focuses in the field of nanomedicine.^8–10^ In general, the key advantages of nanodrug delivery are longer circulating half-lives, improved pharmacokinetics, selective intratumoral accumulation, and lower systemic toxicity. Meanwhile, some other emerging minimally invasive therapies, such as photothermal therapy (PTT) and photodynamic therapy (PDT), have also exhibited promising potential in oncotherapy due to their high selectivity, low side-effects, and negligible drug resistance.^11–14^ Rapid developments in nanomaterials and nanotechnology have provided a vast material reservoir for use in cancer nanomedicine, which mainly include mesoporous silica,^15^ metal chalcogenides,^16^ upconversion materials,^17^ MXenes,^18,19^ carbon-based materials,^20,21^ semiconducting polymers,^22,23^ and liposomes.^24^

The design, synthesis, and applications of advanced porous materials with specific structures at the micron- and nanoscales have been a research hotspot in various scientific fields;^25–30^ further, the development of porous materials ranging from traditional inorganic materials (such as zeolites, silicas, and activated carbons) to organic–inorganic hybrid porous materials (such as metal–organic cages (MOCs),^31^ coordination polymers (CPs),^32^ and metal–organic frameworks (MOFs)).^33–35^ Among them, MOFs are crystalline materials formed by the self-assembly of organic ligands and metal ions (or clusters) through coordination bonds. The highly ordered structures of MOFs allow precise control over their pore shapes and chemical environments, thereby realizing controllable regulation of their properties.^36^ In the past decade, MOFs have been widely applied in the field of oncology and have even entered the stage of clinical trials.^37–39^ With the development of reticular chemistry,^40^ a new generation of crystalline porous materials, namely, covalent organic frameworks (COFs), emerged in 2005^41^ and have been booming in recent years.^42^ As a natural extension of MOFs, COFs are composed of nonmetallic elements (*e.g.*, C, H, N, O, and B) connected by strong covalent bonds into two-dimensional (2D) or three-dimensional (3D) crystalline frameworks with predictable and periodic structures.^43,44^ Due to the diversity of organic syntheses, COFs provide promising prospects for materials design, enabling function- and application-oriented material syntheses. Until now, COFs have been widely used for separation and analysis,^45–49^ heterogeneous catalysis,^50–52^ sensing,^53^ optoelectronics,^54^ energy and environmental science,^55–59^ and biomedicine.^60,61^

In recent years, COFs, particularly nanoscale COFs (NCOFs), have joined a huge candidate library of biomedical nanomaterials because of their following unique features. (i) On account of their modular structures, COFs can be easily decorated with multiple functional compositions, enabling diverse biomedical applications, such as tumor targeting, fluorescence imaging, and cancer therapy. (ii) Due to their inherent porosity, COF cavities allow the encapsulation of various guest molecules, thereby facilitating controlled drug release. (iii) Owing to their conjugated structures, the energy level structure of a COF monomer is different in the framework. By tuning the topological structures and geometric parameters to optimize the directional energy and charge transport, COFs may have optical properties that cannot be realized within the monomers, which offers additional and unexpected possibilities for imaging and therapeutic applications of COFs. (iv) The metal-free nature of COFs prevents any potential biological toxicity caused by metal elements.^62^ To sum up, we believe that COFs are becoming a promising and efficient organic material platform for building theranostic systems.

**Fig. 1:**
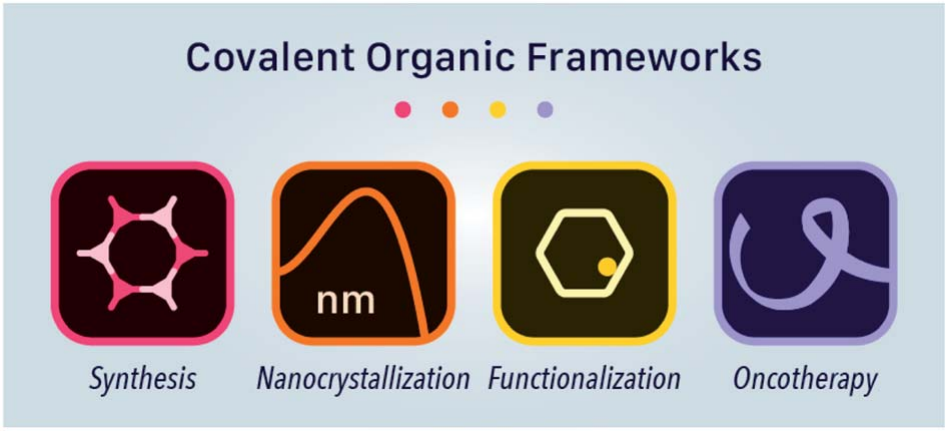
Synthesis, nanocrystallization, functionalization, and oncotherapy applications of COFs.

In this review (Fig. 1), we systematically summarized the rational design and preparation strategies of COFs, focusing on their nanocrystallization and functionalization strategies, with emphasis on their specific applications in tumor nanotherapeutics. Finally, the remaining challenges and possible future trends of COFs for tumor nanotherapeutics were discussed, expecting to promote further development of COFs for oncotherapy.

## 2 Structures and characterizations of COFs

### 2.1 Structures of COFs

**Fig. 2:**
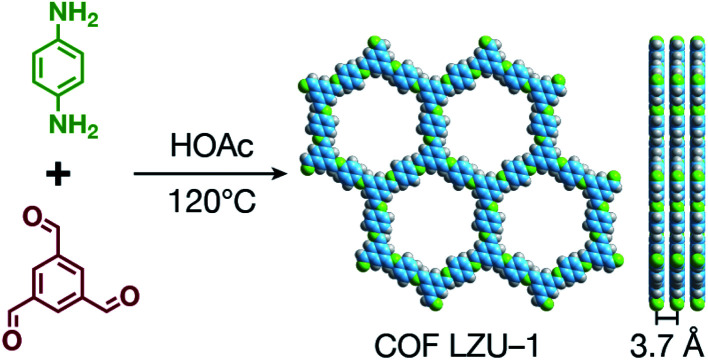
Typical structure of 2D COF LZU-1.

COFs are generally defined as crystalline, extended 2D and 3D networks with permanent pores constructed by different organic building blocks connected *via* covalent bonds.^63^ Until now, most of the reported COFs have been 2D structures. The structure of a 2D COF consists of 2D sheets held together by covalent bonds, which are then stacked together through noncovalent π–π interactions. For example, 2D monosheets of COF LZU-1 use the face-to-face eclipsed stacking (Fig. 2),^64^ which is also known as AA stacking: this is the most common stacking type for 2D COFs. Besides AA stacking, other stacking types, such as staggered AB,^65–67^ ABC,^68^ and ABCD^69^ stacking, can also be formed during the assembly of 2D COFs.

**Fig. 3:**
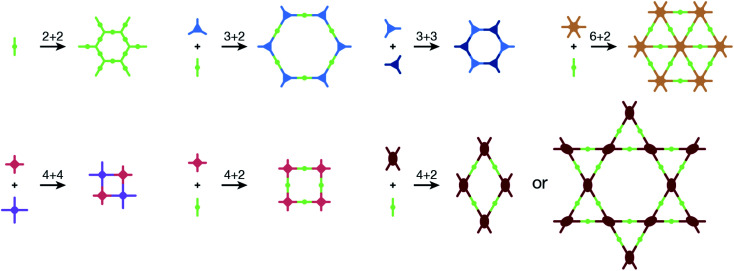
Common monomer geometries and topological diagrams for the synthesis of 2D COFs.

**Fig. 4:**
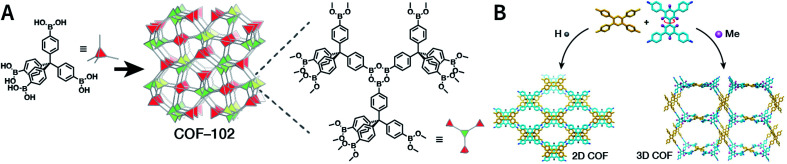
Topological structures of 3D COFs. (A) COF-102 composed of a tetrahedral monomer.^73^ (B) Synthesis of 3D COFs by twisting a monomer from planar to tetrahedral symmetry with steric hindrance. Adapted with permission.^74^ Copyright 2020, American Chemical Society.

COFs are modular in nature. The reactive functional groups (including species and number) and molecular geometry (*e.g.*, length, directionality, and symmetry) of the monomers enable to predefine the geometry and topology of the resultant frameworks. Therefore, unlike amorphous polymers, COFs provide positional control over their monomers in the spatial dimension,^70^ thereby realizing the possibility of the oriented design of frameworks and pore structures. For example, in the 2D plane, trigonal planar monomers can co-condense to form sheets with hexagonal pores, while tetragonal monomers can co-condense with linear monomers to form tetragonal, rhombic, or Kagome pores (Fig. 3). An interesting subject in mathematics, namely, plane tessellation, which refers to completely covering a plane using one or more geometric shapes without overlaps and gaps, may be a useful guide for the topological structure design of COFs, particularly 2D COFs with hierarchical porosity.^71,72^ However, in terms of topology, 3D topology^44^ is expected to be more colorful and complex than 2D topology. As shown in Fig. 4, using polyhedral instead of polygonal monomers^73^ or adding geometric constraints to the 2D monomers^74,75^ can possibly afford 3D COFs. In particular, the combination of tetrahedral monomers and triangular linkages results in the formation of **ctn** or **bor** topology, whereas the combination of tetrahedral and linear monomers usually leads to the **dia** topology.^73^

**Fig. 5:**
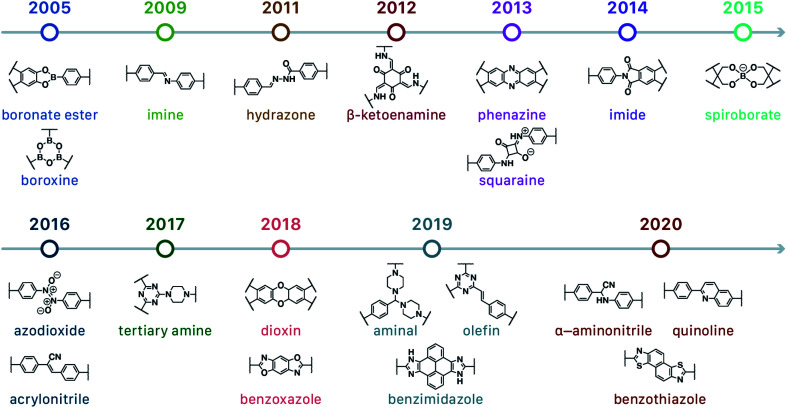
Timeline of various linkages for COF formation.

**Fig. 6:**
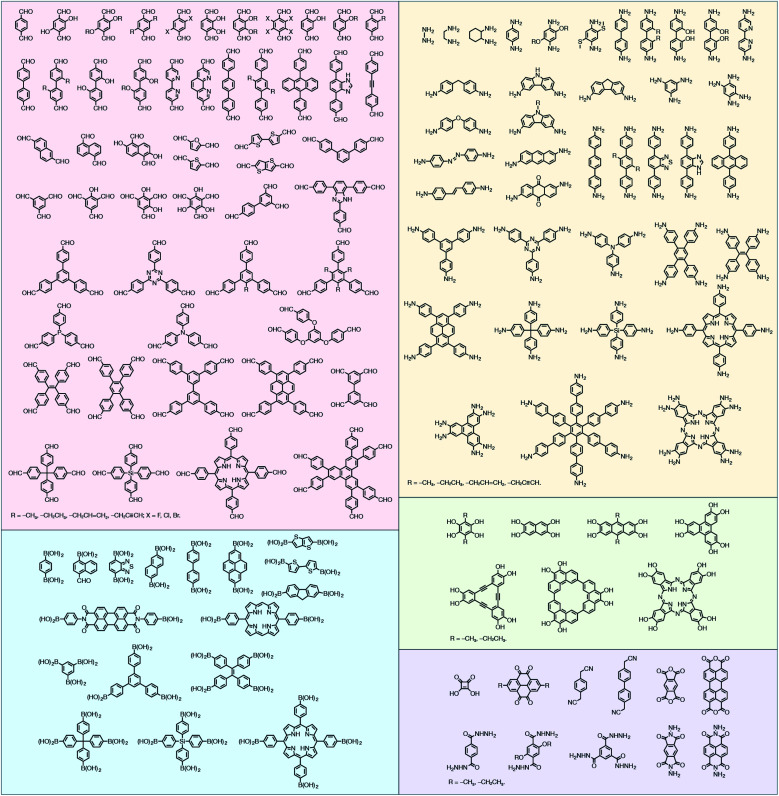
Certain commercially available monomers that have been used in the construction of COFs.

In contrast to MOFs based on coordination bonds, a considerable amount of research in the field of COFs has been devoted toward the development of new chemical bonds that constitute linkages.^76^ For each new linkage, finding appropriate crystallization conditions is the first challenge. In order to fabricate extended crystalline solids, covalent bonds formed between the monomers are usually reversible under the given reaction conditions, and the reaction rate must be sufficiently fast to allow sufficient defect self-correction.^77,78^ In recent years, conventionally considered irreversible chemical bonds have also been successfully used to construct COFs,^79,80^ and these new examples have given a strong impetus toward the theoretical research of COFs. Common linkages reported so far and their corresponding monomers are shown in Fig. 5 and 6.

### 2.2 Characterization of COFs

Generally, the first step for the characterization of COFs is determining their crystal structures. Typically, the structure of a crystalline material is determined by the single-crystal X-ray diffraction (SC-XRD) technique. However, almost all the reported COFs are microcrystalline aggregates in their powder form; it is particularly challenging to obtain high-quality, large-sized single crystals that meet the requirements of SC-XRD measurements.^81^ In this context, powder diffraction crystallography has become the most powerful technique for the determination of COF structures. By combining experimental and simulation results with structural refinements, the structure of a COF can be optimized and perfectly determined. This simulation–experiment–refinement trilogy has become almost a standard procedure for structural designation *via* COF diffraction crystallography.^82^ Among them, X-ray diffraction is the most common diffraction technique, such as powder X-ray diffraction (PXRD) and small-/wide-angle X-ray scattering (SAXS/WAXS).^83^ For 3D COFs, apart from the aforementioned techniques, electron diffraction also plays an important role for interpenetrated structural analyses.^84,85^

Certain spectroscopy methods can also be used for auxiliary research on the chemical structures of COFs. For example, Fourier-transform infrared spectroscopy (FT-IR) is widely used for linkage identification, ^13^C cross-polarization magic-angle spinning solid-state nuclear magnetic resonance spectroscopy (^13^C CP-MAS ssNMR)^86^ is used to designate the chemical environments of carbon atoms, and X-ray photoelectron spectroscopy (XPS) can reveal the chemical structures of COF surfaces.^87^

Another important feature of COFs, namely, their permanent pore structures, can be evaluated by gas adsorption and desorption experiments, which can provide valuable information regarding the specific surface area, pore size, and pore volume of COFs. Currently, the most common test gas is nitrogen at 77 K and the optimal analysis approach to obtain the specific surface area is the Brunauer–Emmett–Teller (BET) theory based on a multilayer gas adsorption model.^88^ On the other hand, the pore volume and pore size distribution of COFs can be determined by various approaches,^89^ such as nonlocal density functional theory (NLDFT), quenched solid density functional theory (QSDFT), grand canonical Monte Carlo (GCMC) method, Barrett–Joyner–Halenda (BJH) method, and Horvath–Kawazoe (HK) method. However, the analysis methods should be carefully selected according to the characteristics of different COF materials; otherwise, it can lead to inaccurate or completely incorrect analysis results.^90^ Since the expected information regarding the pore structure can also be calculated from the crystal structure, it is very meaningful to compare the experimental results with the theoretical predictions.

Morphology, including particle shape and size, is significant for COF characterization. It is a common practice to observe the microscopic morphology of particles with electron microscopes, such as scanning electron microscopy (SEM), transmission electron microscopy (TEM), high-resolution transmission electron microscopy (HRTEM), atomic force microscopy (AFM), and high-angle annular dark-field scanning transmission electron microscopy (HAADF-STEM). Lattice spacing and diffraction pattern obtained by HRTEM can provide additional assistance for the structural analyses of COFs. By combining with energy-dispersive X-ray spectroscopy (EDX), elemental distribution can also be semiquantitatively determined. In addition, dynamic light scattering (DLS) measurements can provide statistical distribution of the hydrodynamic diameters of the particles, and it plays an indispensable role in the study of uniformity and stability of NCOFs.^40,91^

## 3 Synthesis of COFs

**Fig. 7:**
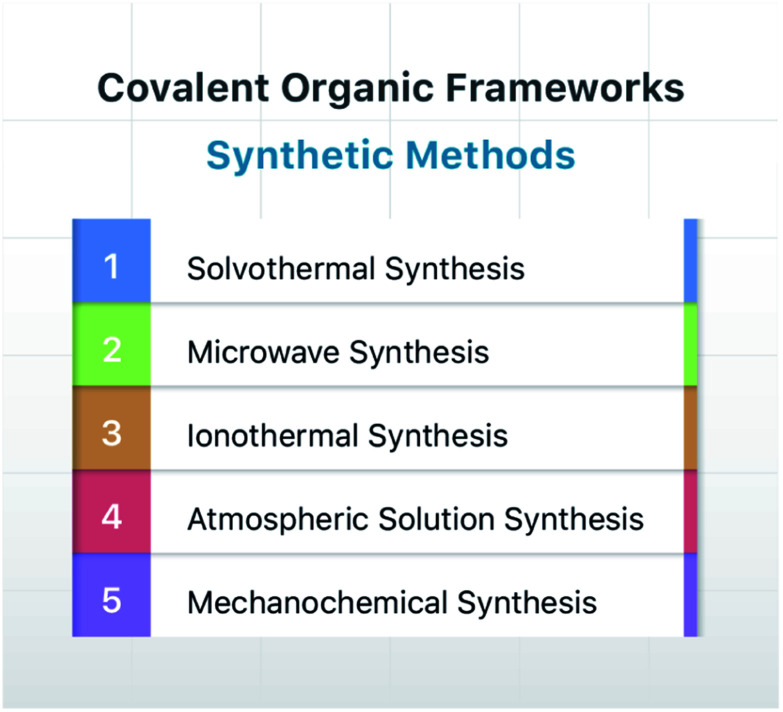
Typical synthesis methods of COFs.

Since the group of Yaghi pioneered the preparation of the first COF material under solvothermal conditions in 2005,^41^ various synthesis methods have been employed and reported for the synthesis of COFs to satisfy the needs of extensive applications. By using relevant examples, this section will summarize and discuss the conventional synthesis methods of COFs, including solvothermal synthesis, microwave synthesis, ionothermal synthesis, atmospheric solution synthesis, and mechanochemical synthesis (Fig. 7).

### 3.1 Solvothermal synthesis

Solvothermal synthesis refers to a method for preparing advanced materials in a sealed pressure container at a certain temperature and solvent autogenous pressure through the process of dissolution and recrystallization of raw materials.^92^ So far, most of the reported COFs have been synthesized under solvothermal conditions, including the earliest reported ones, *i.e.*, COF-1 and COF-5.^41^ When the solvent is water, it is referred to as hydrothermal synthesis. The hydrothermal synthesis of COFs is exceedingly rare,^93^ while the preparation of COFs in a mixed solution of organic solvent and water has been realized.^94,95^

**Fig. 8:**
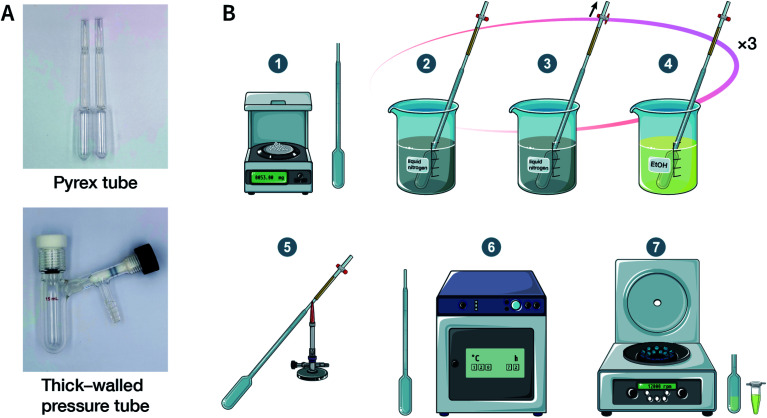
Solvothermal synthesis of COFs. (A) Digital photographs of a Pyrex tube and a thick-walled pressure tube for COF solvothermal synthesis. (B) Conventional steps in COF solvothermal synthesis. (1) Mixing the ingredients; (2) freezing with liquid nitrogen; (3) pump down; (4) thawing; (5) flame sealing; (6) oven heating; (7) separation of solids.

For solvothermal synthesis, stainless steel reaction kettles with polytetrafluoroethylene (PTFE) lining are the most general pressure vessels. Nevertheless, it is difficult to isolate the air, making it unsuitable for the synthesis of COFs. Therefore, typical COF solvothermal synthesis is usually carried out in a Pyrex tube; a thick-walled pressure tube can also be used instead of a disposable Pyrex tube (Fig. 8A). The general synthesis steps are shown in Fig. 8B. In brief, the calculated amount of monomers and solvents are added to the Pyrex tube; after several freeze–pump–thaw cycles, the Pyrex tube is sealed to preserve the produced water molecules to maintain the reversibility of the reaction and placed in the oven under a certain temperature for several days (from 3 to 7 days). After cooling down to room temperature, the target COF materials can be finally obtained after thoroughly washing the crude powders with organic solvents and dried in a vacuum. It should be noted that because of the limitation of the volume of a Pyrex tube, it is relatively difficult to obtain COF materials at a large scale.

The reaction temperature used in the solvothermal reaction has a significant impact on the properties of COFs, particularly crystallinity. The more commonly used reaction temperatures range from 85 to 120 °C. For instance, B–O-linked COF-6, COF-8, COF-10, COF-102, COF-103, COF-105, and COF-108 can be obtained at 85 °C.^73,96^ Most COFs based on the Schiff-base reaction that form the C=N bond usually react at 120 °C.^78^ In some cases, higher temperatures, such as 160 °C (PI-COF-4 and PI-COF-5),^97^ 200 °C (PI-COF-1 and PI-COF-2),^98^ and even 250 °C (PI-COF-3),^98^ were adopted for the synthesis of polyimide-based COFs.

**Fig. 9:**
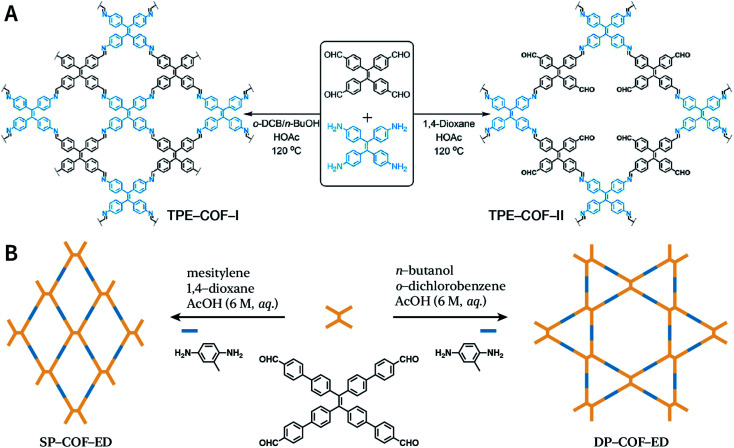
Solvent-induced constitutional isomerism of COFs. (A) Preparation of TPE-COF-I with conventional [4 + 4] pathway and TPE-COF-II with unusual [4 + 2] pathway. Adapted with permission.^100^ Copyright 2018, American Chemical Society. (B) Synthesis of SP-COF-ED with square monoporous structure and DP-COF-ED with triangular and hexagonal dual-porous structures.

Another key factor affecting the synthesis of COFs is the solvent, such as mesitylene, 1,4-dioxane, *o*-dichlorobenzene, and *n*-butanol. It is necessary to try various solvents with different ratios one by one during synthesis to optimize the synthesis conditions. In 2011, Jiang *et al.* synthesized porphyrin-based COF of ZnP-COF;^99^ further, they found that when mesitylene and 1,4-dioxane were used as the solvents, the ratio of these two solvents significantly affected the crystallinity and micromorphology of ZnP-COF. More importantly, apart from the crystallinity of COFs, the solvent may also affect the structures of the COF materials. An interesting example is TPE-COF-I and TPE-COF-II (Fig. 9A) based on a tetraphenylethene core.^100^ When using *o*-dichlorobenzene/*n*-butanol/acetic acid (15 : 15 : 2, v/v/v) as the solvent, the conventional [4 + 4] pathway of 4,4′,4′′,4′′′-(ethene-1,1,2,2-tetrayl)tetraaniline and 4,4′,4′′,4′′′-(ethene-1,1,2,2-tetrayl)tetrabenzaldehyde affords TPE-COF-I with a fully bonded network. However, using 1,4-dioxane/acetic acid (15 : 1, v/v) for the synthesis reaction, an unusual [4 + 2] pathway leads to TPE-COF-II with unreacted *trans*-position aldehyde groups. Recently, Zhao *et al.* reported solvent-induced COF isomerization (Fig. 9B).^101^ In particular, the reaction of 2-methylbenzene-1,4-diamine and 4′,4′′′,4′′′′′,4′′′′′′′-(ethene-1,1,2,2-tetrayl)tetrakis(([1,1′-biphenyl]-4-carbaldehyde)) in different solvents (mesitylene/1,4-dioxane or *o*-dichlorobenzene/*n*-butanol) formed SP-COF-ED with a single-pore structure and DP-COF-ED with a heteropore structure. Interestingly, these two COFs exhibited significantly different adsorption behaviors toward *n*-hexane, and *in situ* structural transformation from DP-COF-ED to SP-COF-ED could be realized by the reaction of DP-COF-ED with 2-methylbenzene-1,4-diamine for 3 days.

**Fig. 10:**
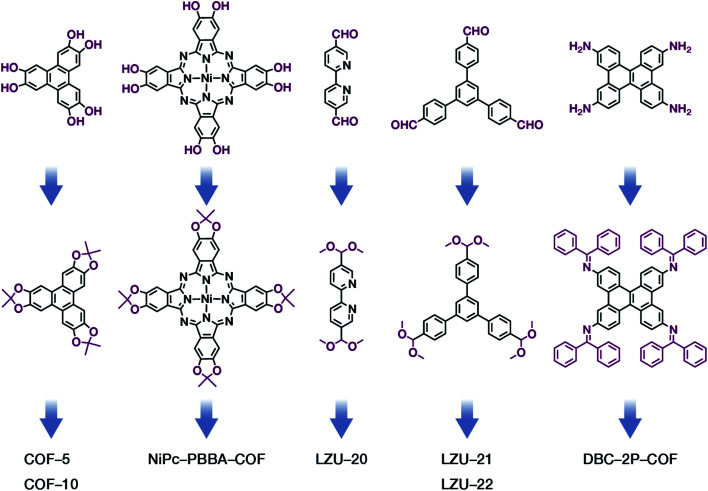
Thermodynamically controlled synthesis of B–O- and C=N-linked COFs using acetals and Schiff base.

The formation of COFs is essentially a thermodynamic control reaction. Therefore, in order to avoid the formation of amorphous polymers under the rapid reaction between monomers, the reaction sites in the monomers can be protected in advance with the protecting groups (Fig. 10). Through this strategy, the reaction rate can be reduced, and it is relatively easier to obtain highly crystalline COF materials. This strategy has been proven by the successful syntheses of COF-5,^102^ COF-10,^102^ NiPc-PBBA-COF,^102^ LZU-20,^103^ LZU-21,^103^ LZU-22,^103^ and DBC-2P COF.^104^ In addition, propylamine-protected 2,4-dihydroxybenzene-1,3,5-tricarbaldehyde was used as a precursor for COF synthesis,^105^ and propylamine inhibited the lateral growth of COF sheets, thereby affording hexagonal COF mesocrystals with rod-like morphology.

**Fig. 11:**
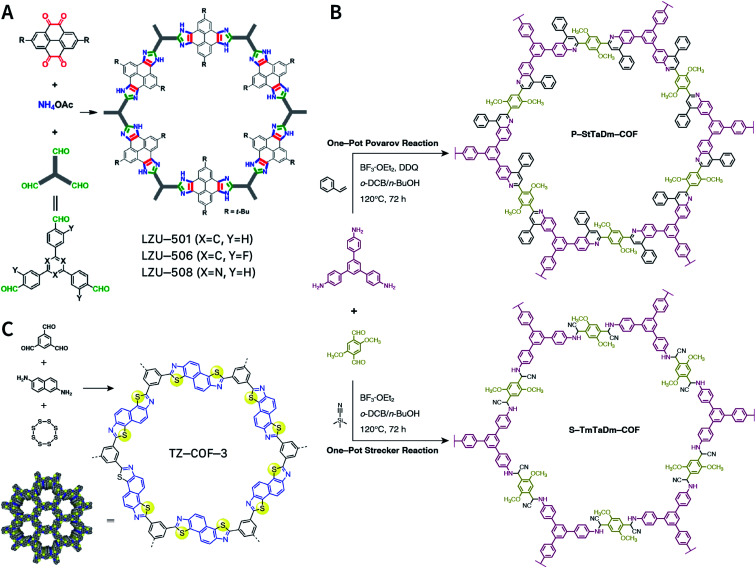
Construction of robust COFs *via* multicomponent one-pot reactions. (A) Synthesis of imidazole-linked COFs *via* a Debus–Radziszewski imidazole synthesis reaction. Adapted with permission.^106^ Copyright 2019, American Chemical Society. (B) Synthesis of quinoline- and α-aminonitrile-linked COFs *via* Strecker and Povarov reactions. (C) Synthesis of thiazole-linked COFs *via* C–H functionalization and oxidative annulation reactions. Adapted with permission.^108^ Copyright 2020, American Chemical Society.

Multicomponent reactions have recently proven to be an effective way to optimize solvothermal thermodynamics and kinetics. They combine a reversible covalent bond to allow the crystallinity of COFs and an irreversible covalent bond that imparts stability, reaching a higher level of complexity and precision in covalent assembly. Recently, Wang's and Dong's groups reported the applications of this strategy for COF synthesis. As shown in Fig. 11A, the three-component one-pot Debus–Radziszewski reaction among pyrene-4,5,9,10-tetraone, aromatic trialdehydes, and ammonium acetate afforded a series of imidazole-linked COFs under solvothermal conditions,^106^ and five covalent bonds in each cyclic joint were formed *in situ* during polymerization. Fig. 11B shows that when a mixture of 1,3,5-tris(4-aminophenyl)benzene, 2,5-dimethoxyterephthalaldehyde, and phenylethylene was heated in *o*-dichlorobenzene/*n*-butanol (1 : 1, v/v) at 120 °C for 72 h in the presence of BF_3_·OEt_2_, 4,5-dichloro-3,6-dioxocyclohexa-1,4-diene-1,2-dicarbonitrile (DDQ), and acetic acid, the one-pot *in situ* Povarov reaction afforded P-StTaDm-COF as an orange-red crystalline solid.^107^ Similarly, by replacing trimethylsilane carbonitrile with phenylethylene in the aforementioned reaction system, S-TmTaDm-COF could be obtained *via* the three-component *in situ* Strecker reaction.^107^ In addition, based on the report by Cooper *et al.*, benzothiazole-linked COFs^108^ were obtained by adding sulfur to the conventional synthesis system of Schiff-base COFs (Fig. 11C). The reversible imine condensation, irreversible C–H functionalization reaction, and oxidative annulation reaction synergistically afforded a set of TZ-COFs with high crystallinity and excellent robustness. This *in situ* multicomponent polymerization approach might open a new avenue for constructing COFs that are not possible to be successfully obtained by other conventional methods.

### 3.2 Microwave synthesis

Microwave synthesis^109^ is related to the synthesis approach using microwave heating. When compared with the traditional external heating method, microwave heating is endogenous, that is, the object to be heated is a heat-generating object; further, it does not require heat conduction, and uniform heating can be achieved in a short time.

In 2009, Cooper *et al.* were the first to use microwaves to synthesize B–O-linked COF-5 with a high yield of 68%.^110^ The reaction time (about 20 min) by microwave heating is more than 200 times faster than that of solvothermal synthesis (3 days). Meanwhile, the BET specific surface area of the obtained COF-5 is higher than that of previously reported COFs under solvothermal conditions (2019 *vs.* 1590 m^2^ g^−1^). Imine-linked TpPa-COF was also synthesized by microwave heating using benzene-1,4-diamine and 2,4,6-trihydroxybenzene-1,3,5-tricarbaldehyde as the monomers.^111^ Dichtel *et al.* further synthesized imine- and β-ketoenamine-linked COFs by microwave synthesis using benzophenone *N*-aryl imine with higher solubility and stronger oxidation stability to replace the aromatic amine in the imine condensation reaction.^112^ Very recently, dioxin-linked DH-COF was synthesized under microwave conditions within 30 min using the nucleophilic substitution reaction of 2,3,5,6-tetrafluoroisonicotinonitrile with triphenylene-2,3,6,7,10,11-hexaol.^113^

These examples clearly indicate that microwave heating can immensely increase the reaction rate and shorten the reaction time. Meanwhile, microwave synthesis enables online monitoring, which is significantly difficult to be achieved for solvothermal synthesis.

### 3.3 Ionothermal synthesis

Ionic liquids (ILs) are a class of organic salts that are liquid at room temperature or near room temperature.^114^ As recyclable alternatives to traditional volatile organic solvents, ILs have been widely used as environment-friendly solvents. The synthesis reaction carried out in ILs is referred to as ionothermal synthesis, and it has exhibited great promise for industrial applications due to the avoidance of safety hazards caused by pressure.

**Fig. 12:**
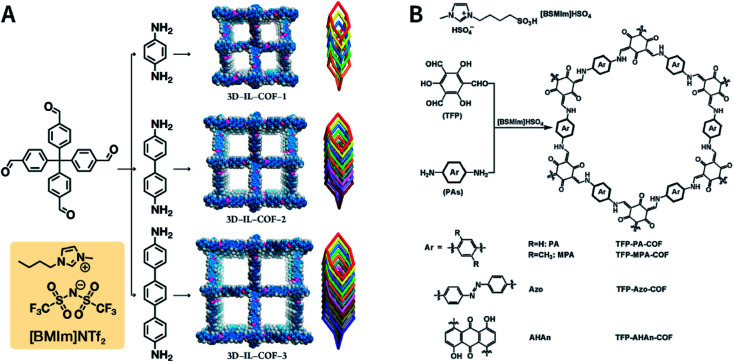
Ionothermal synthesis of COFs. (A) Synthesis of 3D COFs in [BMIm]NTf_2_ IL. Adapted with permission.^115^ Copyright 2018, American Chemical Society. (B) Synthesis of β-ketoenamine-linked COFs in [BSMIm]HSO_4_ IL. Adapted with permission.^116^ Copyright 2019, Elsevier B.V.

In 2018, 1-butyl-3-methylimidazolium bis(trifluoromethylsulfonyl)imide ([BMIm]NTf_2_) was chosen as a solvent as well as a catalyst for producing Schiff-base COFs.^115^ In the ILs, the tetrahedral monomer of 4,4′,4′′,4′′′-methanetetrayltetrabenzaldehyde reacted with the linear monomers of gradually increasing lengths, namely, *p*-phenylenediamine (6 Å), [1,1′-biphenyl]-4,4′-diamine (11 Å), and [1,1′:4′,1′′-terphenyl]-4,4′′-diamine (15 Å), to produce 3D-IL-COF-1, 3D-IL-COF-2, and 3D-IL-COF-3 with interpenetrated dia structures, respectively (Fig. 12A). By using ILs, the reaction time was shortened from 72 to 12 h. This is the first report on COF synthesis using ILs. Unfortunately, ILs are rather difficult to be removed from the pores of 3D COFs, thereby limiting their widespread applications.

The synthesis of COFs that do not contain ILs as the guest in the pores was reported for the first time by Kang *et al.* in 2019.^116^ By using 1-(4-sulfobutyl)-3-methylimidazolium hydrogen sulfate ([BSMIm]HSO_4_) as the solvent as well as catalyst, four β-ketoenamine-linked COFs were obtained at ambient pressure (Fig. 12B). Due to the open channels of COFs, [BSMIm]HSO_4_ could be completely removed from the pores, and it could be recycled for further use without any activity loss. In the same year, imine-linked TFPPy-PDA-COF^117^ and TPB-DMTP-COF^118^ were also synthesized using [BMIm]NTf_2_ and [BSMIm]HSO_4_ ILs, respectively.

In addition, ILs [C_*n*_mim][BF_4_] (*n* = 4, 6, 10) with alkyl chains of different lengths have also been used for the ionothermal synthesis of imine- and hydrazone-linked COFs.^119^ Apart from the inherent structural pores, alkyl chains with different lengths enable the induction of mesopores with different porosities, thereby exhibiting excellent performance in catalyzing C–C coupling reactions.

### 3.4 Atmospheric solution synthesis

**Fig. 13:**
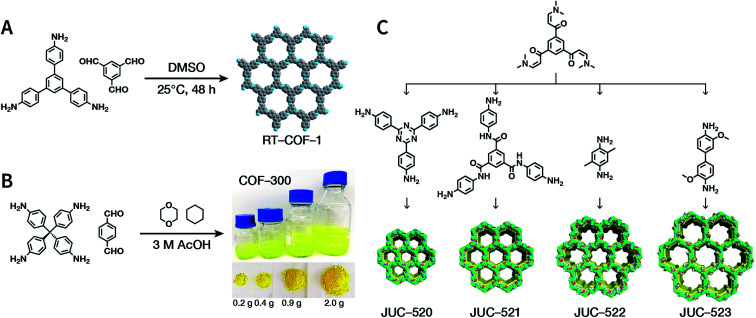
Atmospheric solution synthesis of COFs. (A) Room-temperature imine condensation reaction to form RT-COF-1. (B) Synthesis of COF-300 in jars at the gram scale. Adapted with permission.^121^ Copyright 2019, American Chemical Society. (C) Ambient aqueous-phase synthesis of JCU-520, JUC-521, JUC-522, and JUC-523 based on the Michael addition–elimination reaction of β-ketoenamine and arylamine. Adapted with permission.^122^ Copyright 2019, The Royal Society of Chemistry.

The importance of achieving COF synthesis under ambient pressure is self-evident. The first example of COF synthesis at atmospheric pressure and room temperature was reported by Zamora *et al.* in 2015.^120^ Benzene-1,3,5-tricarbaldehyde and 1,3,5-tris(4-aminophenyl)benzene were used as the monomers and stirred in DMSO for 48 h to obtain a white powder of RT-COF-1 (Fig. 13A). This milestone work opened new avenues for the large-scale production of COFs. Furthermore, COF-300 has also been obtained under atmospheric pressure. As shown in Fig. 13B, with 1,4-dioxane and cyclohexane as the reaction solvents, a gram-scale yellow powder of COF-300 was obtained at 65 °C with a yield of up to 90%.^121^ The crucial factors for obtaining high-quality crystalline imine-linked COFs mainly include lower temperature (to prevent the oxidation of –NH_2_), reduction in water within the reaction system (to maintain reversibility of imine condensation), and reduction in solubility (to control nucleation and continuous crystal growth).

Recently, Fang *et al.* reported the atmospheric pressure synthesis of COFs based on the Michael addition–elimination reaction in aqueous solutions.^122^ Typically, β-ketoenamine and arylamine were suspended in an aqueous solution containing acetic acid as the catalyst, followed by maintaining the reaction at ambient temperature and pressure to produce crystalline JUC-520, JUC-521, JUC-522, and JUC-523 solids (Fig. 13C). More importantly, by scaling up, it took only 30 min to afford gram-scale JUC-521 with a yield of up to 93%. This eco-friendly, low-cost, and mild synthesis method provides the possibility of large-scale production of COFs. To give the readers a better understanding of this promising approach, detailed examples of COF syntheses in an atmospheric solution are summarized in Table 1.^120–134^

**Table 1:** Synthesis of COFs in an atmospheric solution

COFs	Monomers	Reaction condition	Ref.
TFB-HZ COF, TFP-HZ COF	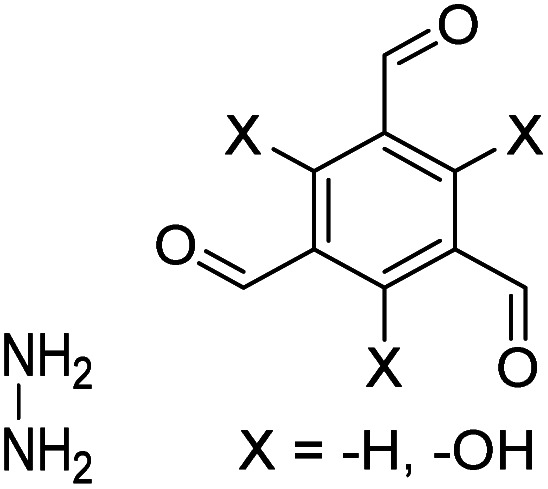	1,4-Dioxane/mesitylene (1 : 1, v/v), 25 °C, 72 h	123
HZ-BTCA COF	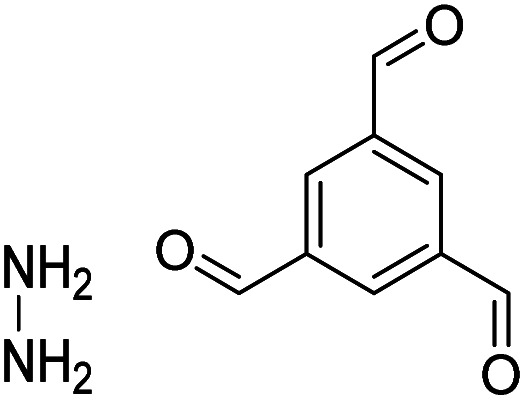	H_2_O, 80 °C, 120 h	124
N_3_-COF, TFPB-HZ COF	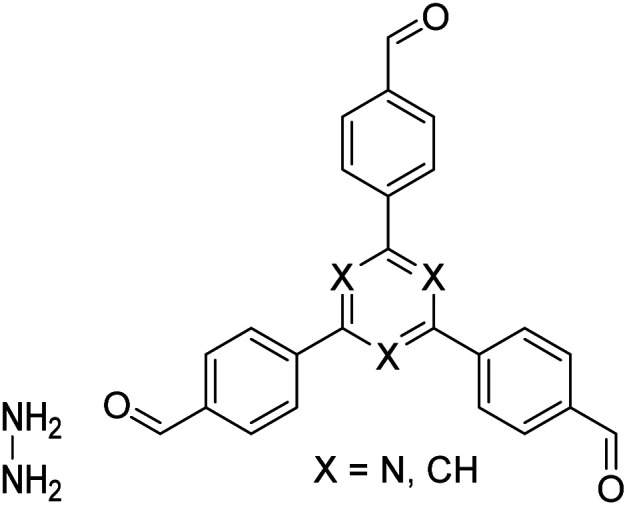	1,2-Dichlorobenzene/ethanol (2 : 3, v/v), 25 °C, 72 h	123
NUS-14, NUS-15	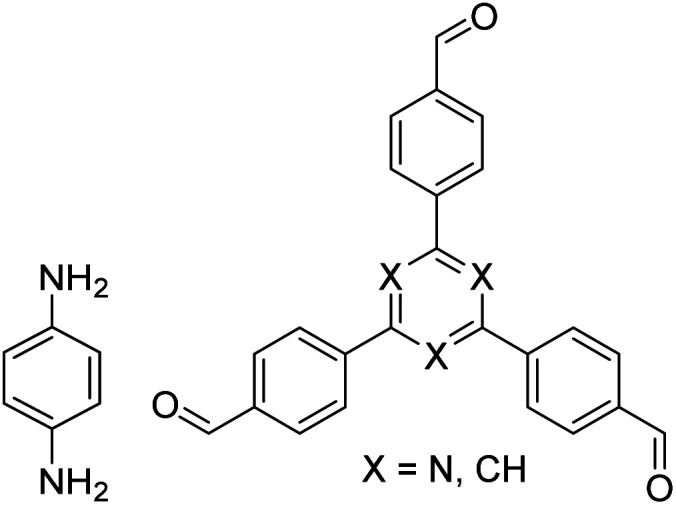	Mesitylene/ethanol (1 : 1, v/v), 25 °C, 72 h	123
TpPa-1	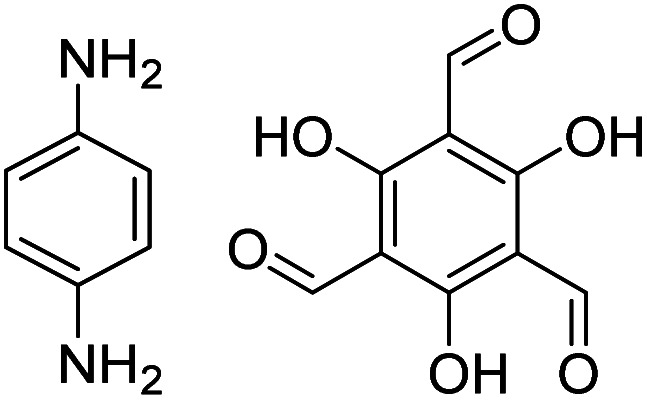	1,4-Dioxane, 25 °C, 72 h	123 and 125
COF-LZU1	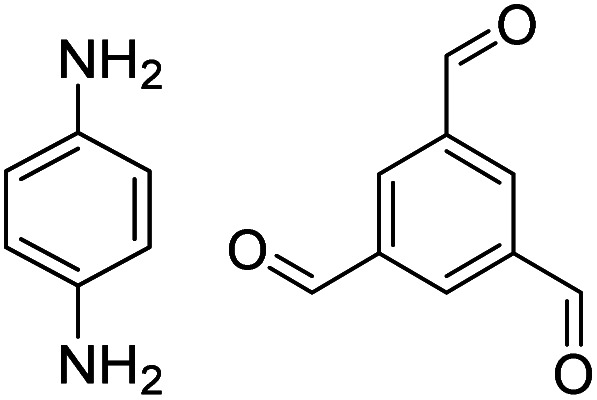	1,4-Dioxane or ethanol, 20 °C, 72 h	126
TpBD COF	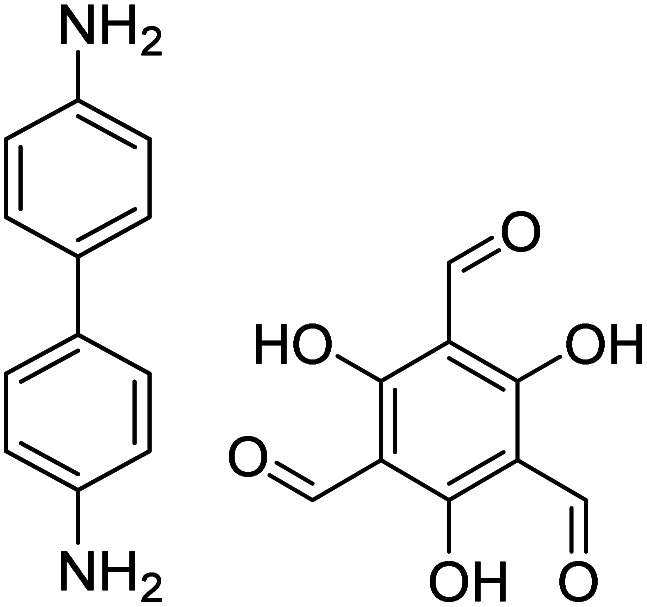	Ethanol, 25 °C, 30 min	127
TPB-DMTP-COF	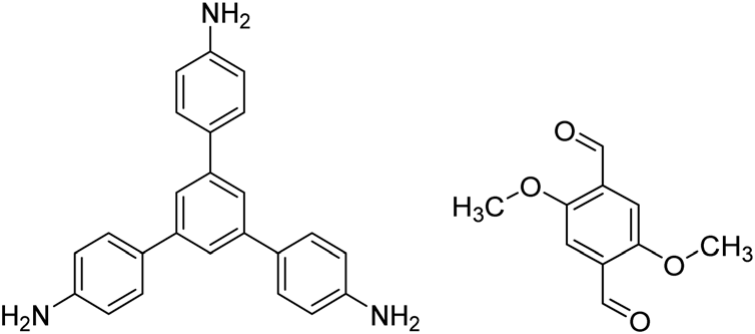	1,4-Dioxane/mesitylene (1 : 1, v/v), 25 °C, 72 h	128
TAPB-PDA, TAPB-OHPDA, TAPB-OMePDA	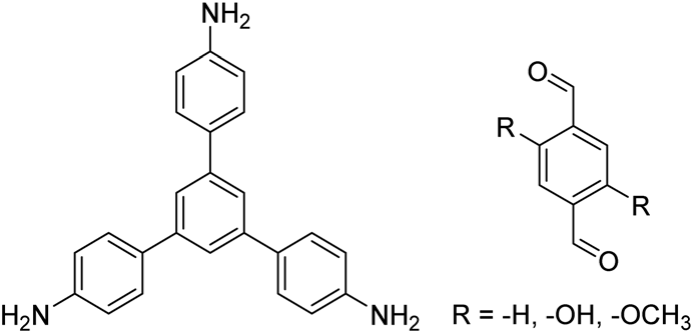	1,4-Dioxane/mesitylene (4 : 1, v/v), 70 °C, 4 h	129
TzDa COF	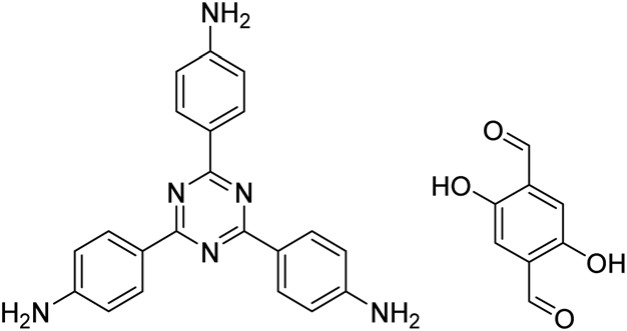	1,2-Dichlorobenzene/ethanol (1 : 1, v/v)	130
TAPB-BPDA	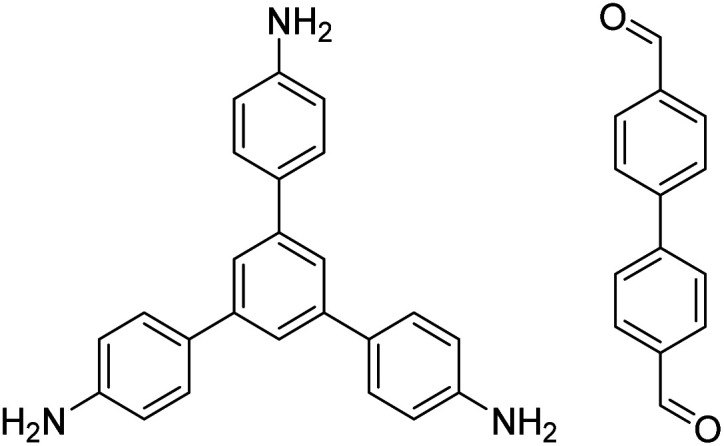	1,4-Dioxane/mesitylene (4 : 1, v/v), 20 °C, 30 min	131
RT-COF-1	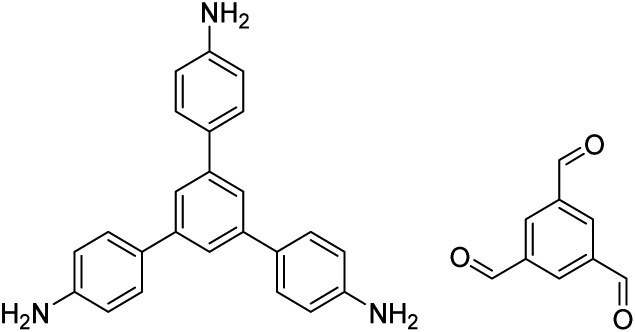	DMSO or 3-methylphenol, 25 °C, 48 h	120, 132 and 133
TZ-BTCA-COF, TAPB-BTCA-COF	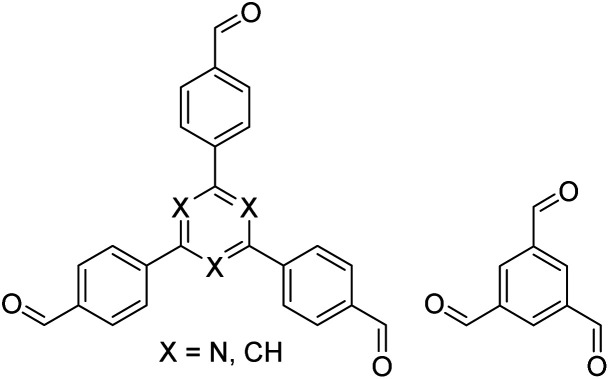	H_2_O, 80 °C, 120 h	124
TAPPy-PDA COF	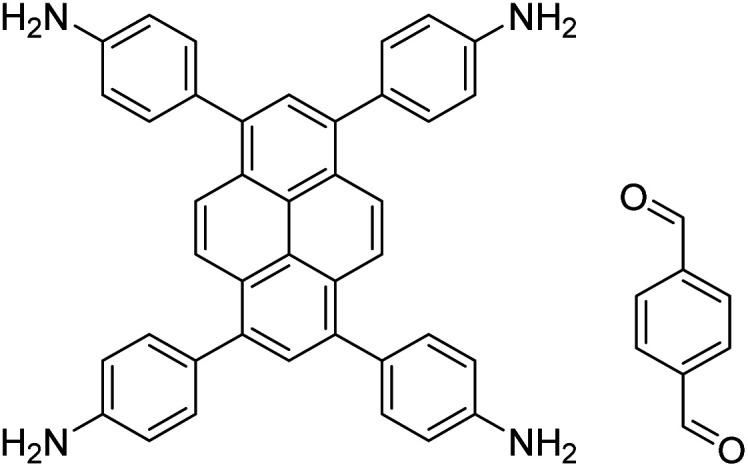	1,4-Dioxane/mesitylene (4 : 1, v/v), 70 °C, 4 h	129
Tf-DHzOPr, Tf-DHzOAll, Tf-DHzOBz	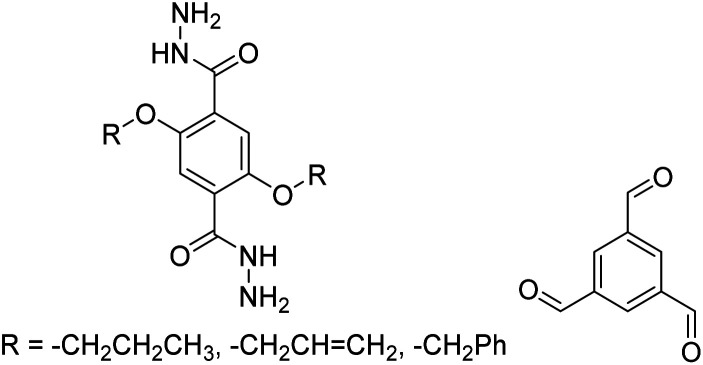	1,2-Dichlorobenzene, 100–120 °C, 30 min	134
COF-42, Pr-COF-42	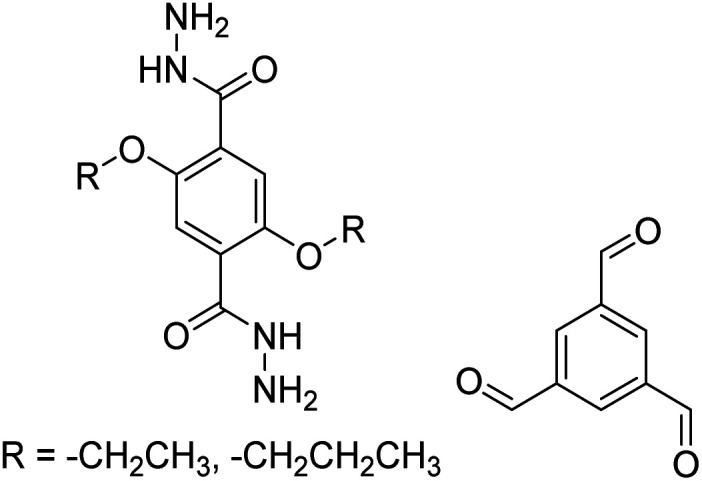	1,4-Dioxane/mesitylene (3 : 2, v/v), 20 °C, 72 h	126
COF-43	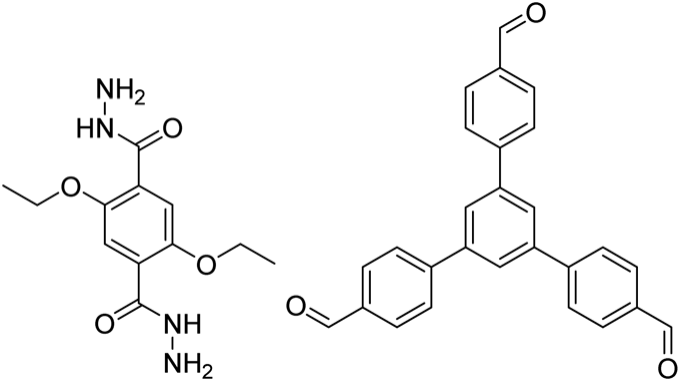	1,4-Dioxane/mesitylene (1 : 3, v/v), 20 °C, 72 h	126

### 3.5 Mechanochemical synthesis

**Fig. 14:**
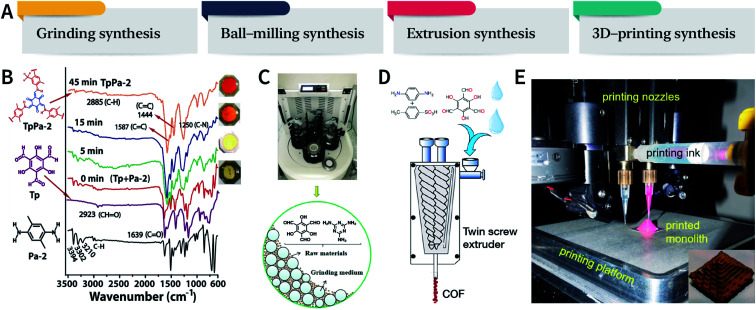
(A) Mechanochemical synthesis of COFs. (B) Grinding synthesis of TpPa-2 COF. Stepwise comparison of the FT-IR spectra showing the reaction progress with time for TpPa-2. Inset: color changes observed during grinding. Adapted with permission.^137^ Copyright 2013, American Chemical Society. (C) Ball-milling synthesis of COFs. Adapted with permission.^138^ Copyright 2019, Elsevier B.V. (D) Extruder synthesis of COFs. Adapted with permission.^139^ Copyright 2017, American Chemical Society. (E) 3D printing technology used for COF preparation and molding. Inset: a delicate COF device. Adapted with permission.^140^ Copyright 2019, American Chemical Society.

Mechanical chemistry research originated from the transformation of mechanical energy and chemical energy in biochemistry related to physiological functions. At present, mechanochemistry mainly refers to the process of applying mechanical energy to substances *via* squeezing, shearing, and friction to induce chemical changes between solids.^135^ With the development of the machinery industry, the continuous emergence of various high-energy grinding equipment has enabled the application of mechanical chemistry in many fields such as metal alloying, inorganic materials, organic synthesis, and compound modification.^136^ So far, at least four types of mechanical synthesis equipment have been developed for COF preparation and molding, namely, mortar, ball mill, extruder, and 3D printer (Fig. 14A). Mechanochemical synthesis is also considered as a green synthesis process due to its obvious characteristics of no/low solvents.

In 2013, TpPa-1, TpPa-2, and TpBD COFs were synthesized by means of solvent-free mechanochemical grinding using aldehyde–amine condensation reactions.^137^ In short, the raw materials were placed in an agate mortar and ground at room temperature. After about 5 min, the color of the powder changed to light yellow and gradually turned into orange within 15 min. After 45 min of grinding, the powder became crimson in color, suggesting the successful formation of COFs (Fig. 14B). When compared with the classic solvothermal method, this method is fast, controllable, and environment-friendly; however, the crystallinity of the resulting COFs is normally unsatisfactory. The BET specific surface area is only 61 m^2^ g^−1^ for TpPa-1 COF, 56 m^2^ g^−1^ for TpPa-2 COF, and 35 m^2^ g^−1^ for TpBD COF. In contrast, the COFs synthesized by the solvothermal method afforded BET surface areas of 535, 339, and 537 m^2^ g^−1^ for TpPa-1, TpPa-2, and TpBD COFs, respectively.

In order to further improve the synthesis efficiency, grinding can be performed in a ball mill. According to the report of Banerjee *et al.*, when the frequency of the ball mill with two 7 mm-diameter stainless steel balls is 25 Hz, the yield of TpPa-1 COF can reach 90% at 45 min.^137^ Besides, ball milling can also be used to synthesize Tp-MA COF (Fig. 14C) by the reaction of 2,4,6-trihydroxybenzene-1,3,5-tricarbaldehyde and melamine at ambient temperature.^138^ The resulting material can be used for degrading various types of organic pollutants.

The third method of mechanochemical synthesis of COFs is the extrusion process. A twin-screw extruder has been used for the continuous synthesis of COFs.^139^ In a representative synthesis process, *p*-phenylenediamine and solid catalyst of *p*-toluenesulfonic acid were mixed in a beaker, manually fed into the extruder, and then 2,4,6-trihydroxybenzene-1,3,5-tricarbaldehyde and a small amount of water were sequentially added; after mixing for a specific period of time, the mixture was heated at 170 °C for 1 min. Highly crystalline and porous COF materials were obtained after washing and drying (Fig. 14D). This approach provides the possibility of large-scale production of COFs at high throughputs of several kilograms per hour.

More recently, 3D printing technology has also been employed for the preparation and molding of COFs.^140^ With the help of a 3D printing template, Pluronic F127 and the raw materials were mixed and subsequently forming hydrogels; then, the COFs could be printed using commercial 3D printers (Fig. 14E). Due to the extremely controllable and operational accuracy of 3D printing, very delicate COF devices could be obtained.

The mechanochemical synthesis of COFs is still at a vigorously developing stage. The advantage of space–time yield overwhelms the lack of relatively high crystallinity, and the resulting COFs have been applied in the fields of separation,^141^ detection,^142^ and electrochemistry.^143,144^

### 3.6 Other synthesis methods

In addition to the aforementioned methods, other synthesis methods have also been developed, such as photochemical synthesis,^145,146^ electron-beam irradiation synthesis,^147^ and vapor-assisted synthesis.^148^ Although these methods are probably not universal, they might be highly effective for the synthesis of task-specific COFs with specific structures. Among these novel methods, it is particularly worth mentioning the efforts to synthesize hcc-COF by Choi *et al.*^146^ Benzene-1,2,4,5-tetraamine, cyclohexane-1,2,3,4,5,6-hexaone, water, and acetic acid were mixed in a quartz bottle and exposed to simulated sunlight (∼200–2500 nm, 50 mW cm^−2^) irradiation for 3 h to produce hcc-COF. Light energy not only accelerates the imine condensation reaction, but also promotes the conversion reaction from amorphous polyimide precipitation to crystalline COFs *via* fast and reversible dynamic imine condensation. As a comparison, the reaction in darkness for only 3 h formed an amorphous product.

## 4 Nanocrystallization of COFs

**Fig. 15:**
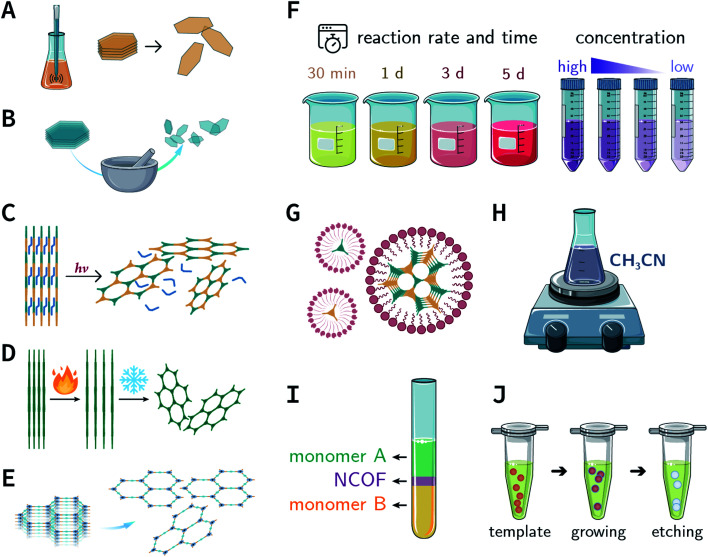
Synthesis of NCOFs by the top-down and bottom-up strategies. (A) Ultrasonic exfoliation. (B) Mechanical exfoliation. (C) Chemical exfoliation. (D) Gas-driven exfoliation. (E) Charge-mediated self-exfoliation. (F) Reaction kinetics regulation. (G) Surfactant-assisted synthesis. (H) Acetonitrile method. (I) Interfacial synthesis. (J) Template method.

In the earlier section, we discussed the general synthesis approaches for fabricating bulk COFs. However, due to the strict restrictions on the size of the materials for biomedical applications, micron-sized bulk COFs cannot be directly applied in the field of oncology. In this section, we will systematically discuss how to obtain NCOFs to meet the needs of biomedical applications. It should be noted that as long as one spatial dimension of the COF material is in the nanoscale, then it can be classified as NCOF, including, but not limited to, quantum dots, nanorods, nanosheets, and nanoparticles. The process of preparing NCOFs is also called COF nanocrystallization. Similar to other 2D materials,^18,57^ according to the differences in raw materials, COF nanocrystallization can be divided into two categories (Fig. 15). One is the top-down method, which uses bulk COFs as the precursor, which destroys the interlayer interaction of the COFs under certain conditions to afford nanosheets. The other is the bottom-up method, which employs the corresponding monomers to directly synthesize NCOFs.

### 4.1 Top-down synthesis

#### 4.1.1 Ultrasonic exfoliation

Ultrasonic exfoliation^149^ is a universally applicable and commonly used nanocrystallization strategy, theoretically applicable to all types of COFs. The common practice is that bulk COFs are suspended in a specific solvent with the appropriate surface energy and then simply sonicated. During this process, ultrasound induces bubbles in the solvent; when the bubbles burst due to their huge surface tension, shock waves are induced across the bulk COF surfaces that destroy the interlayer π–π-stacking interactions of the COFs, thereby affording exfoliated nanosheets.

The solvents are significant for the process of ultrasonic exfoliation. Appropriate solvents can promote exfoliation as well as inhibit aggregation. Generally, polar solvents (*e.g.*, ethanol, water, and 1,4-dioxane) can be used to obtain better exfoliation effectivity.^150^ Theoretical studies have indicated that the Hansen's parameter (HSP) of the solvent—a metric that determines the intensity of the molecular forces in the solvent—may be a useful reference for selecting the ultrasonic exfoliation media.^151^ Some examples for the preparation of NCOFs by ultrasonic exfoliation are shown in Table 2.^150–165^

**Table 2:** Ultrasonic exfoliation to prepare COF nanosheets

COFs	Ultrasonication	Nanosheet size	Application	Ref.
COF-43	1,4-Dioxane	Thickness of 1.32 ± 0.37 nm	Not mentioned	150
RIO-1	47 kHz, 2 h, ethanol	Thickness of 10.7 ± 3.2 nm	Not mentioned	151
TPA-COF	110 W, 40 kHz, 3 h, ethanol	Thickness of 3.5 ± 0.3 nm	DNA detection	152
PI-COF	8 h, in ethanol	Thickness of 1 nm	2,4,6-Trinitrophenol detection	153
COF-LZU1	110 W, 40 kHz, 4 h, ethanol	Thickness of 3.51 nm	DNA detection	154
JUC-510/511/512	7 h, isopropanol	Thickness of 36, 22, and 19 nm	Electrochemical double-layer capacitor	155
TTA-DFP COF	20 kHz, water	Thickness of 1.0–1.3 nm	Cell nucleus bioimaging	156
Bpy-COF/BD-COF	650 W, 25 kHz, 8 h, water	Thickness of 1 and 1.5 nm	Al^3+^ fluorescence sensing	157
TpASH-NPHS	130 W, 20–25 kHz, 6 h, water	Size of 87.4 ± 18.6 nm, thickness of 1.6 ± 0.3 nm	H_2_S fluorescence bioimaging	158
NDI-COF	3 h, water	Thickness of 6–7 nm	Oxygen reduction electrocatalyst	159
Tp-Bpy COF	6 h, water	Not mentioned	Hg^2+^ colorimetric detection	160
TP-Por COFs	450 W, 19–25 kHz, 2 h, PBS	Hydrodynamic diameter of 350 nm	Chemo-photothermal tumor therapy	161
TP-Por COFs	450 W, 19–25 kHz, 2 h, PBS	Thickness of 25 nm	Photodynamic therapy	162
TphDha COF	300 W, 5 h; 1200 W, 5 h, DMF/water (9 : 1, v/v)	Thickness of about 3 nm	Photodynamic therapy	163
COF_DOPA_	50 kHz, 90 min, CHCl_3_	Lateral dimension of 100 nm	Non-migrating antioxidant materials	164
sp^2^c-COF	250 W, 30 min	Not mentioned	Luminescent materials	165

**Fig. 16:**
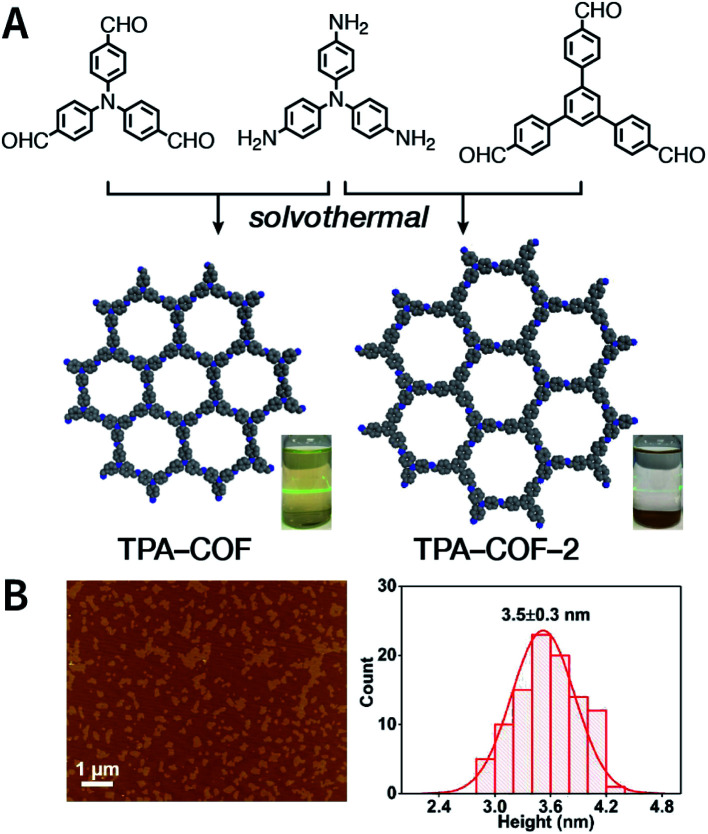
(A) Syntheses of TPA-COF and TPA-COF-2. Inset: photograph of the Tyndall effects in TPA-COF and TPA-COF-2 nanosheet suspensions obtained under the same ultrasonic processing conditions. (B) Statistical analysis of the thickness measured by AFM of TPA-COF-2 nanosheets. Adapted with permission.^152^ Copyright 2017, American Chemical Society.

Evidently, the weaker the interactions between the COF layers, the easier it becomes to perform ultrasonic exfoliation. TPA-COF constructed with tris(4-aminophenyl)amine and tris(4-formylphenyl)amine flexible monomers with the *C*_3v_ symmetry confirmed this prediction.^152^ Bulk TPA-COF can be synthesized by the solvothermal method in 1,2-dichlorobenzene/ethanol/3 M acetic acid (20 : 5 : 1, v/v/v). Bulk TPA-COF was sonicated in an ultrasonic bath (110 W, 40 kHz, ethanol) for 3 h, which was naturally sedimented for 24 h to obtain high-quality nanosheets in the supernatant liquid (Fig. 16). If the tris(4-formylphenyl)amine in TPA-COF was replaced with 1,3,5-tris(4-formylphenyl)benzene, the resulting TPA-COF-2 was relatively difficult to exfoliate into nanosheets under similar conditions. This interesting phenomenon can be attributed to the approximately planar structure and strong π-delocalized system of 1,3,5-tris(4-formylphenyl)benzene, resulting in the increasing π–π interactions between the adjacent layers in TPA-COF-2 as compared to those in TPA-COF.

Unfortunately, ultrasonic exfoliation is a very time- and energy-consuming process, and it is challenging to prepare a large number of NCOF sheets within a short time. More importantly, the size of the nanosheets obtained by ultrasonic exfoliation is often uneven. Therefore, proper postprocessing is necessary, such as removing the large COF particles that have not been completely peeled by standing or low-speed centrifugation. In some cases, filtration is also a feasible option.

#### 4.1.2 Mechanical exfoliation

Mechanical exfoliation mainly refers to the method of exfoliating bulk COFs by grinding, which is widely used for the preparation of 2D nanomaterials.^166^ During grinding, the grinding medium exerts impact, friction, and shear force on the COF powders *via* regular or irregular movements, causing crystallographic slips between the different layers and subsequently resulting in flaking. Based on the differences in the media, mechanical exfoliation can be divided into wet grinding and dry grinding: the difference between these two methods is the necessity to add a solvent. Generally, wet grinding yields better exfoliating efficiency.

**Fig. 17:**
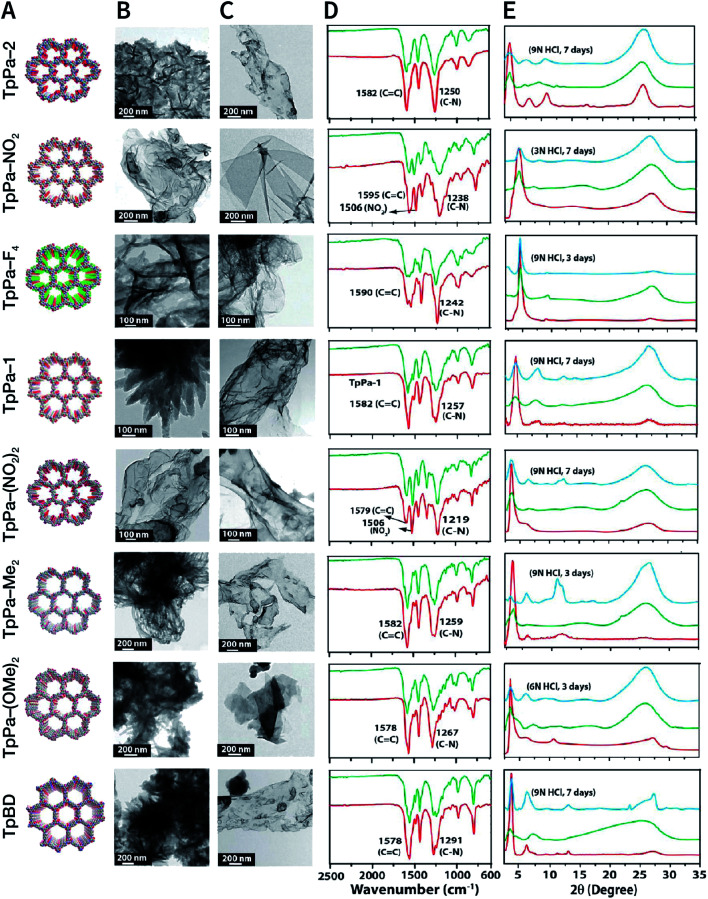
COF nanosheets prepared by mechanical exfoliation. (A) Packing diagrams (B) HRTEM images of bulk COFs. (C) HRTEM images of delaminated COF nanosheets. (D) FT-IR spectra of bulk COFs (red) and corresponding COF nanosheets (green). (E) PXRD patterns of bulk COFs (red), corresponding COF nanosheets (green), and acid-treated COF nanosheets (cyan). Adapted with permission.^167^ Copyright 2013, American Chemical Society.

NCOF sheets prepared by mechanical exfoliation were reported for the first time by Banerjee *et al.* in 2013.^167^ They synthesized eight Schiff-base COFs with different pore diameters based on the imine condensation reaction (Fig. 17A), and manually ground them in an agate mortar for about 30 min in the presence of a small amount of methanol. After removing the remaining bulk COFs by centrifugation, eight kinds of NCOF sheets (thicknesses ranging from 3 to 10 nm) were obtained with a yield of about 8% (Fig. 17B and C). The obtained NCOF sheets and bulk COF exhibited almost the same FT-IR spectra, confirming that the intrastratal chemical bonds did not change (Fig. 17D). However, in the PXRD pattern, the diffraction peak attributed to the (001) plane broadened, and the diffraction peak corresponding to the (100) plane decreased in intensity, indicating the reduced number of stacked layers and decreased periodicity along the *z*-direction because of random slips in these nanosheets (Fig. 17E). Nevertheless, the resulting nanosheets exhibited good stability in strong acid media.

**Fig. 18:**
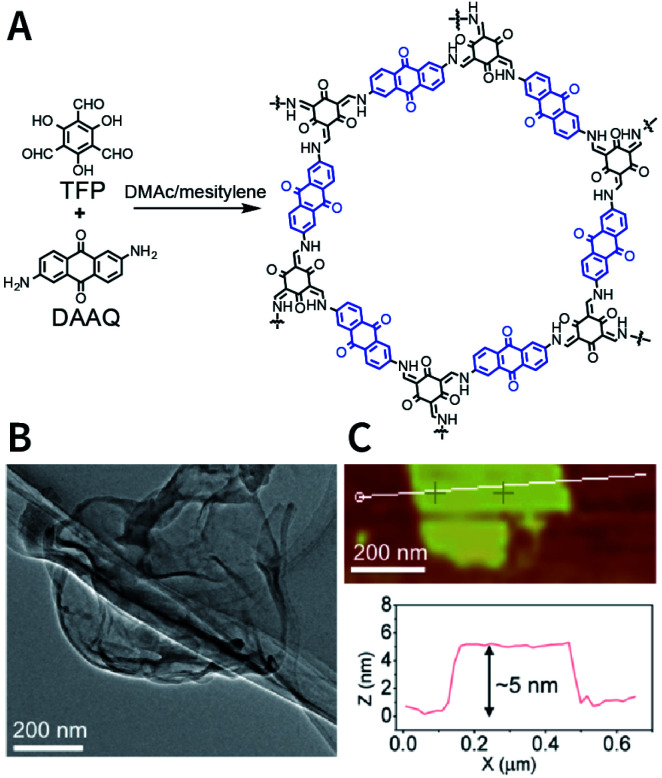
Preparation of DAAQ-TFP-COF nanosheets *via* the dry ball-milling method. (A) Synthesis of bulk DAAQ-TFP-COF. (B) HRTEM images of DAAQ-TFP-COF nanosheets. (C) AFM images of DAAQ-TFP-COF nanosheets. Adapted with permission.^168^ Copyright 2017, American Chemical Society.

The dry ball-milling method has been used for preparing redox-active DAAQ-TFP-COF nanosheets for lithium-ion electrodes.^168^ In a typical synthesis process, bulk DAAQ-TFP-COF was placed in a ball crusher under a vibration frequency of 50 Hz for 0.5 h to prepare DAAQ-TFP-COF nanosheets with a thickness of ∼3–5 nm (Fig. 18). Electrochemical experiments proved that as compared to bulk DAAQ-TFP-COF, exfoliated DAAQ-TFP-COF nanosheets with shorter lithium diffusion pathways yield significantly higher utilization efficiency of redox sites and faster lithium storage kinetics. Moreover, 3BD COF and nanosheets prepared by the dry ball-milling method even show the possibility of fluorescence sensing of peroxide-based explosives.^169^

Since the ball mill has been widely used in industry, NCOF nanosheets prepared by the ball-milling method have been used as modifier additives in the fields of polyurethane modification^170^ and mixed matrix membranes.^171,172^

#### 4.1.3 Chemical exfoliation

The essence of chemical exfoliation is to use chemical reactions to introduce large-sized groups into the COF layer in order to increase the interlayer spacing and weaken the van der Waals force between the layers, thereby inducing exfoliation.

**Fig. 19:**
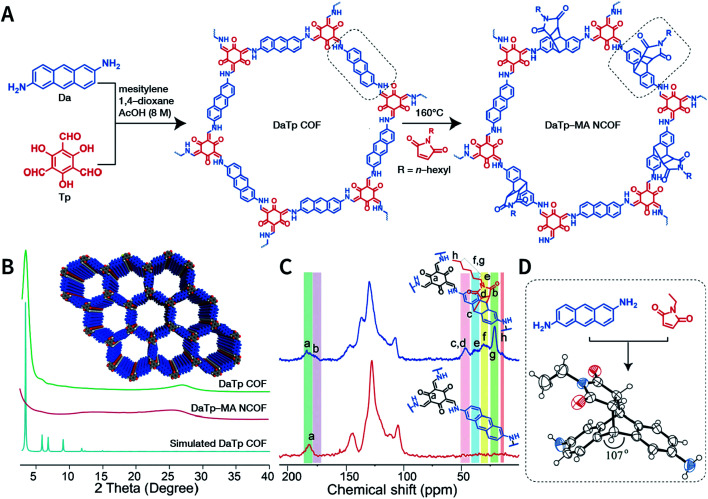
Synthesis of DaTp-MA NCOF sheets through the Diels–Alder cycloaddition-induced chemical exfoliation. (A) Reaction formula of COF preparation and chemical exfoliation. (B) PXRD patterns. (C) ^13^C solid-state NMR spectra of DaTp COF (red) and DaTp-MA NCOF (blue). (D) Model compound synthesized from 2,6-diaminoanthracene and *N*-ethylmaleimide. Adapted with permission.^173^ Copyright 2016, Wiley-VCH Verlag GmbH & Co. KGaA, Weinheim.

Exfoliation induced by the Diels–Alder cycloaddition reaction between *N*-hexylmaleimide and anthracene-based DaTp COF is the earliest report on chemical exfoliation.^173^ The introduction of *N*-hexylmaleimide with a length much larger than the interlayer distance in the COF interferes with the π–π-stacking interaction and planarity of the COF layer, resulting in exfoliation (Fig. 19). Importantly, although both ultrasonic and mechanical exfoliation methods can afford DaTp COF exfoliation, only DaTp-MA NCOF sheets prepared by the chemical exfoliation method can self-assemble in a layer-by-layer manner between the air–water interfaces to produce centimeter-sized films. The authors claimed that this was related to the hydrophobic hexyl groups, which reduced their exposure in the water environment. Since surfactant or stability is not necessary, this method exhibits great promise for various applications. For instance, chemically exfoliated anthracene-based COF nanosheets using maleic anhydride as the functionalizing exfoliation reagent^174^ have been used to enhance the anodic performance of COF materials in lithium-ion batteries.

**Fig. 20:**
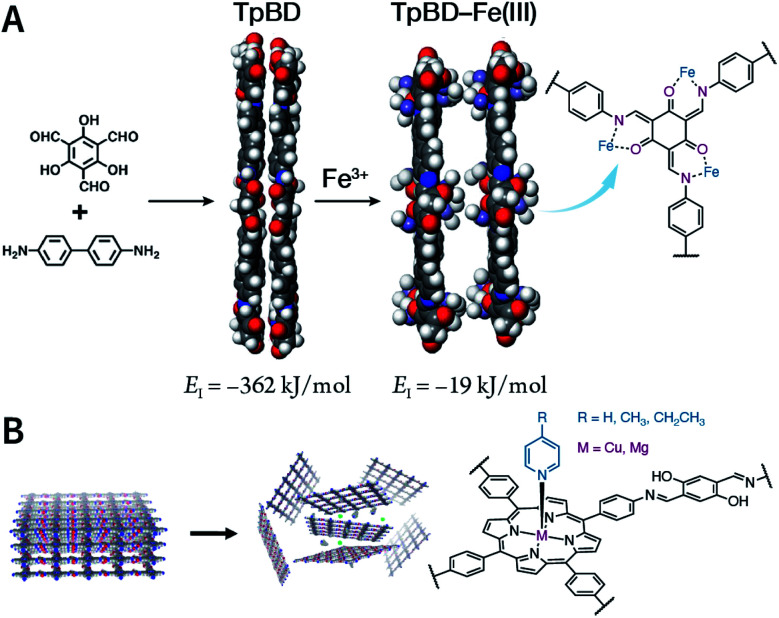
Coordination-induced chemical exfoliation of COFs. (A) Fe^3+^-assisted aqueous-phase chemical exfoliation to prepare TpBD COF nanosheets. Adapted under a Creative Commons Attribution 4.0 International License. Copyright 2018, The Author(s).^175^ Published by Springer Nature Limited. (B) Porphyrin-based nanodisks synthesized *via* the simultaneous axial coordination of pyridines and metal ions. Adapted under a Creative Commons Attribution 4.0 International License. Copyright 2019, The Author(s).^176^ Published by Springer Nature Limited.

Due to the low efficiency of the ultrasonic exfoliation method, it is extremely challenging to prepare TpBD COF nanosheets in a large quantity in water. However, macroscopic suspended solids were invisible after dissolving FeCl_3_ and ultrasonication for 1 h.^175^ Then, the TpBD COF nanosheets with a hydrodynamic diameter of ∼50 nm and thickness of 2.5 nm were successfully obtained with the removal of Fe^3+^ by dialysis. Quantitative calculations confirmed that Fe^3+^ could coordinate with the β-ketoenamine linkage of TpBD COF, resulting in an increase in the interlayer distance from 3.42 to 9.85 Å; further, the interlayer interaction energy changed from −362 to −19 kJ mol^−1^ (Fig. 20A). This increased interlayer spacing and weakened interlayer interaction energy lead to the facile insertion of solvent molecules between the COF layers and subsequent delamination. Similarly, the axial coordination of 4-ethylpyridine with the central metal ion of porphyrin can also coercively increase the spacing between the COF layers (Fig. 20B), thereby inducing the exfoliation of porphyrin-based COFs.^176^ The coordination reaction provides newer possibilities for the chemical exfoliation of COFs.

**Fig. 21:**
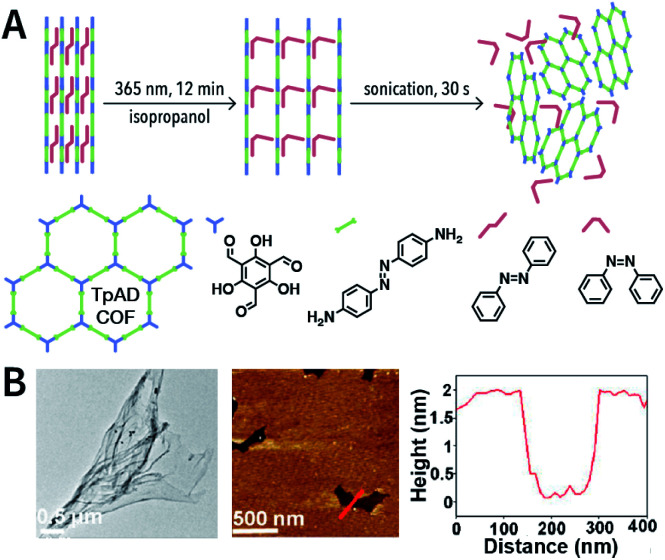
Azobenzene-assisted chemical exfoliation of TpAD COF into few-layer nanosheets. (A) Illustration of the azobenzene-assisted exfoliation method. (B) HRTEM and AFM images of TpAD COF nanosheets. Adapted with permission.^177^ Copyright 2020, Elsevier B.V.

Photochemical reactions can also induce the chemical exfoliation of COFs. As shown in Fig. 21, when TpAD COF loaded with *cis*-azobenzene guest molecules was exposed to ultraviolet light at 365 nm for 12 min in isopropanol, *cis*-azobenzene underwent the isomerization reaction. The formation of *trans*-azobenzene induced an increase in the layer spacing in TpAD COF and weakened the interlayer interaction. Remarkably, TpAD COF nanosheets with a thickness of 1.8 nm—equivalent to 6 layers of TpAD COF single layers—were obtained after only 30 s of ultrasonic treatment,^177^ while only 7% of the azo units in TpAD COF were converted into *trans*-isomers after 40 min of ultraviolet-light irradiation at 365 nm, which implied that only the guest molecules underwent isomerization and the internal structure of TpAD COF did not change.

The hydrolysis reaction of *n*-butyllithium has also been used for the chemical stripping of TpPa-1 COF.^178^ In an *n*-hexane solution, *n*-butyllithium was embedded into the TpPa-1 COF layers. After the hydrolysis reaction, TpPa-1 COF nanosheets were obtained with an astonishing productive rate of 80%.

**Fig. 22:**
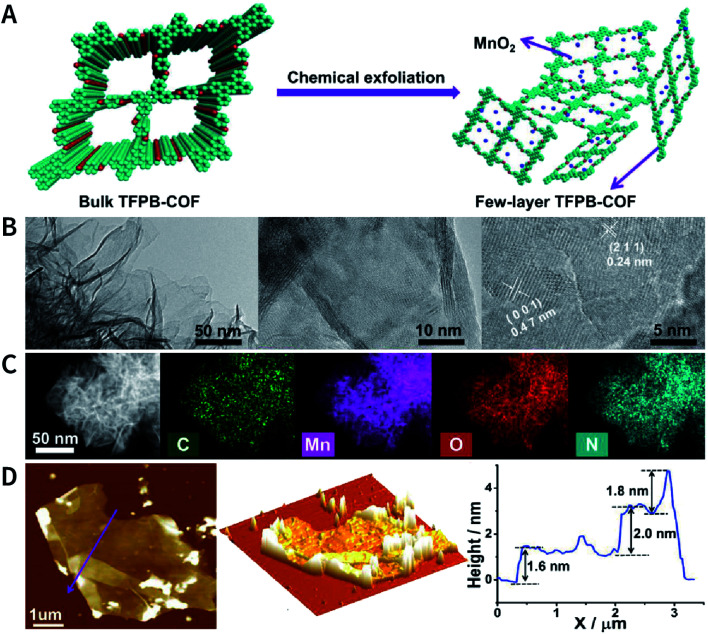
Reduction reaction of KMnO_4_ inducing the chemical exfoliation of TFPB-COF. (A) Schematic illustration for the chemical exfoliation of TFPB-COF. (B) HRTEM images of E-TFPB-COF/MnO_2_ nanosheets. (C) HAADF-STEM elemental mapping images of E-TFPB-COF/MnO_2_ nanosheets. (D) AFM images and measured thickness of E-TFPB-COF/MnO_2_ nanosheets. Adapted with permission.^179^ Copyright 2019, Wiley-VCH Verlag GmbH & Co. KGaA, Weinheim.

Another interesting method is to generate nanoparticles *in situ* between the COF layers *via* redox reactions, thereby realizing chemical exfoliation. As shown in Fig. 22, Wang *et al.* synthesized E-TFPB-COF nanosheets by the reduction reaction of KMnO_4_.^179^ Typically, bulk TFPB-COF and perchloric acid were mixed in water; then, KMnO_4_ was carefully added into the solution, which was maintained at 30 °C for 30 min. Thereafter, the solution was sonicated for 2 h to afford a black powder of E-TFPB-COF/MnO_2_. During the exfoliation process, MnO_2_ nanoparticles acted as spacers to effectively prevent the reaggregation of E-TFPB-COF. Finally, light-yellow E-TFPB-COF nanosheets could be obtained after etching away the MnO_2_ nanoparticles with hydrochloric acid. The thickness of the E-TFPB-COF nanosheets, as measured by AFM, was ∼1.6–2.0 nm.

#### 4.1.4 Gas-driven exfoliation

The key point of gas-driven exfoliation is to trigger the lattice expansion of a 2D material at high temperatures and then induce exfoliation by the vaporization of liquid nitrogen. After several “expansion–vaporization” cycles, the 2D material can be exfoliated into several atomic layers. This method has been used for the exfoliation of graphene,^180^ hexagonal boron nitride,^181^ and MOFs.^182^

**Fig. 23:**
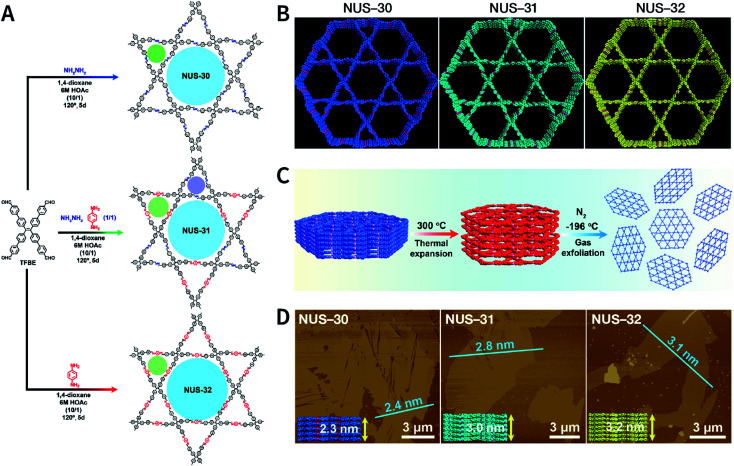
(A) Solvothermal syntheses of NUS-30, NUS-31, and NUS-32 COFs. (B) View of the slipped AA-stacking crystal structures of NUS-30, NUS-31, and NUS-32 COFs. (C) Temperature-swing gas exfoliation of NUS-30 from bulk powder to ultrathin nanosheets. (D) AFM images of NUS-30, NUS-31, and NUS-32 nanosheets. Inset: the theoretical thickness of NUS-30 (5 layers, 2.3 nm), NUS-31 (6 layers, 3.0 nm), and NUS-32 (7 layers, 3.2 nm) based on the AA-stacking structures. Adapted with permission.^183^ Copyright 2019, American Chemical Society.

Recently, three COFs (NUS-30, NUS-31, and NUS-32) with triangular and hexagonal pores were exfoliated by using this method (Fig. 23A and B).^183^ COF bulk powders were heated to 300 °C in air and maintained for 10 min, followed by immediately immersing them in liquid nitrogen (Fig. 23C); the above steps were repeated five times. Thereafter, the as-prepared COF nanosheets were centrifuged in acetonitrile to remove any large particles and to obtain COF nanosheets with thicknesses ranging from 2.4 to 3.1 nm (Fig. 23D). Although a few COF nanosheets have been successfully exfoliated *via* this method, its general applicability needs to be further explored. Exfoliation induced by gases instead of liquid nitrogen can be a promising alternative in the near future.

#### 4.1.5 Charge-mediated self-exfoliation

Due to electrostatic repulsion, the interactions between polymer chains with an embedded ionic characteristic decrease. Along this line, providing no or less external energy, COFs with positive charges on the framework may get spontaneously exfoliated, which is called charge-mediated self-exfoliation. Generally, the charge is located on the monomers, such as benzimidazolium,^184^ guanidinium,^185,186^ and viologen.^187–189^

**Fig. 24:**
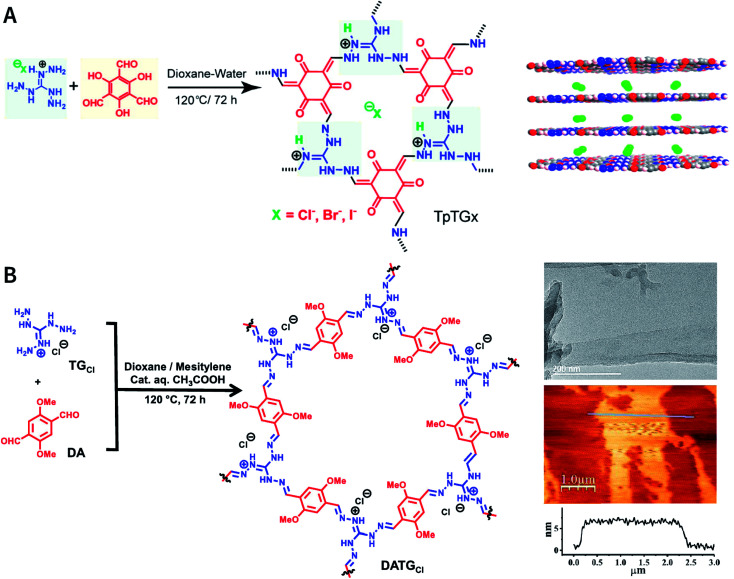
Self-exfoliated guanidinium-based COFs. (A) TpTG_X_ (X = Cl, Br, and I) COF based on triaminoguanidinium halides. Adapted with permission.^185^ Copyright 2016, American Chemical Society. (B) DATG_Cl_ COF nanosheets and their microtopography based on HRTEM and AFM imaging. Adapted with permission.^186^ Copyright 2020, American Chemical Society.

As reported by Banerjee *et al.*,^185^ three COFs, namely, TpTG_X_ (X = Cl, Br, and I), were constructed based on the Schiff-base condensation reaction of triaminoguanidinium halide and 2,4,6-trihydroxybenzene-1,3,5-tricarbaldehyde (Fig. 24A). Similar to hydrogen bonds, N–H⋯X interactions exist between the halide ions and guanidinium nitrogen of COFs, thereby immobilizing halide ions between the COF layers. The presence of interlamellar halide ions and inherent positive charges within the guanidine units causes repulsion and also interferes with the π–π-stacking interactions between the COF layers, leading to self-exfoliation into COF nanosheets with a thickness of only a few nanometers. As expected, TpTG_X_ spontaneously exfoliates in water, thereby affording TpTG_X_ nanosheets with a thickness of ∼2–5 nm. Due to the interaction of the positively charged nanosheets and negatively charged phospholipid bilayer of bacteria, the resulting TpTG_X_ nanosheets exhibited certain antibacterial activity. Recently, Pal *et al.* prepared highly fluorescent self-exfoliable DATG_Cl_ COF *via* a similar structural design.^186^ The thickness of the exfoliated DATG_Cl_ COF nanosheets was in the range of ∼5–7 nm, indicating that DATG_Cl_ COF nanosheets had ∼13–18 layers (Fig. 24B).

**Fig. 25:**
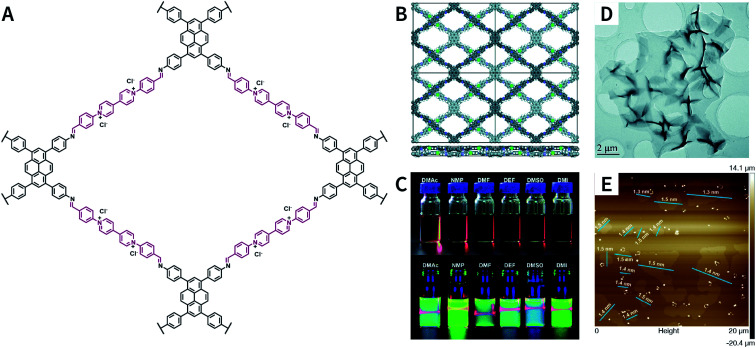
Self-exfoliated 4,4′-bipyridinium-based PyVg-COF. (A) Structure of PyVg-COF. (B) PyVg-COF with staggered AB-stacking arrangement. (C) Photographs of PyVg-COF nanosheets dispersed in various solvents under sunlight and 365 nm light. (D) TEM image of PyVg-COF nanosheets. (E) AFM image of PyVg-COF nanosheets. Adapted with permission.^188^ Copyright 2019, The Royal Society of Chemistry.

Pyridinium is another type of structural unit that constitutes self-exfoliated COFs. As shown in Fig. 25, using *N*,*N*-dimethylacetamide/mesitylene/6 M acetic acid (1 : 9 : 1, v/v/v) as the solvent, PyVg-COF with a staggered AB-stacking arrangement was prepared by the solvothermal reaction of 4,4′,4′′,4′′′-(pyrene-1,3,6,8-tetrayl)tetraaniline and 1,1-bis(4-formylphenyl)-4,4′-bipyridinium dichloride.^188^ The bipyridinium structural unit with high-density electrostatic repulsion was encoded into the framework to withstand interlayer π–π stacking, resulting in stronger interlayer interaction of PyVg-COF than that of the skeleton–solvent interaction. Therefore, PyVg-COF could be dispersed in a variety of organic solvents (*e.g.*, *N*-methyl pyrrolidone, dimethyl sulfoxide, *N*,*N*-dimethylformamide, *N*,*N*-diethylformamide, *N*,*N*-dimethylacetamide, and 1,3-dimethyl-2-imidazolidinone) by simple shaking. The critical aggregation concentration (CAC) of PyVg-COF in DMSO-*d*_6_ was up to 30 μg mL^−1^, below and above which monolayers and multilayers were respectively formed. Because of its highly charged skeleton and desirable dispersibility, ionic COF membranes could be easily prepared by means of the electrophoretic deposition method.

**Fig. 26:**
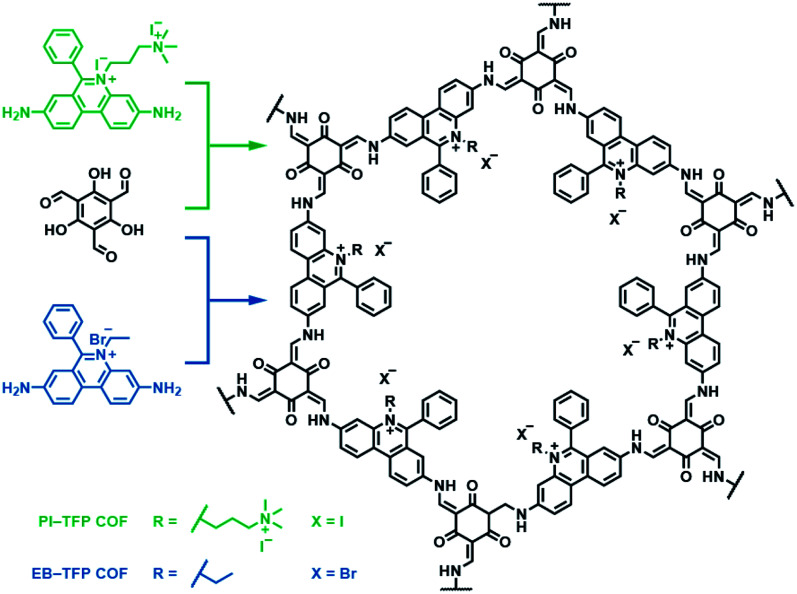
Self-exfoliated 6-phenylphenanthridin-5-ium-based COFs, PI-TFP COF, and EB-TFP COF, showing the supramolecular reassembly property.

Besides bipyridinium,^188,189^ phenanthridinium, such as ethidium and propidium, was also introduced into the frameworks to fabricate self-exfoliated ion-containing COFs. As shown in Fig. 26, the self-exfoliated EB-TFP COF^190^ and PI-TFP COF^191^ obtained in water showed average layer thickness distributions of 1.6 and 1.5 nm, respectively. Surprisingly, the supramolecular reassembly phenomenon exists in both COF nanosheets. For EB-TFP COF nanosheets,^190^ double-stranded DNA (dsDNA) induced their reaggregation, which created a hydrophobic environment over the ethidium unit and prevented the excited-state proton transfer process, thereby enhancing fluorescence emission. This phenomenon provides a unique opportunity to distinguish between dsDNA and single-stranded DNA (ssDNA). For PI-TFP COF,^191^ the host–guest interactions between PI-TPF COF and cucurbit[7]uril (CB[7]) lead to the restacking of nanosheets. After adding 1-adamantylamine hydrochloride, the nanosheets get self-exfoliated again. This reversible and controllable exfoliation implies the contribution of quaternary ammonium salt in propidium iodide to charge-mediate the self-exfoliation process.

Self-exfoliation induced by the charges within the frameworks is also observed in other types of COFs, such as iCOF-A-containing 1-methylpiperazine branched chain^192^ and COF_BTC_-containing iron phthalocyanine.^193^

**Fig. 27:**
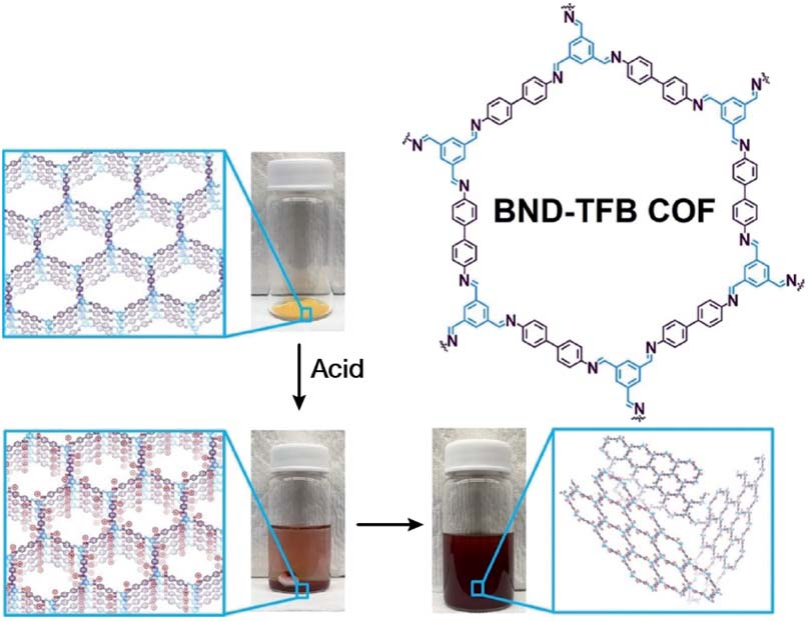
Charge-mediated self-exfoliation of BND-TFB COF *via* trifluoroacetic acid treatment. Adapted with permission.^194^ Copyright 2020, Wiley-VCH Verlag GmbH & Co. KGaA, Weinheim.

Self-exfoliation induced by the charges on the COF linkages is relatively scarce. In 2020, Dichtel *et al.* confirmed that the protonation of the C=N bond caused by acid treatment can induce the self-exfoliation of imine-linked COFs.^194^ BND-TFB COF (Fig. 27) with imine linkages was stirred in a mixture of acetonitrile/tetrahydrofuran/trifluoroacetic acid (7 : 3 : 2, v/v/v) overnight, which was delaminated into a suspension. After acid treatment, the protonated COF layer was positively charged and the charge repulsion induced their exfoliation. AFM and HRTEM images of BND-TFB COF nanosheets showed that the thickness was ∼5–50 nm and the diameter was ∼50–1000 nm. Moreover, two additional imine-linked COFs, namely, TAPB-PDA COF and methyl COF, were also exfoliated by acid treatment. Nanopipette-based electrochemical tests by Wang *et al.* confirmed that as the pH value decreased, the polarization of C=N bonds, slippage of layers, and exfoliation of COF occurred in sequence, finally leading to the formation of uniform COF nanosheets.^195^ Acid concentration and treatment time should be precisely controlled to achieve a balance between the degradation caused by the fracture of the covalent bond and exfoliation induced by the destruction of interlayer interaction.

### 4.2 Bottom-up synthesis

Although many strategies have been developed to prepare NCOFs by the top-down synthesis, these exfoliation techniques usually yield 2D nanosheets. The 2D nanosheet is very thin (<10 nm), but its size in the other two dimensions is still too large (up to several microns), which cannot meet the needs of biomedical applications. Therefore, it is imperative to develop an effective approach for the bottom-up synthesis of COF nanoparticles.

**Fig. 28:**
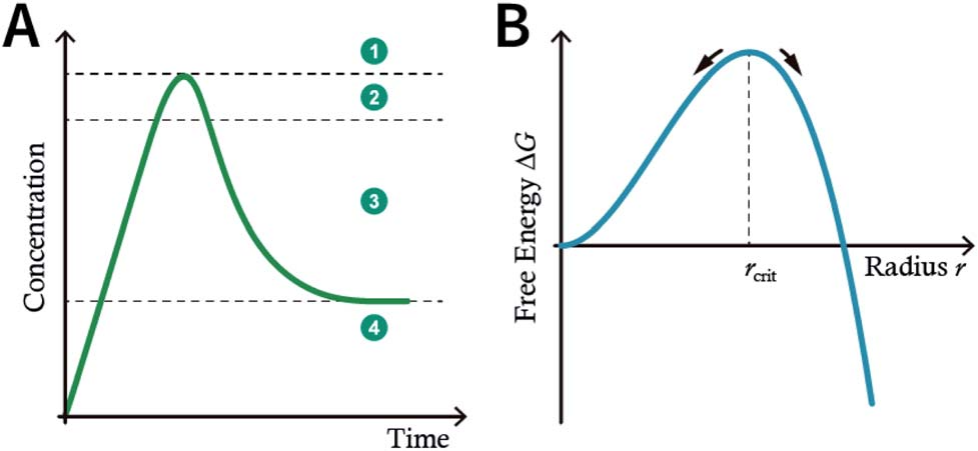
(A) Nanocrystal nucleation theory and growth model. (1) Critical limiting supersaturation; (2) rapid nucleation; (3) growth by diffusion; (4) solubility. (B) Radius corresponding to the maximum free energy is defined as the critical radius, which is the minimum size of particles that can survive in the solution without getting redissolved.

The nucleation and growth theories of nanocrystals are extremely complicated.^196,197^ In short, the synthesis of nanocrystals in a solution involves two important processes: nucleation followed by nanocrystal growth. In the early stages of the reaction, a rapid polymerization reaction occurs in the solution, affording polymer fragments with lower solubility. As the reaction proceeds, the polymer in the solution reaches supersaturation, breaking through the critical value required for nucleation. The nucleation stage is completed with precipitate formation. After that, the solution has a lower degree of supersaturation again, and the monomer continues to polymerize on the surface of the formed crystal nucleus with particles growing and becoming larger followed by a decrease in the monomer concentration in the solution. Finally, due to the reversibility of the COF linkages, the precipitation undergoes a dissolution–reprecipitation process, and the monomers tend to be arranged in a periodic order to form crystalline COF particles (Fig. 28A). Therefore, according to this model, to prepare uniform-sized nanoparticles, a large amount of nucleation should be explosively formed in the shortest possible time such that the above process is separated as much as possible. The simultaneous nucleation and growth processes may result in the formation of particles with nonuniform sizes.

In the nucleation stage, according to the theory of crystal nucleation, for spherical crystal nuclei,
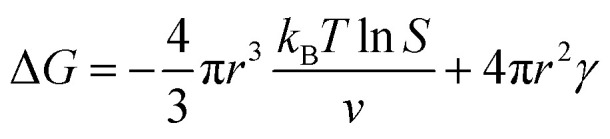
where Δ*G* represents the total free energy change when a new phase is formed, *v* corresponds to the molar volume of the crystal, *r* is the radius of the nucleus, *k*_B_ represents the Boltzmann constant, *S* is the solution supersaturation, and *γ* represents the surface free energy per surface area. The functional relationship between Δ*G* and *r* is shown in Fig. 28B.

When *S* > 1, the maximum value of Δ*G* is obtained at
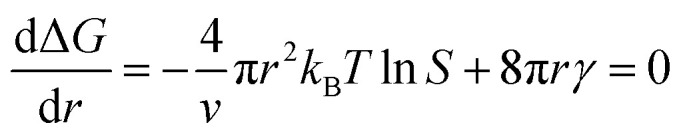
therefore,1
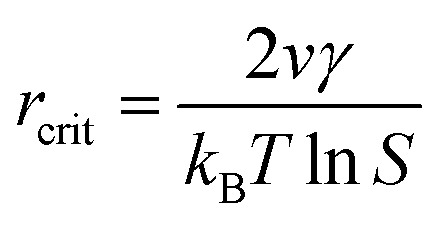


This critical radius *r*_crit_ corresponds to the minimum size at which a particle can survive in solution without being redissolved (Fig. 28B). Therefore, it is possible to reduce *r*_crit_ by increasing *S* or decreasing *γ* to promote nucleation. Besides, rate control is also significant during growth, and ripening in addition to avoiding secondary nucleation can improve the uniformity of nanoparticles and increase in crystallinity.

According to the aforementioned nanocrystal nucleation theory and growth model, we will comprehensively introduce and discuss the bottom-up synthesis strategies of NCOFs and summarize the related examples in the following section.

#### 4.2.1 Reaction kinetics regulation

Here, we will illustrate the applications of reaction kinetics regulation (*e.g.*, changing the reaction time, controlling the rate of monomer addition, and adding nucleation modulators) for the preparation of NCOFs.

**Fig. 29:**
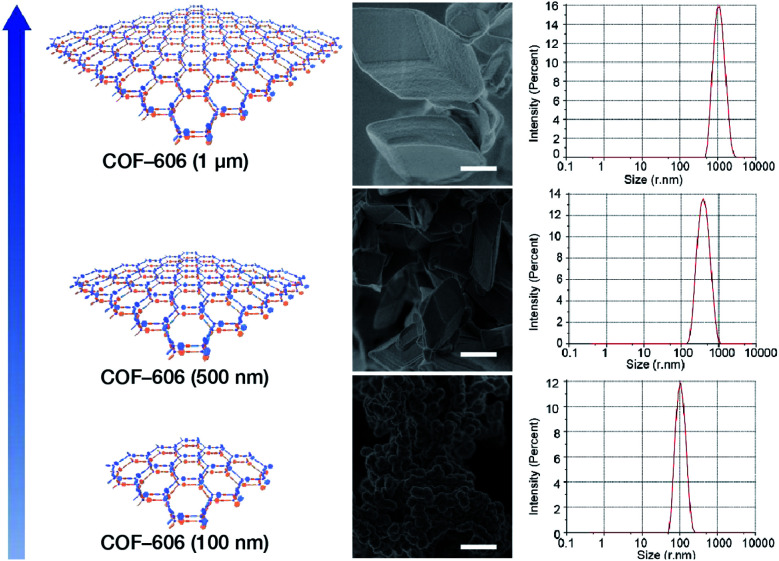
COF-606 with different particle sizes measured by SEM and DLS. Scale bar: 500 nm. Adapted with permission.^198^ Copyright 2020, Elsevier Inc.

Deng *et al.* prepared COF-606 with different particle sizes (Fig. 29) by varying the temperature and reaction time of the solvothermal reaction.^198^ In order to prepare COF-606 with particle sizes of 500 nm and 1 μm, a Pyrex tube containing the building blocks and solvents was placed in an oven and heated at a programmed temperature at a constant rate of 0.1 to 90 °C and maintained for 7 days. When the precipitate was immediately separated, the particle size of the obtained COF-606 was 500 nm. If the Pyrex tube was cooled down to room temperature under programmed temperature at a constant rate of 0.1 °C, the particle size of the obtained COF-606 was 1 μm. More importantly, if the Pyrex tube was directly heated at 65 °C for 12 h, the particle size of COF-606 was reduced to 100 nm. Indeed, this synthesis strategy is fully consistent with the theory of nanocrystal nucleation and growth. A slow increase in temperature inhibits the nucleation process, and a slow decrease in temperature promotes the growth process such that large-sized particles can be obtained. The rapid and short-term reaction favors explosive nucleation, retards growth, and tends to form small-sized COF nanoparticles.

The feasibility of the preparation of NCOFs by shortening the reaction time was further verified by TTA-AzoDFP NCOF synthesis.^199^ TTA-AzoDFP NCOF was synthesized by the condensation of 4,4′,4′′-(1,3,5-triazine-2,4,6-triyl)trianiline (TTA) with (*E*)-4-(4-(phenyldiazenyl)phenyl)pyridine-2,6-dicarbaldehyde (AzoDFP) in 1,4-dioxane in the presence of acetic acid (3 M) under solvothermal conditions at 120 °C for 1 h. TTA-AzoDFP NCOF is a spherical nanoparticle with a rough surface and the diameter of the particle is only 117 nm. The shorter reaction time (1 h) inhibits the growth process of nanocrystals, resulting in small-sized particles.

**Fig. 30:**
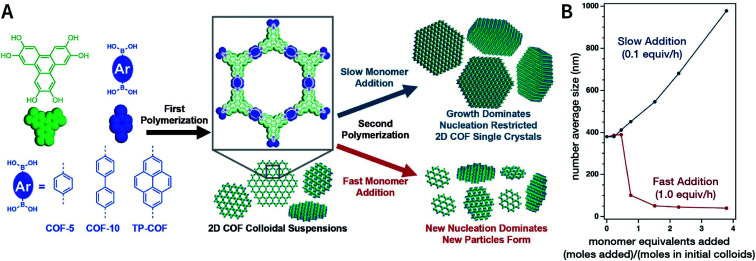
Seeded growth for the synthesis of boronate-ester-linked COFs with different particle sizes. (A) Effect of the rate of adding monomers on the particle size. (B) Average size of COF-5 particles as a function of the added monomer equivalents. Adapted with permission.^200^ Copyright 2018, The Author(s). Published by American Association for the Advancement of Science.

In 2018, Dichtel *et al.* prepared COF-5 with different particle sizes by adding different concentrations of reactants at different rates to a pre-prepared COF-5 seed crystal with a particle size of 400 nm.^200^ When the monomer was gradually added to the reaction mixture, the monomer concentration became limited. Growth dominated nucleation; therefore, the particles gradually grew without forming newer particles, which resulted in an increased particle size. In contrast, when monomers were rapidly added, their concentration increased above the critical nucleation concentration. The reaction was dominated by the formation of new and small-sized particles (Fig. 30). Furthermore, the quantitative analysis of COF-5 nucleation and growth by kinetic Monte Carlo (KMC) simulations^201,202^ showed that there was a threshold of the monomer concentration below which growth dominated nucleation and nucleation and growth rates had second- and first-order dependence on the monomer concentration, respectively. Besides COF-5, studies on COF-10 and TP-COF also confirmed the above trends.^200^

Unfortunately, unlike amorphous materials and easily crystallized inorganic materials, the crystallization of COFs is a crucial process that accompanies nucleation and growth processes. For COF growth, particularly boronate-ester-linked COF growth, the decisive step is not condensation, but interlayer polymer stacking by a nucleation–extension process.^203^ Therefore, indiscriminately promoting nucleation and inhibiting growth may reduce the crystallinity of COFs and even lead to the formation of amorphous polymers. In this context, a reasonable balance between nucleation and growth is imperative. One effective strategy is to add monofunctional species (*e.g.*, phenylboronic acid, catechol, benzaldehyde, and phenylamine) as the modulators, which can affect the reaction rate and thermodynamic equilibrium state by participating in the polymerization reaction. This strategy may be suitable for the synthesis of COF particles with any size. For instance, Bein *et al.* reported that the addition of (4-mercaptophenyl)boronic acid modulator in COF-5 syntheses increased their crystallinity by slowing down the COF-5 growth and supporting the self-healing of crystal defects.^204^ Dichtel *et al.* also demonstrated a similar effect for pyrocatechol in COF-5 synthesis.^205^ Recently, modulators have been used to maintain a thermodynamically stable state to regulate the morphology of COFs, such as COF spheres,^206^ hollow fibers,^206^ single crystals,^207,208^ and thin films.^206,209^ Additionally, when the added competitor has a chiral site (*e.g.*, (*S*)-1-phenylethan-1-amine), this strategy can possible generate chiral COFs,^210^ even though the monomers used for the construction of COFs do not exhibit any chirality.

**Fig. 31:**
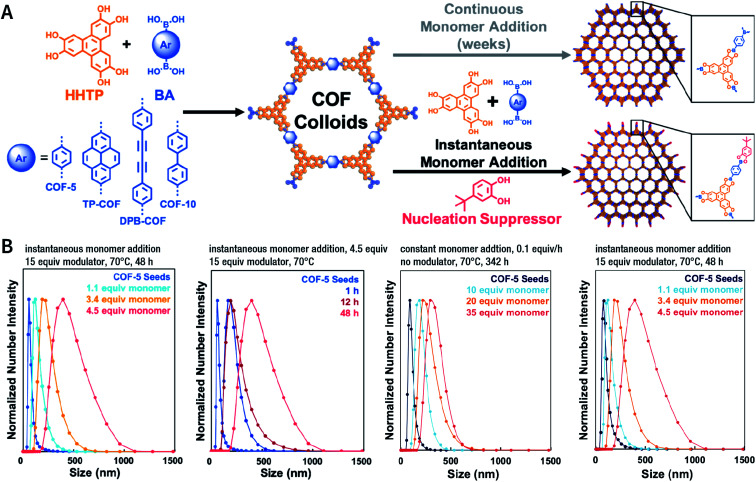
Chemical control over nucleation and anisotropic growth of COFs. (A) COF colloidal seeds formed by monomers can be grown *via* two patterns. One is slowly adding monomers, resulting in slower growth. The other is instantaneously adding the competitor and monomers. (B) Average size distribution of COF-5 particles under different synthesis conditions. Adapted with permission.^211^ Copyright 2019, American Chemical Society.

Among them, Dichtel *et al.* demonstrated that the addition of high-dosage 4-(*tert*-butyl)benzene-1,2-diol to the synthesis reaction of COF-5 inhibited nucleation in a concentration-dependent mode and induced subsequent anisotropic growth.^211^ When compared with the constant addition of monomers, the competitor significantly shortened the synthesis time without reducing the crystallinity. By controlling the reagent concentration and reaction time, the hydrodynamic diameter of COF-5 particles was adjustable within the range of ∼60–450 nm (Fig. 31). By adding the competitor in a similar way, three other boronate-ester-linked COF materials, namely, TP-COF, DPB-COF, and COF-10, were also obtained within a hydrodynamic diameter range of ∼110–1400, ∼90–260, and ∼110–800 nm, respectively.

#### 4.2.2 Surfactant-assisted synthesis

According to the earlier discussion, in the eqn (1), reducing *γ* can reduce *r*_crit_, thereby affording small-sized nuclei. To our delight, surfactants can reduce *γ* by reducing the tension at the solid–liquid interface.^212,213^ Therefore, surfactant-assisted synthesis is one of the most effective methods for synthesizing NCOFs.

**Fig. 32:**
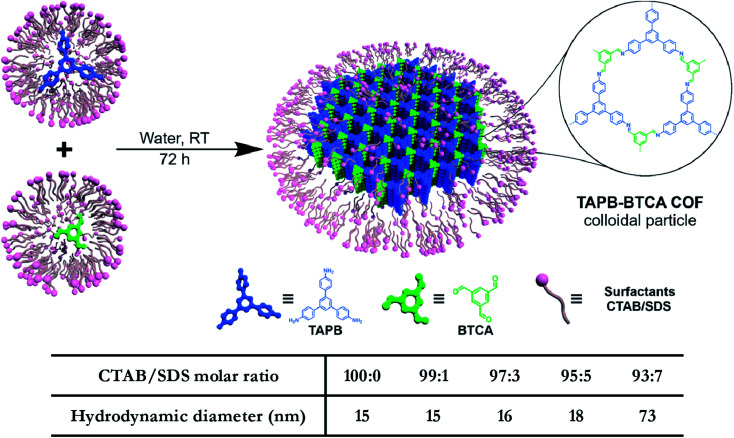
Catanionic-surfactant-assisted synthesis of TAPB-BTCA COF nanoparticles in water. By varying the ratio of the surfactants, the size of the nanocrystals can be controlled to sub-20 nm. Adapted with permission.^214^ Copyright 2020, American Chemical Society.

In addition to regulating the nucleation and growth processes of COFs, the solubilizing effect of surfactants on organic monomers enables aqueous synthesis. Puigmartí-Luis *et al.* synthesized imine-linked TAPB-BTCA COF nanoparticles in water using a surfactant mixture (Fig. 32). Due to the formation of micelles, the water-insoluble benzene-1,3,5-tricarbaldehyde and 1,3,5-tris(4-aminophenyl)benzene monomers were effectively dissolved in aqueous solutions containing cationic hexadecyltrimethylammonium bromide (CTAB) and anionic sodium dodecyl sulfate (SDS) surfactants, thereby forming two homogeneous solutions. When acetic acid was added to the mixture, the mixture turned orange, indicating the formation of the C=N bond. An aqueous colloidal solution of TAPB-BTCA COF was obtained after reaction for 72 h at 30 °C. The particle size of TAPB-BTCA COF strongly depended on the ratio of CTAB to SDS; by increasing the amount of SDS, the hydrodynamic diameter of TAPB-BTCA COF could range from 15 to 73 nm. Additionally, another imide-based COF, *i.e.*, Tz-COF, with a particle size of about 20 nm, could also be prepared *via* the reaction of 2,4,6-tris(4-aminophenyl)-1,3,5-triazine and benzene-1,3,5-tricarbaldehyde in CTAB/SDS (97 : 3) mixtures, demonstrating the feasibility of this method.

**Fig. 33:**
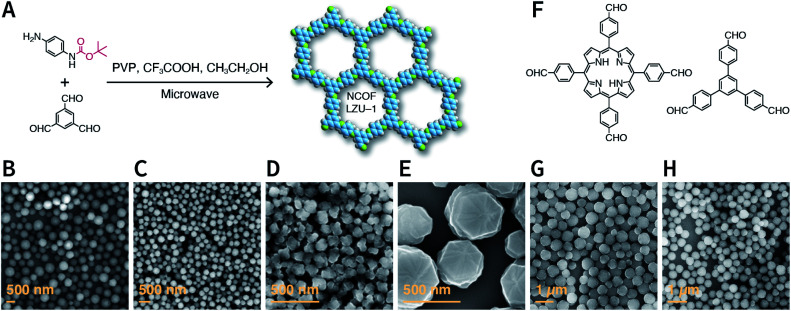
Synthesis of NCOFs using *tert*-butyl (4-aminophenyl)carbamate as the precursor. (A) Synthesis of NCOF LZU-1. (B–D) LZU-1 nanocrystals with different particle sizes. (E) Hexagonal LZU-1 nanocrystals. (F) Aldehydes for the synthesis of nanoscale Por-COF and TFPB-PDA-COF. (G) Por-COF nanocrystals. (H) TFPB-PDA-COF nanocrystals. Adapted with permission.^215^ Copyright 2017, American Chemical Society.

Rapid heating and slow growth are beneficial to COF nanocrystallization. For this purpose, two strategies have been developed for surfactant-assisted COF nanocrystal synthesis. First, microwaves provide speedy and uniform heating, which facilitates a nucleation burst. Second, the amine monomer is protected with *tert*-butoxycarbonyl, which is deprotected *in situ* during the reaction, thereby delaying the growth of the crystal nuclei. As shown in Fig. 33A, under microwave treatment, *tert*-butyl-(4-aminophenyl)carbamate and benzene-1,3,5-tricarbaldehyde reacted in ethanol in the presence of polyvinylpyrrolidone (PVP) and trifluoroacetic acid, affording uniform LZU-1 nanocrystals.^215^ The protonation of the imine bond rendered the nanocrystals to become polar during the growth process, allowing PVP to bind and passivate its surface, thereby regulating the growth process. By changing the molecular weight and concentration of PVP, the particle size of LZU-1 can be tuned as 500 ± 52, 245 ± 25, and 112 ± 11 nm (Fig. 33B–D). When toluene was added to the reaction system to reduce the polarity of the solution, LZU-1 assumed a hexagonal shape (Fig. 33E). This method is also used for the synthesis of Por-COF and TFPB-PDA COF nanocrystals using 4,4′,4′′,4′′′-(porphyrin-5,10,15,20-tetrayl)tetrabenzaldehyde and 1,3,5-tris(4-formylphenyl)benzene as the monomers (Fig. 33F–H).

Along this line, in 2019, Dong *et al.* successfully synthesized NCOF LZU-1 under solvothermal conditions instead of the aforementioned microwave method.^216^ More importantly, the obtained LZU-1 NCOF exhibited a high surface area of 822 m^2^ g^−1^, which is twofold higher than that of the original report (410 m^2^ g^−1^).^64^

#### 4.2.3 Acetonitrile method

Acetonitrile—a common organic solvent—has been successfully used for the preparation of B–O- and C=N-linked NCOFs, showing extraordinary application potential in the bottom-up synthesis of NCOFs.

**Fig. 34:**
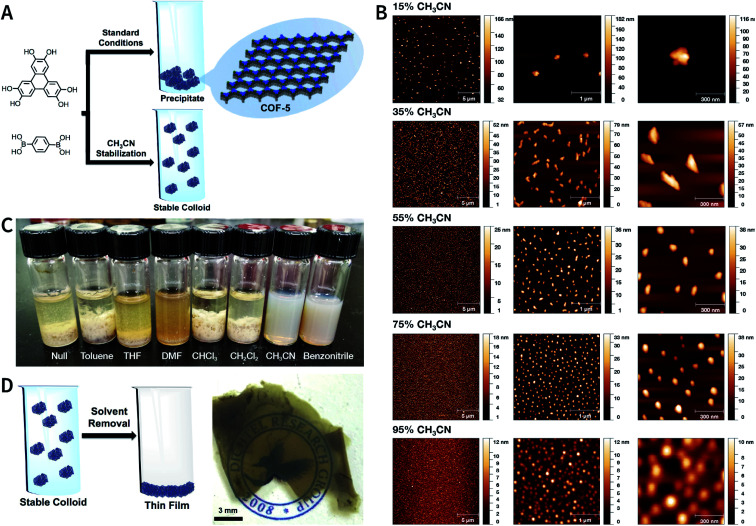
Boronate-ester-linked COF-5. (A) Synthesis of stable colloidal COF-5 nanoparticles using acetonitrile as the cosolvent. (B) AFM of COF-5 nanoparticles prepared at different volume ratios of acetonitrile and 1,4-dioxane/mesitylene (4 : 1, v/v). (C) COF-5 synthesized in additive solvent/1,4-dioxane/mesitylene (5 : 4 : 1, v/v/v). (D) Centimeter-scale COF-5 thin films fabricated *via* colloidal solution casting. Adapted with permission.^217^ Copyright 2017, American Chemical Society.

**Fig. 35:**
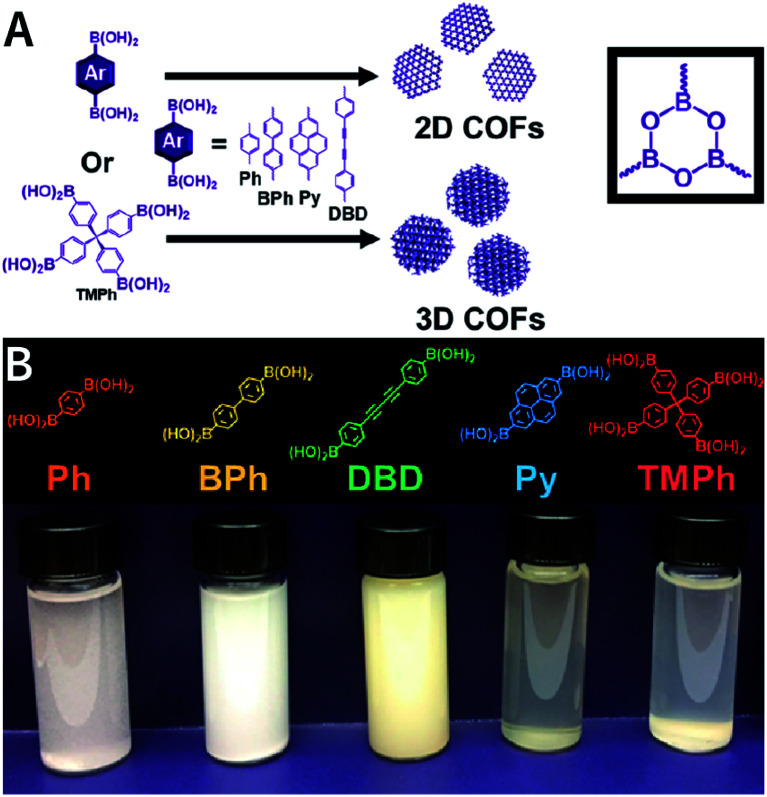
Synthesis of boroxine-linked 2D and 3D COFs in acetonitrile/1,4-dioxane/mesitylene (5 : 4 : 1, v/v/v). Adapted with permission.^218^ Copyright 2019, American Chemical Society.

As shown in Fig. 34, by the cocondensation of triphenylene-2,3,6,7,10,11-hexaol and 1,4-phenylenediboronic acid, Dichtel *et al.* synthesized translucent COF-5 dispersions in a ternary mixed solvent of acetonitrile, 1,4-dioxane, and mesitylene at 70 °C.^217^ When the acetonitrile content increases from 15 to 95 vol%, the hydrodynamic diameter of COF-5 drops from 232 to 50 nm. Acetonitrile is irreplaceable for colloidal stabilization, whereas other alternative solvents for acetonitrile (*e.g.*, toluene, tetrahydrofuran, *N*,*N*-dimethylformamide, chloroform, and methylene chloride) lead to COF-5 precipitation. The authors speculate that the direct interaction between the cyano group and COF-5 is responsible for colloid formation, although the details for this interaction are still not fairly clear. Moreover, as compared to the poor processability of microcrystalline powders, these stable COF colloidal suspensions provide a new avenue for processing these materials into centimeter-scale thin films from solution. Besides boronate-ester-linked COFs, boroxine-linked Ph-COF, BPh-COF, DBD-COF, Py-COF, and TMPh-COF colloidal dispersions (Fig. 35) were also synthesized in mixed solvents containing acetonitrile by the trimerization of diboronic acids.^218^

**Fig. 36:**
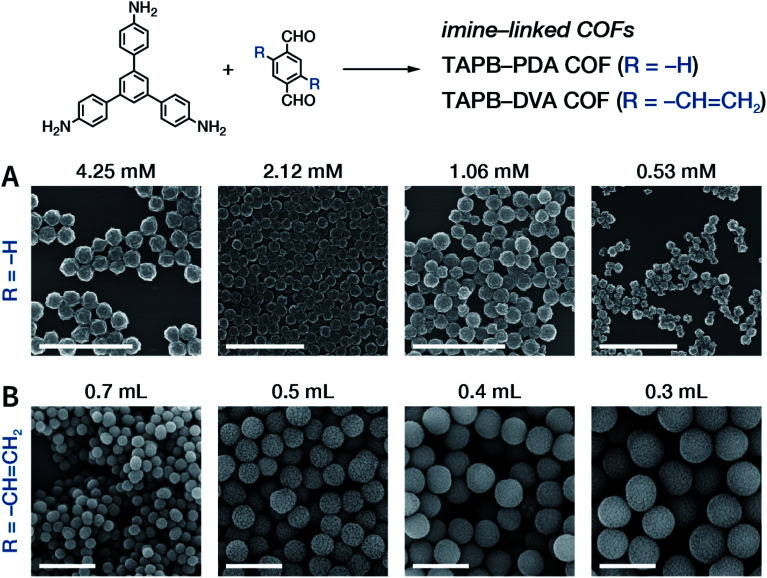
SEM images of imine-linked COFs prepared in CH_3_CN at room temperature. (A) TAPB-PDA COF with particle sizes of 200, 260, 290, and 330 nm prepared at varying aryltriamine monomer concentrations. Scale bar: 2 μm. Adapted with permission.^219^ Copyright 2019, The Royal Society of Chemistry. (B) TAPB-DVA COF with particle sizes of 450, 800, 1000, and 1300 nm synthesized at different volumes of acetic acid. Scale bar: 2 μm. Adapted with permission.^222^ Copyright 2019, American Chemical Society.

According to the study reported by Dichtel *et al.*, acetonitrile was also used as a solvent for the synthesis of imine-linked COFs.^219^ Briefly, scandium(iii) trifluoromethanesulfonate as a transamination catalyst^131^ was added to the acetonitrile solutions of 1,3,5-tris(4-aminophenyl)benzene and *p*-phthalaldehyde (Fig. 36). Then, the mixture was stirred for 20 h at room temperature. The particle size of the obtained TAPB-PDA COF varied from the minimum of 200 nm to the maximum of 500 nm with changes in the initial monomer concentration. This approach has been widely applied to the synthesis of various Schiff-base COFs,^220–224^ implying its widespread feasibility.

#### 4.2.4 Interfacial synthesis

**Fig. 37:**
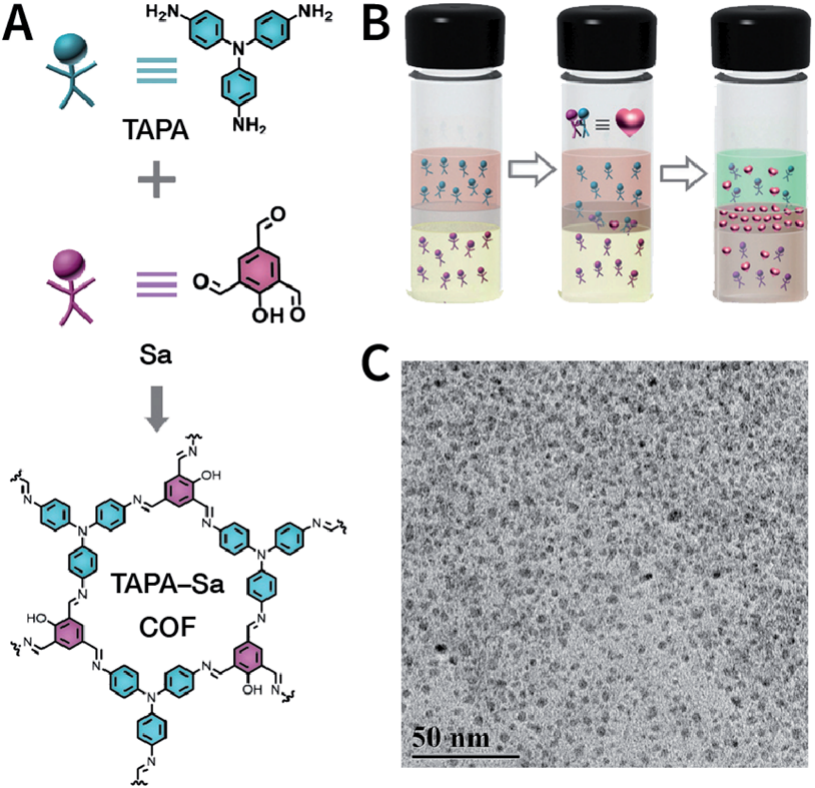
Crystalline TAPA-Sa COF quantum dots. (A) Chemical structure of TAPA-Sa COF. (B) Schematic of the interfacial growth process. (C) HRTEM image of TAPA-Sa COF quantum dots with an average size of 4.2 nm. Adapted with permission.^230^ Copyright 2020, The Royal Society of Chemistry.

Diffusion-based COF synthesis at the solid–liquid,^225^ liquid–liquid,^226–228^ or liquid–gas^229^ interfaces usually yields nanosheets or thin films. Although the thickness can be controlled to a few nanometers or a dozen nanometers, the length and width are usually in the range of several microns. An interesting exception is the interfacial synthesis of TAPA-Sa COF quantum dots (Fig. 37).^230^ The monomer of 2-hydroxybenzene-1,3,5-tricarbaldehyde was dissolved in dichloromethane/*N*,*N*-dimethylformamide (40 : 1, v/v) solvent; then, acetic acid (12 M) was slowly added. Finally, TAPA in *N*,*N*-dimethylformamide solution was carefully added dropwise to the acetic acid surface. As TAPA moved to the intermediate layer and got protonated, its color gradually changed from red to green. At the same time, 2-hydroxybenzene-1,3,5-tricarbaldehyde in the lower layer slowly diffused to the intermediate layer, thereby generating a large amount of COF quantum dots. When the reaction time was 3 days, the yield was about 29%, and the average particle size was 4.2 nm.

#### 4.2.5 Template method

The template method uses a readily synthesized nanostructure as the core; then, a shell material is deposited on its surface. After removing the inner core, hollow nanoparticles can be obtained. Since Möhwald *et al.* prepared hollow silica nanospheres using the template method in 1998,^231^ it has become one of the most efficient methods for nanomaterial synthesis. The material and structure of the template can be customized according to the property and morphological requirements of the target materials. For the materials that are difficult to reduce to the nanoscale, this method is facile, straightforward, and highly effective.

**Fig. 38:**
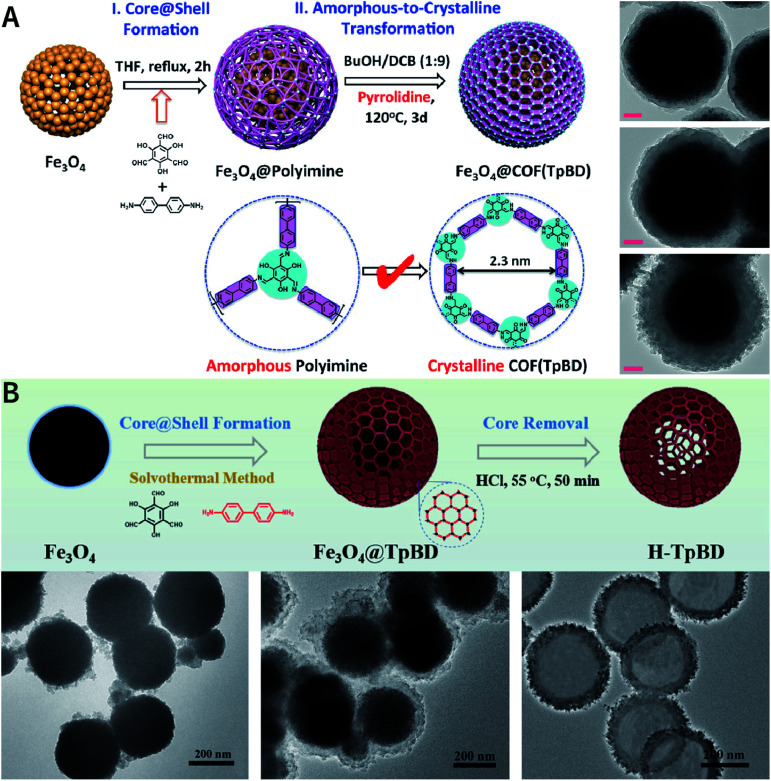
(A) Preparation protocol and HRTEM images of Fe_3_O_4_@COF (TpBD) with shell thicknesses of 20, 50, and 100 nm. Scale bar: 50 nm. Adapted with permission.^232^ Copyright 2016, Wiley-VCH Verlag GmbH & Co. KGaA, Weinheim. (B) Synthesis protocol and HRTEM images of hollow COF nanoshells. Adapted with permission.^233^ Copyright 2018, Elsevier B.V.

Fe_3_O_4_ was the first template used for the synthesis of NCOFs. Since Fe_3_O_4_ is usually prepared by the hydrothermal reaction of sodium citrate with iron salts, the surface of Fe_3_O_4_ nanoparticles normally adsorbs citrates, which further facilitates the adsorption of arylamine molecules *via* hydrogen bonds, resulting in polymerization reactions on the Fe_3_O_4_ surface. As shown in Fig. 38, Wang *et al.* employed this method to grow an amorphous polyimide network on the Fe_3_O_4_ surface, which was further converted into crystalline COFs *via* solvothermal synthesis.^232^ By varying the concentration of COF monomers, the thickness of the COF shell can be controlled. If further etching of Fe_3_O_4_ is undertaken with hydrochloric acid, hollow COF nanoshells can be obtained.^233^ In addition, considering the magnetic property of Fe_3_O_4_, core–shell Fe_3_O_4_@COFs have shown great promise for magnetic separation and enrichment.^234,235^

**Fig. 39:**
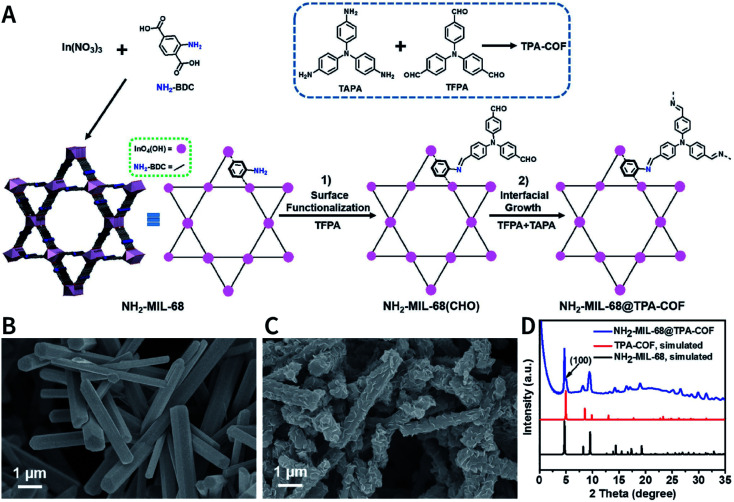
(A) Synthesis of NH_2_-MIL-68@TPA-COF hybrid material. (B) SEM image of NH_2_-MIL-68. (C) SEM image of NH_2_-MIL-68@TPA-COF. (D) SAXS/WAXS patterns. Adapted with permission.^236^ Copyright 2018, Wiley-VCH Verlag GmbH & Co. KGaA, Weinheim.

The pre-modification of monomers on the templates is more robust to provide COF growth sites *via* covalent bonds as compared to that *via* hydrogen bonds. Therefore, templates with amino groups on the surface may facilitate the controlled template synthesis of C=N-linked COFs. Zhang *et al.* were the first to pre-modify the amino group on the surface of indium-based MOF NH_2_-MIL-68 template with an aldehyde monomer *via* imine condensation and then extended the polymerization of TPA-COF using formyl groups on the surface as the reactive sites; finally, the NH_2_-MIL-68@TPA-COF core–shell structure was successfully prepared (Fig. 39).^236^ Other MOFs with amino groups, such as UiO-66-NH_2_,^237,238^ NH_2_-MIL-125(Ti),^239,240^ and NH_2_-MIL-101(Fe),^241^ can also be used as templates to anchor aldehyde monomers *via* covalent bonds.

**Fig. 40:**
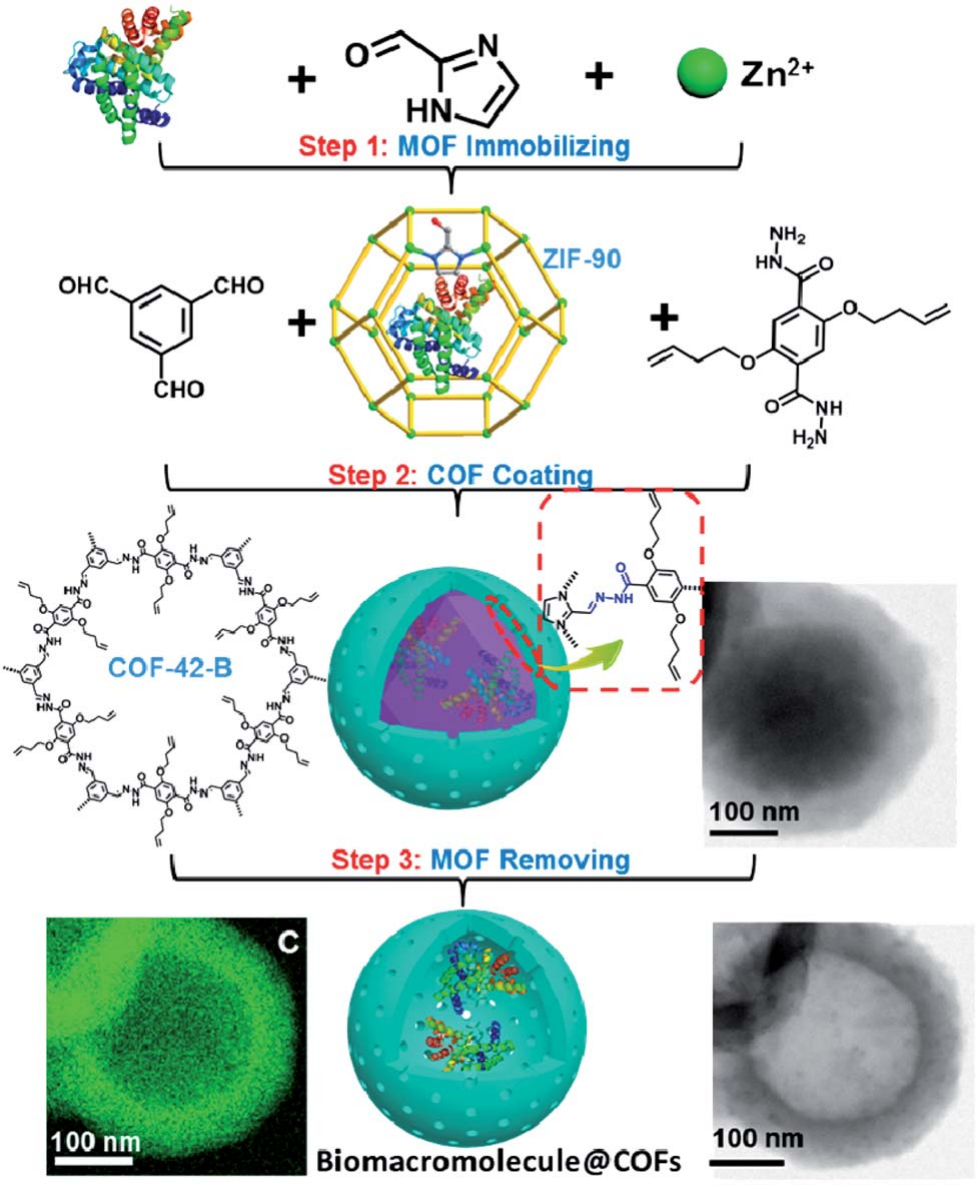
Using ZIF-90 as the sacrificial templates to construct hollow COF-42-B nanocapsules for biomacromolecule encapsulation. Adapted with permission.^242^ Copyright 2020, American Chemical Society.

Very recently, Chen *et al.* prepared hollow COF capsules using MOFs as the template to encapsulate biomacromolecules (Fig. 40), which could not be directly loaded onto solid COFs by simple adsorption.^242^ First, biomacromolecules were encapsulated into acid-labile MOFs by *in situ* biomimetic mineralization to yield a template for COF growth. Then, considering the fact that acetic acid readily leads to the premature dissolution of MOFs, the COF shells were grown at room temperature with Sc(OTf)_3_ as the transamination catalyst. Finally, the MOF core was etched away to afford hollow COF capsules loaded with biomacromolecules. The COF shells protect the biomacromolecules and prevent interference from the external environment. More importantly, its spacious interior maintains the conformational freedom of the biomacromolecules while allowing the efficient diffusion of substrates and products. The generalizability of this approach was confirmed by selecting different MOFs (*e.g.*, ZIF-90, ZIF-8, and ZPF-2), COFs (*e.g.*, COF-42-B and COF-43-B), and biomacromolecules (*e.g.*, bull serum albumin, catalase, and glucose oxidase). In addition, in CO_2_-dissolved water, hollow LZU-1 and COF-42 were synthesized using ZIF-8 and ZIF-67 as the templates.^243^

In addition to the aforementioned hard templates (*e.g.*, Fe_3_O_4_ and MOFs), soft templates, such as oil-in-water (O/W) microemulsions,^244^ can also be used to fabricate hollow NCOFs. Unlike the demanding etching conditions of hard templates, soft templates can be readily removed from the core–shell structures by evaporating or adding demulsifiers.

Finally, some of the representative examples of COF core–shell structures synthesized using different templates are summarized in Table 3.^94,233–261^

**Table 3:** Core–shell structures obtained by growing COFs on different nanotemplates

Core	Size of core	Shell	Thickness of shell	Ref.
Fe_3_O_4_	150 nm	Guanidyl-based COF	Tens of nanometers	94
Fe_3_O_4_	160 nm	Imine-linked COF	5 nm	245
Fe_3_O_4_	40 nm	LZU-1	Not mentioned	246
Fe_3_O_4_	409–614 nm	COF-DtTb	92, 120, and 177 nm	247
Fe_3_O_4_	336 nm	CTpBD	31 nm	248
Fe_3_O_4_	300 nm	TpBD COF	50 nm	233
Fe_3_O_4_	5 nm	mTpBD COF	Hundreds of nanometers	249
Fe_3_O_4_	340 nm	TAPB-DMTP-COF	150 nm	250
Fe_3_O_4_	250 nm	TAPB-PDA COF	25 nm	251
Fe_3_O_4_@SiO_2_	150–200 nm	TpBD(NO_2_)_2_	44 nm	252
MCNC	200–300 nm	TAPB-DVA COF	20 nm	253
SiO_2_	360 nm	BD-TPB COF	20–30 nm	254
SiO_2_	170 ± 12 nm	COF_TTA-DHTA_	32 ± 6 nm	255
SiO_2_, ZnS, and UiO-66	410, 390, and 490 nm	TAPB-DMTP-COF	Adjustable	256
UiO-66-NH_2_	150–200 nm	TFPT-DETH-COF	20 ± 1 nm	237
UiO-66-NH_2_	100 nm	Tp-PDA COF	10 nm	238
UiO-66-NH_2_	100 nm	Tp-TPE COF	10 nm	238
UiO-66-NH_2_	200 nm	LZU-1	10 nm	257
NH_2_-MIL-125(Ti)	Hundreds of nanometers	TAPB-PDA COF	10–60 nm	239
NH_2_-MIL-125(Ti)	Hundreds of nanometers	LZU-1	20 nm	240
NH_2_-MIL-101(Fe)	200 nm	NTU-COF	20–40 nm	241
NH_2_-MIL-68	Microrod	TPA-COF	50–200 nm	236
MIL-69	Microrod	TPA-COF	200–400 nm	236
PCN-222-Co	380 × 200 nm	TpPa-1	15 ± 3 nm	258
CNTs	Nanotube	2D-PAI	4–6 nm	259
CNTs	Nanotube	TpPa-COF	25 nm	260
PAA-UCNPs	30 nm	Porphyrin-based COF	15–30 nm	261

## 5 Functionalization of COFs

In the earlier section, we discussed methods for preparing NCOFs. However, to some extent, NCOFs are only platforms; additional functional active sites have to be integrated with them to realize some task-specific biomedical applications. The process of incorporating functional components is better known as COF functionalization. Therefore, in this section, we will systematically discuss the functionalization approaches involving COFs.

**Fig. 41:**
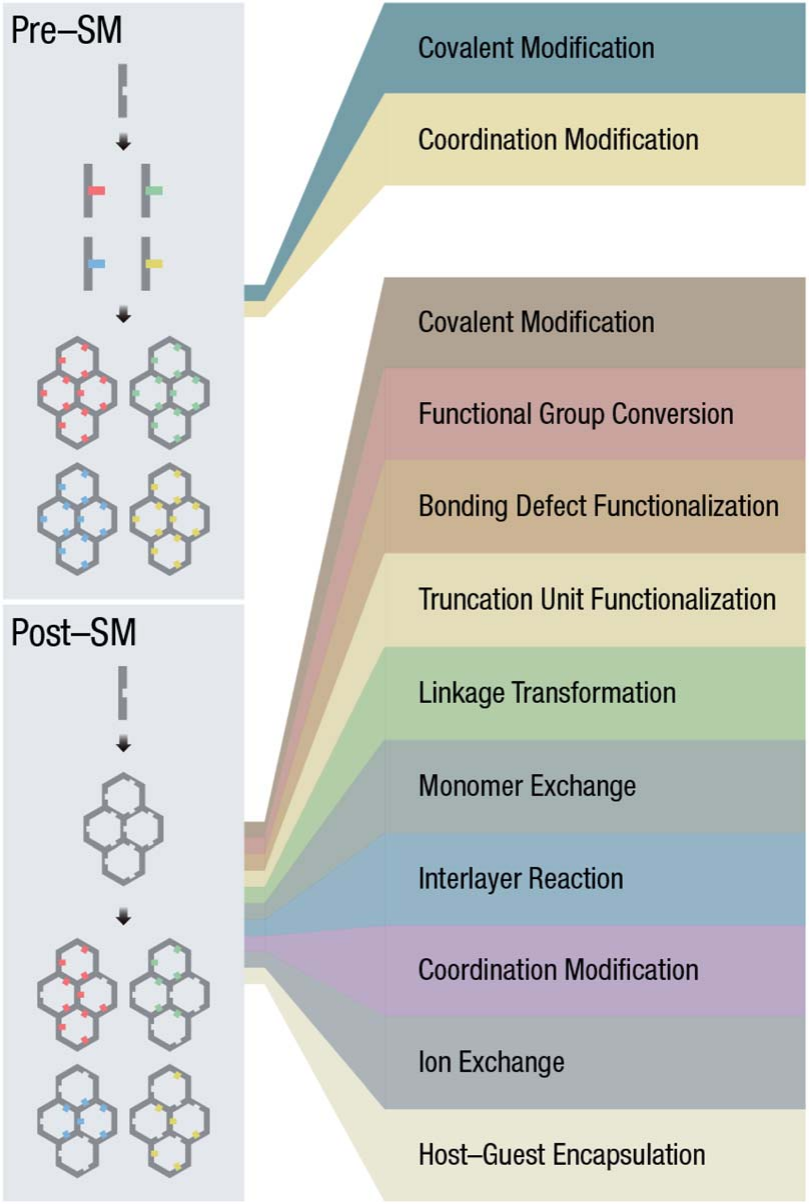
Functionalization of COFs.

Generally, approaches for COF functionalization (Fig. 41) can be divided into pre-synthesis modification (pre-SM) and post-synthesis modification (post-SM). For pre-SM, the functionality is firstly introduced into the monomer, which can be further used to synthesize functionalized COFs. Although the obtained functionalized COFs possess abundant functionalities within the frameworks, this approach is not appropriate for all kinds of COFs and it is even incompatible with the synthesis of some COFs, significantly limiting widespread applications. For post-SM, pre-prepared COFs can be used as the scaffold and then assembled with a variety of functionalities. In contrast, post-SM can introduce some task-specific functionalities that are impossible to be incorporated into the frameworks of COFs *via* pre-SM. However, due to the inherent deficiencies of solid–liquid reactions, it is sometimes difficult to achieve the complete functionalization of the frameworks from the inside out. In addition, when the sizes of the functionalized molecules exceed the pore size of the COFs, only surface functionalization can be realized.

### 5.1 Pre-SM

Active sites or functional groups can be introduced into the organic monomers in advance before they can be used for the construction of COFs. In this way, the active sites can be uniformly distributed throughout the COFs, which is beneficial for diverse applications such as heterogeneous catalysis and separation. Theoretically, any active group can be designed and incorporated into the monomers for the construction of functionalized COFs, although synthesis conditions need to be optimized on a case-by-case basis.

#### 5.1.1 Pre-synthesis covalent modification

**Fig. 42:**
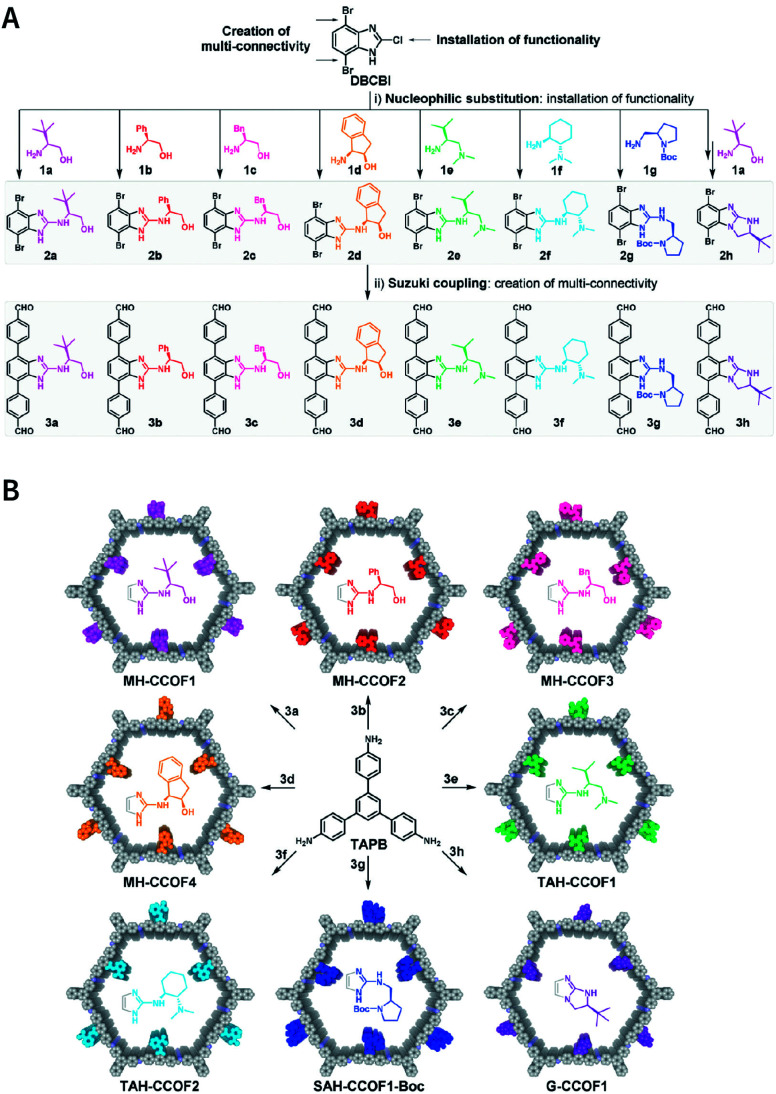
Construction of combinatorial library of CCOFs. (A) Divergent synthesis of chiral monomers from DBCBI by nucleophilic substitution and Suzuki coupling. (B) Divergent synthesis of CCOFs by imine formation. Adapted with permission.^263^ Copyright 2019, Wiley-VCH Verlag GmbH & Co. KGaA, Weinheim.

One of the important functions of pre-SM is to integrate chiral groups into the monomers, affording chiral COFs (CCOFs) for chiral separation and catalysis applications. For example, the CCOFs reported in 2016 were used for chiral separation.^262^ First, 2,4,6-trihydroxybenzene-1,3,5-tricarbaldehyde was functionalized with (+)-*O*,*O*′-diacetyl-l-tartaric anhydride by an esterification reaction to form the chiral-functionalized monomer of CTp. Then, *p*-phenylenediamine, 2,5-dimethylbenzene-1,4-diamine, and [1,1′-biphenyl]-4,4′-diamine were condensed with CTp to afford the CCOFs of CTpPa-1, CTpPa-2, and CTpBD, respectively. These CCOFs exhibited highly regular chiral pore environments, while their bound capillary columns showed higher resolution for the separation of enantiomers. In addition, for asymmetric catalysis, a CCOF library^263^ was formulated based on 4,7-dibromo-2-chloro-1*H*-benzo[*d*]imidazole (DBCBI) as the platform molecule through nucleophilic substitution, Suzuki coupling reaction, and imine formation (Fig. 42). This functional COF combinatorial library enables further structure–activity relationship investigation and high-throughput screening.

In addition to the above representative examples, other functional groups, such as alkyl chain,^68,264–266^ thioether,^267^ free radical,^268^ chiral pyrrolidine,^269,270^ imidazolium,^271^ 8-hydroxyquinoline,^272^ and crown ether,^273^ are also introduced into COFs *via* pre-SM, which considerably enriches the types and applications of COFs.

#### 5.1.2 Pre-synthesis coordination modification

When using monomers containing coordination atoms to construct COFs, metal ions can be incorporated into the monomers in advance, which is called pre-synthesis coordination modification. Some porphyrin- and phthalocyanine-based COFs have been metallized by using this method (Table 4).^274–286^ However, most coordination compound molecules cannot be stabilized within the COFs under harsh solvothermal conditions, significantly limiting their widespread applications.

**Table 4:** Pre-synthesis coordination modification of porphyrin- and phthalocyanine-based COFs

Chelating ligand in COFs	Metal ion	Property and application	Ref.
Phthalocyanine	Ni^2+^	High photoconductivity	274
Phthalocyanine	Ni^2+^	High intrinsic conductivity	275
Phthalocyanine	Co^2+^	Hydrogen storage	276
Phthalocyanine	Co^2+^	Hydrogen storage	277
Phthalocyanine	Co^2+^	Electrocatalytic CO_2_ reduction	278
Phthalocyanine	Zn^2+^	Ordered heterojunctions	279
Phthalocyanine	Zn^2+^/Co^2+^/Cu^2+^	Photocurrent gain	280
Phthalocyanine	Zn^2+^/Cu^2+^	Electronic properties	281
Porphyrin	Zn^2+^	Ambipolar charge transportation	282
Porphyrin	Zn^2+^	Optoelectronics devices	229
Porphyrin	Pd^2+^	Antibiosis	283
Porphyrin	Pd^2+^	Photocatalysis	284
Porphyrin	Zn^2+^/Ni^2+^/Cu^2+^	Nonlinear optical switching	285
Porphyrin	Zn^2+^/Ni^2+^/Cu^2+^	CO_2_ photoreduction	286

### 5.2 Post-SM

Similar to the post-SM of MOFs,^287,288^ the post-SM of COFs generally involves the embedding of reactive functional groups into the frameworks of pre-synthesized COF materials to achieve task-specific applications *via* different post-SM strategies, such as post-synthesis covalent modification, post-synthesis functional group conversion, bonding defect functionalization (BDF), and post-synthesis linkage transformation. Here, we will provide detailed summary and discussion of each strategy, expecting to give the readers a better understanding of this promising post-SM approach.

#### 5.2.1 Post-synthesis covalent modification

**Fig. 43:**
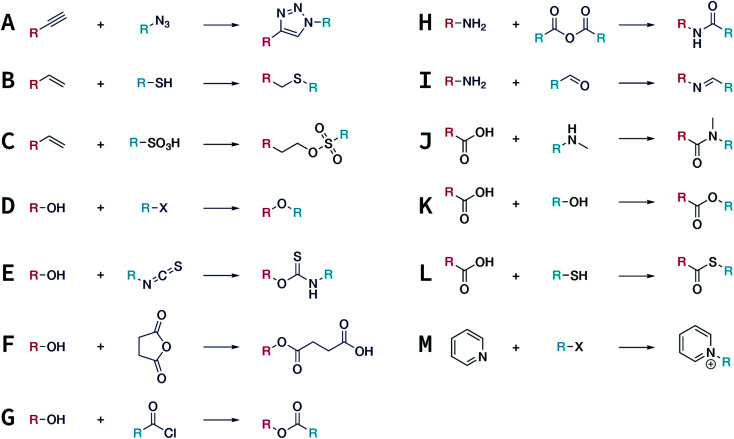
Typical covalent bonds adopted in the covalent post-SM of COFs. (A) Azide–alkyne cycloaddition. (B) Thiol–ene click reaction. (C) Electrophilic addition reaction of the vinyl group with sulfonic acid. (D) Williamson ether synthesis reaction. (E) Isothiocyanation of hydroxyl. (F) Esterification of hydroxyl with cyclic anhydrides. (G) Esterification of hydroxyl with acyl halides. (H) Amidation of the amino group. (I) Imidization of the amino group. (J) Amidation of the carboxyl group. (K) Esterification of the carboxyl group. (L) Thioesterification of the carboxyl group. (M) Formation of pyridinium salt.

With regard to covalent post-SM, the desired functional groups or active sites can be integrated within the frameworks of pre-synthesized COFs *via* covalent bonds. Till now, the reaction sites within the COF structures mainly include ethynyl, vinyl, hydroxyl, amino, and carboxyl groups (Fig. 43).

**Fig. 44:**
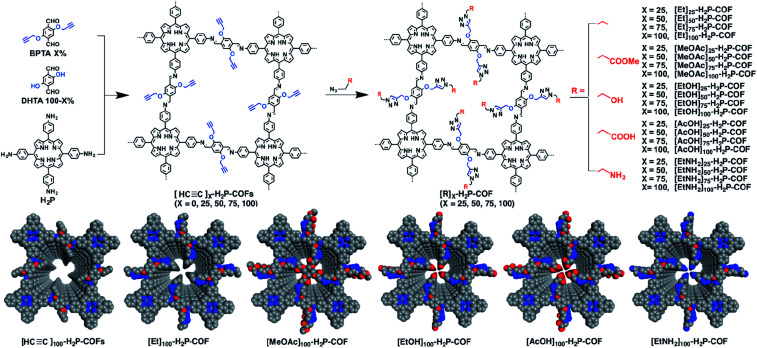
Post-SM of porphyrin-based COFs with various functional groups *via* click reactions. Adapted with permission.^289^ Copyright 2015, American Chemical Society.

To achieve the covalent modification of COFs, a high-yield modification reaction under mild conditions is highly desired, *e.g.*, click chemistry. Jiang *et al.* synthesized [HC≡C]_*x*_-H_2_P-COFs containing content-tunable and reactive ethynyl groups in the pores by the imine condensation reaction of 2,5-dihydroxyterephthalaldehyde, 2,5-bis(ethynyloxy)terephthalaldehyde, and 4,4′,4′′,4′′′-(porphyrin-5,10,15,20-tetrayl)tetraaniline (Fig. 44).^289^ Quantitative click reactions between the ethynyl units and azide compounds were performed with CuI as the catalyst to anchor the desired groups into the pores. The groups with different hydrophilicities and acid–base properties (including ethyl, ester, hydroxyl, carboxyl, and amino groups) were successfully incorporated into the COFs with controllable loading contents. The effects of functional groups on their gas adsorption properties were confirmed by CO_2_ adsorption measurements. For example, the CO_2_ adsorption capacity could reach up to 157 mg g^−1^ at 273 K and 1 bar for functionalized COFs with 50% amino groups, which was three times higher than that of pristine COFs. Covalent post-SM based on a click reaction to form a triazole ring is an extremely versatile and efficient method that enables the development of custom-built COFs with tunable porosities and functionalities while maintaining their high crystallinity.^164,290–298^

**Fig. 45:**
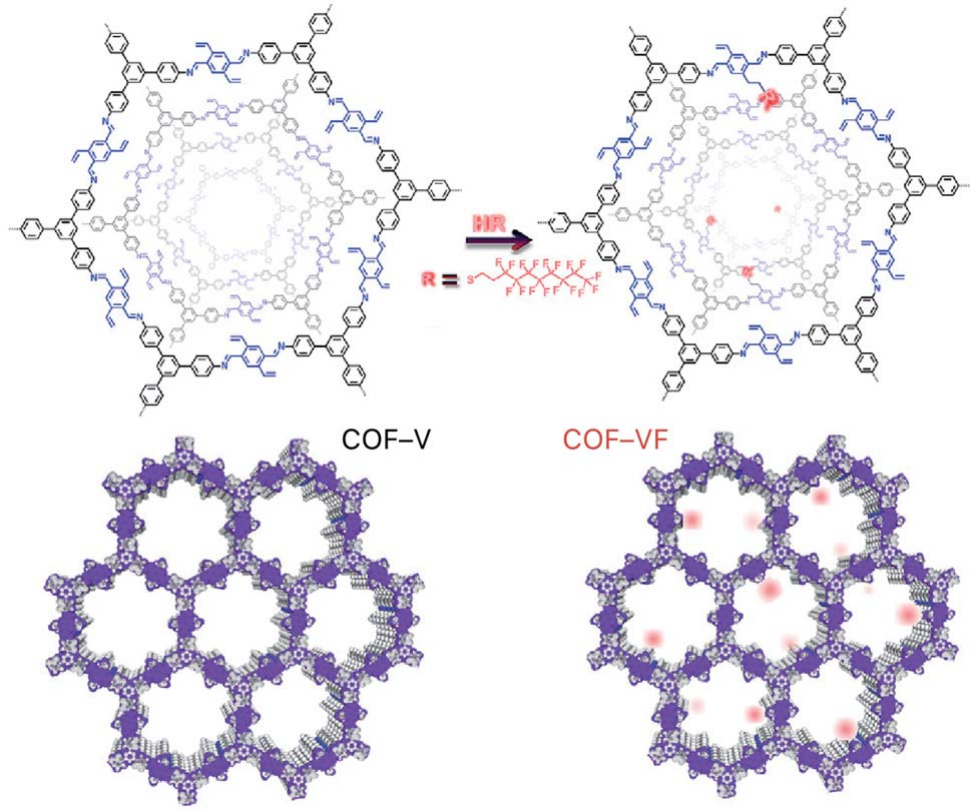
Thiol–ene click synthesis of perfluoride-functionalized COF-VF. Adapted with permission.^299^ Copyright 2018, Elsevier Inc.

The click reaction of vinyl with thiol is another typical click-chemistry-based post-SM method. For instance, vinyl-functionalized COF-V and 3,3,4,4,5,5,6,6,7,7,8,8,9,9,10,10,10-heptadecafluorodecane-1-thiol with low surface free energy reacted in the presence of azobisisobutyronitrile (AIBN) as the catalyst to yield perfluoroalkane-modified COF-VF (Fig. 45).^299^ Since the enhanced hydrophobicity of the materials can be realized at the expense of porosity and crystallinity of COF-V, by optimizing the reaction conditions, the modification rate of vinyl can be controlled at 4% to balance the hydrophobicity and inherent properties of COF-V. The water contact angle increased from 113° for COF-V to 167° for COF-VF, demonstrating the superhydrophobicity of COF-VF. In addition, other fascinating examples, such as ethane-1,2-dithiol,^300,301^ (4-mercaptophenyl)boronic acid,^302^ 4-mercaptobenzoic acid,^303^ and glutathione,^253^ have been modified into COFs by the thiol–ene reaction.

Noteworthily, electrophilic addition reactions of vinyl groups, such as the reaction of vinyl groups with sulfonic acids to generate sulfonate esters, can also be used for the post-SM of COFs. For example, Lv *et al.* prepared sulfonic-group-modified COFs with the maximum adsorption capacity for diclofenac of up to 770 mg g^−1^.^304^

**Fig. 46:**
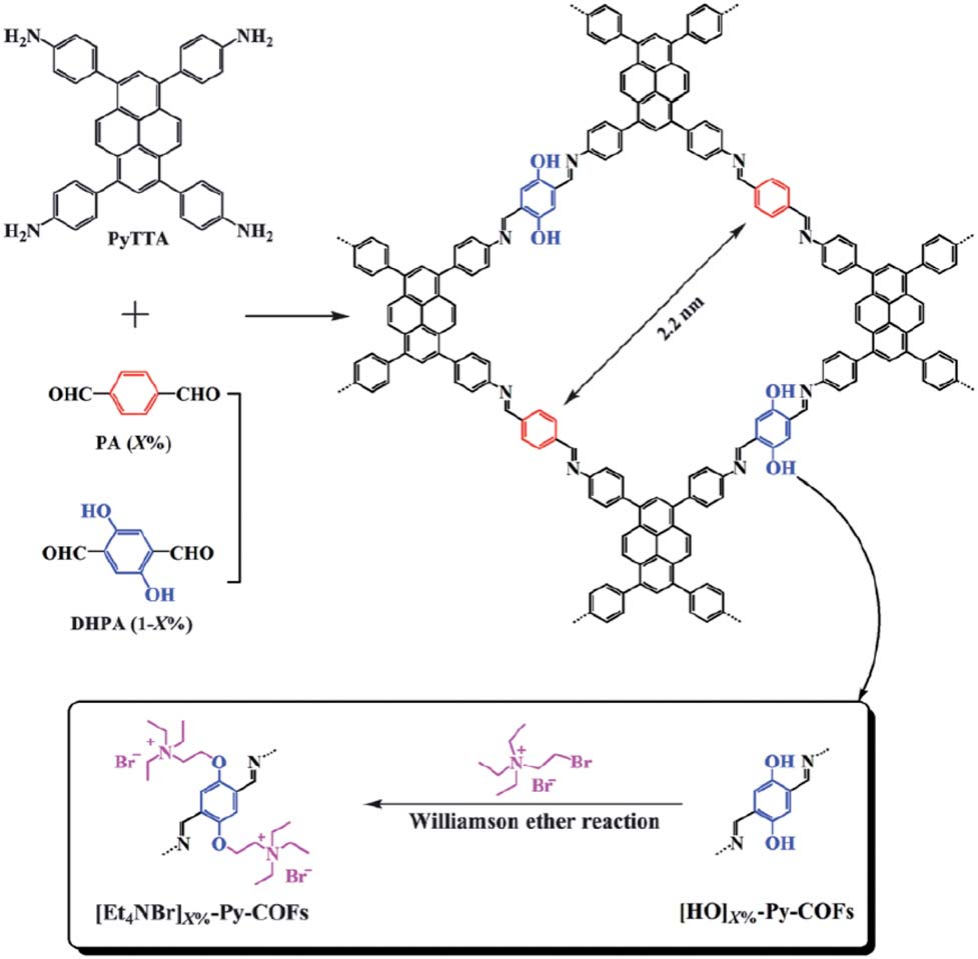
Williamson ether synthesis of IL-functionalized [Et_4_NBr]_*X*%_-Py-COFs for catalyzing the formylation reaction. Adapted with permission.^305^ Copyright 2016, The Royal Society of Chemistry.

The hydroxyl group has become a promising reaction center for post-SM because of its versatile reaction chemistry (Fig. 46). By the Williamson ether synthesis reaction of hydroxyl groups with halohydrocarbon, the etherification of COFs can be achieved. For example, Gao *et al.* synthesized hydroxyl-rich [HO]_*X*%_-Py-COFs using X-shaped tetraamine and two linear dialdehydes as the monomers.^305^ Subsequently, [HO]_*X*%_-Py-COFs were reacted with a quaternary ammonium salt for fixing the IL on the pore walls of the COFs. The obtained [Et_4_NBr]_*X*%_-Py-COFs could catalyze the formylation of amine with CO_2_ and phenylsilane under metal-free conditions. Besides, the post-SM reaction of the hydroxyl group with iodine-substituted perfluoroalkane could convert hydrophilic COF-DhaTab into hydrophobic COF-DTF.^306^

**Fig. 47:**
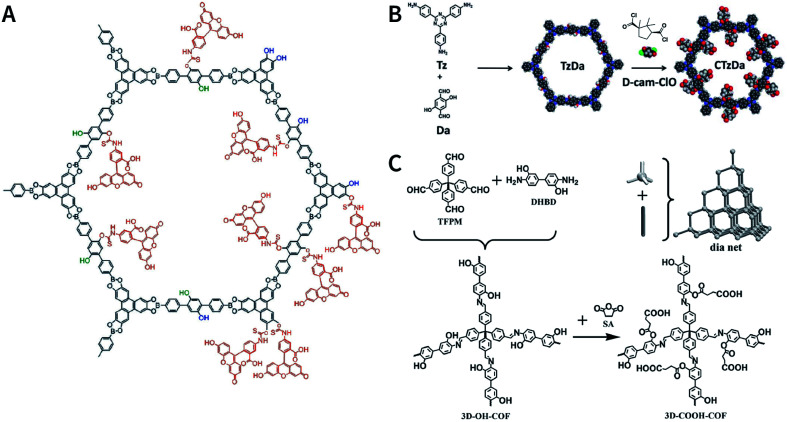
Post-SM *via* esterification of hydroxyl groups. (A) FITC-functionalized T-COF-OFITC as luminescent materials. Adapted with permission.^307^ Copyright 2017, The Royal Society of Chemistry. (B) Chiral d-(+)-camphorate-modified CTzDa COF. Adapted with permission.^130^ Copyright 2020, The Royal Society of Chemistry. (C) Introducing carboxyl groups into 3D COFs using succinic anhydride. Adapted with permission.^310^ Copyright 2018, Wiley-VCH Verlag GmbH & Co. KGaA, Weinheim.

Esterification is another effective strategy for hydroxyl modification. For example, Bein *et al.* studied the reaction of T-COF-OH with fluorescein isothiocyanate (FITC) in acetone to modify nonfluorescent T-COF-OH into T-COF-OFITC with green-light emission *via* a thiocarbamate bond (Fig. 47A).^307^ Another attempt involved the esterification of hydroxyl groups with acyl halides. Chiral d-(+)-camphoric acid dichloride (d-cam-ClO) has been loaded into the pores of TzDa COF by this reaction; the resulting CTzDa COFs were used as an adsorbent for the chiral isolation of amino acid enantiomers (Fig. 47B).^130^ The hydroxyl group can also be esterified by an anhydride and the versatility of this method has been demonstrated by the esterification of hydroxyl groups in 2D COFs^308,309^ and 3D COFs.^310^ As shown in Fig. 47C, the ring-opening esterification reaction of succinic anhydride not only esterifies the hydroxyl group, but also introduces a carboxyl group into the COFs.^310^

There are two different ways for performing the covalent post-SM of amino groups, *i.e.*, amidation and imidization. For example, the amidation reaction between TpPa-NH_2_ and 4,4′-(ethane-1,2-diyl)bis(morpholine-2,6-dione) can integrate ethylenediaminetetraacetic acid (EDTA) into the pores, thereby improving the heavy-metal-ion adsorption capacity of the COF material.^311^ TpBD(NH_2_)_2_-COF-containing amino groups enable the reaction with acetic anhydride to achieve the acetylation of the pore walls.^312^ In addition, TpBD(NH_2_)_2_ COFs can be functionalized by the imine condensation reaction of the amino group with (2-formylphenyl) boronic acid.^252^

**Fig. 48:**
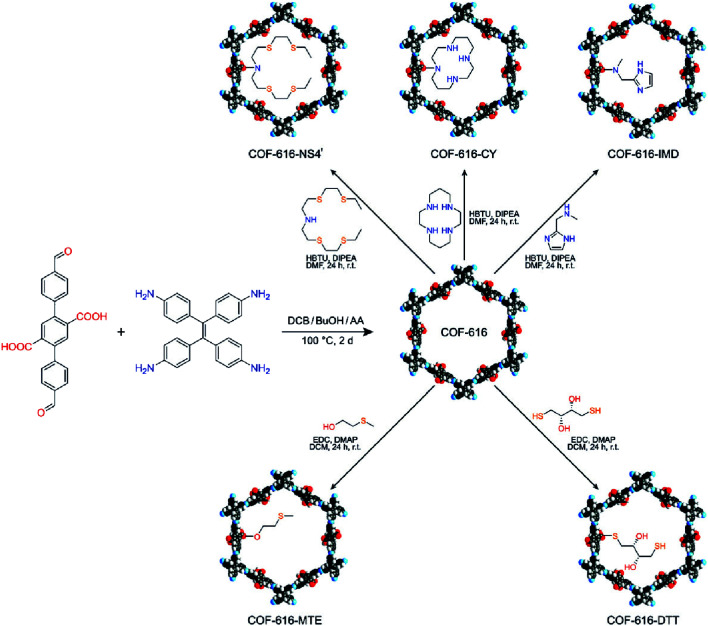
Post-SM of carboxyl-functionalized COF-616 *via* amidation, esterification, and thioesterification. Adapted with permission.^313^ Copyright 2020, Wiley-VCH Verlag GmbH & Co. KGaA, Weinheim.

Very recently, the modification of carboxyl groups was successfully reported by Yaghi and co-workers.^313^ With the help of certain condensating agents, such as 1-(bis(dimethylamino)methylene)-1*H*-benzo[*d*][1,2,3]triazole-1-ium 3-oxide hexafluorophosphate (HBTU) and 3-(((ethylimino)methylene)amino)-*N*,*N*-dimethylpropan-1-amine (EDC), the post-SM of carboxyl-functionalized COF-616 can be realized *via* amidation, esterification, and thioesterification at room temperature, and several chelating groups can be introduced into COF-616 to afford functional materials for the adsorption of pollutants in water (Fig. 48).

#### 5.2.2 Post-synthesis functional group conversion

**Fig. 49:**
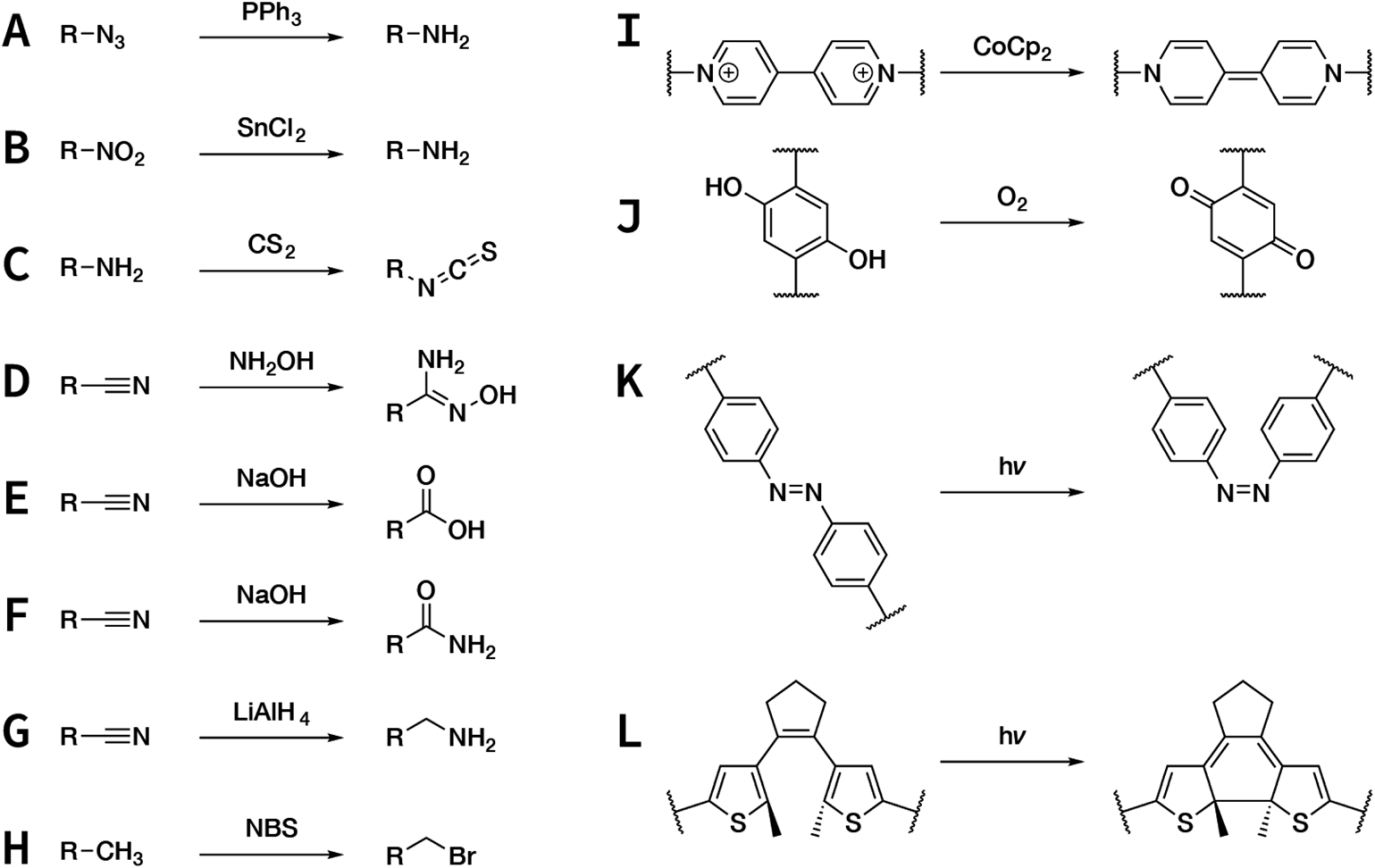
Post-SM of COFs *via* post-synthesis functional group conversion.

Although the amino group is a good candidate for post-SM, the *de novo* preparation of imine-bonded COFs containing free amino groups is a formidable challenge. Post-synthesis functional group conversion (Fig. 49) is regarded as a feasible choice to introduce amino groups into imine-linked COFs.

**Fig. 50:**
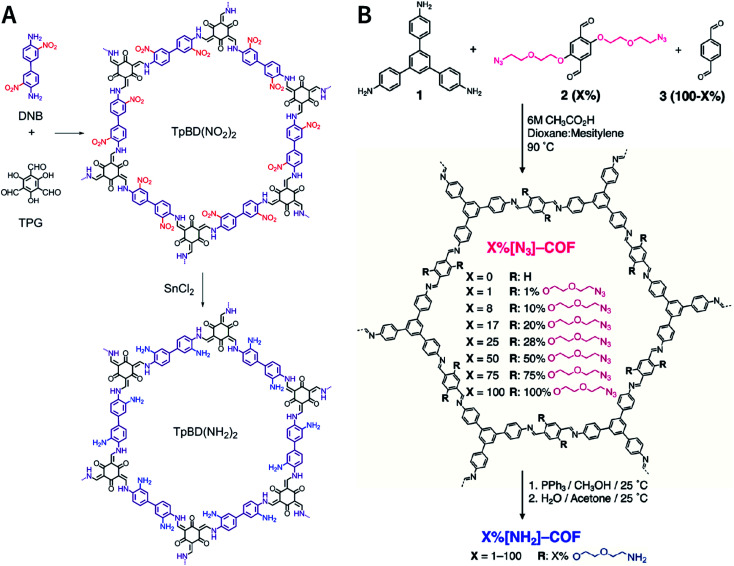
Inserting amino groups into COFs by the reduction of nitro and azide groups. (A) Adapted with permission.^312^ Copyright 2016, American Chemical Society. (B) Adapted with permission.^314^ Copyright 2018, American Chemical Society.

In particular, the reduction of azide and nitro groups is a feasible method to insert amino groups into COFs *via* post-SM. For example, it is difficult to prepare TpBD(NH_2_)_2_ COF by the direct reaction of [1,1′-biphenyl]-3,3′,4,4′-tetraamine and 2,4,6-trihydroxybenzene-1,3,5-tricarbaldehyde, because the four amino groups of the former can randomly react with the trialdehyde monomer and interfere with the formation of a periodic ordered network structure. However, using 3,3′-dinitro-[1,1′-biphenyl]-4,4′-diamine as the precursor, TpBD(NO_2_)_2_ COF was initially prepared (Fig. 50A); subsequently, SnCl_2_ was used to reduce the nitro groups to obtain TpBD(NH_2_)_2_ COF with high crystallinity.^312^ Dichtel *et al.* reported imide-linked COFs containing different amounts of amino groups by reducing the azide groups on the monomer side chain (Fig. 50B).^314^ More importantly, the obtained amino groups could be further modified by covalent modification^252,311,312^ or functional group conversion (*e.g.*, conversion to isothiocyanate^315^) to obtain other types of functional COF materials.

**Fig. 51:**
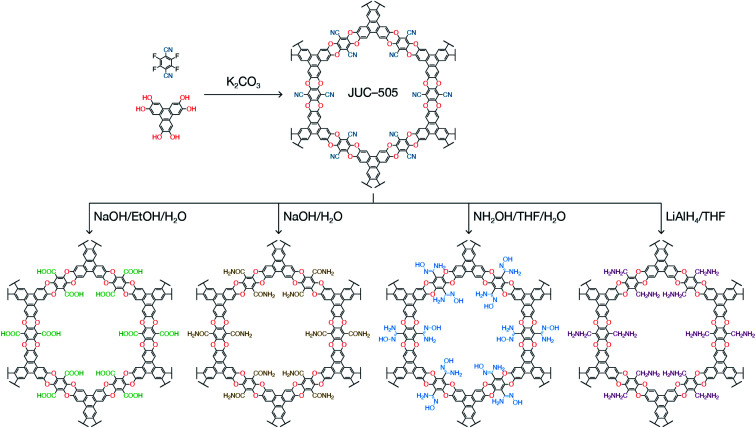
Dioxin-linked JUC-505 and their post-SM of –CN.

Post-SM based on cyano-chemistry has been fully proven by using COF JUC-505 (also known as COF-316) as the model.^79,80^ JUC-505 was synthesized by the irreversible nucleophilic aromatic substitution reaction between 2,3,5,6-tetrafluoroterephthalonitrile and triphenylene-2,3,6,7,10,11-hexaol, which forms 1,4-dioxin linkages. The cyano groups within the JUC-505 framework can be converted to carboxylic acid, amide, amidoxime, and methanamine in NaOH/ethanol/water, NaOH/water, NaOH/ethanol/water, hydroxylamine/tetrahydrofuran/water, and LiAlH_4_/tetrahydrofuran solution (Fig. 51). Functional group conversion based on the reaction of cyano groups with hydroxylamine has also been demonstrated in acrylonitrile-linked TP-COF,^316^ β-ketoenamine-linked COF-TpDb, and COF-TpAab,^317^ which shows the application potential of radionuclide sequestration.

In addition to the aforementioned generic examples of functional group conversion, there are some other post-SM examples that are only compatible with specific COF structures to some extent. For example, methyl bromination,^318^ viologen reduction,^319^*p*-phenol oxidation,^168^ photoinduced *cis*–*trans* isomerization,^320^ and photoinduced pericyclic reaction.^321^

Above all, a considerable number of organic chemical reactions can be carried out on COF platforms to achieve functional group conversion, which can considerably enrich the types of functional groups in COFs and expand the applications of advanced COF-based functional materials.

#### 5.2.3 BDF

Despite the high crystallinity of COFs, defects are still inevitable, such as vacancy defects due to monomer deficiency in periodic ordered structures, edge dislocations because of squeezing or stretching of lattices, extrinsic defects as a result of the doping of extraneous materials (*e.g.*, dust). Among them, the bonding defect located at the crystal grain edge and caused by unreacted functional groups on the monomer remains reactive, providing an opportunity for surface post-SM. This covalent modification method using the bonding defects of COFs is called BDF.

**Fig. 52:**
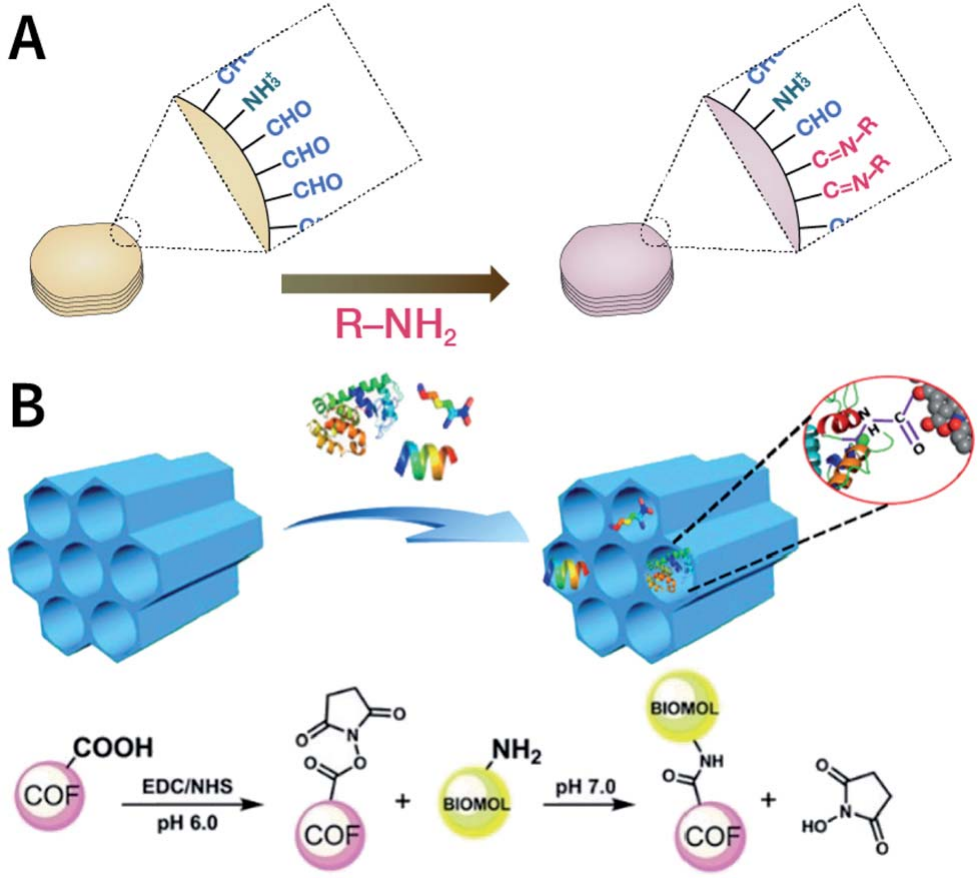
(A) BDF of imine-linked COFs. (B) Grafting biomolecules on the surface of NKCOF-1 COF containing carboxyl groups *via* BDF. Adapted with permission.^323^ Copyright 2018, Wiley-VCH Verlag GmbH & Co. KGaA, Weinheim.

The presence of free amino and aldehyde groups in imine-COFs is a widely accepted consensus, as revealed by ssNMR and FTIR measurements. For instance, the ssNMR peak of the aldehyde group in LZU-1 COF is at 191 ppm and the FTIR characteristic peak is at 1695 cm^−1^; in contrast, these peaks are located at 191 ppm and 1682 cm^−1^, respectively, for TPB-DMTP-COF. Consequently, Dong *et al.* further demonstrated that the aldehyde groups on the surface of imine-linked LZU-1 COF^216^ and TPB-DMTP-COF^220^ can graft with amino-containing functional molecules *via* Schiff-base condensation reaction (Fig. 52A). Similarly, by the immobilization of polyvinylamine onto the frameworks of LZU-1 COF, the obtained polyCOF hybrid materials with the side chain of amino group exhibited superior CO_2_/N_2_, CO_2_/CH_4_, and CO_2_/H_2_ gas separation selectivity.^322^

In addition, imide-linked NKCOF-1, which was generated from 1*H*,3*H*-benzo[1,2-*c*:4,5-*c*′]difuran-1,3,5,7-tetraone and 4,4′,4′′-(1,3,5-triazine-2,4,6-triyl)trianiline, has carboxyl groups on its surface and can be used for BDF. Generally, the formation of NKCOF-1 occurs in two steps. First, the anhydride reacts with the primary amine *via* ring opening to form amic acid. When the synthesis temperature rises above 150 °C, amic acid gets further dehydrated to form imide linkages. During the two-step condensation reaction, a portion of the carboxyl groups is not condensed with the amino groups, thereby resulting in bonding defects. Ma *et al.* found that when the synthesis temperature was 200 °C, the content of carboxyl groups was approximately 5.7% in NKCOF-1, as determined by the acid–base titration analysis.^323^ Furthermore, these carboxyl groups continued to react with the amino groups, making NKCOF-1 as the ideal platform for the covalent modification of biomolecules, such as lysozyme, tripeptide Lys–Val–Phe, and lysine (Fig. 52B).

#### 5.2.4 Truncation unit functionalization (TUF)

**Fig. 53:**
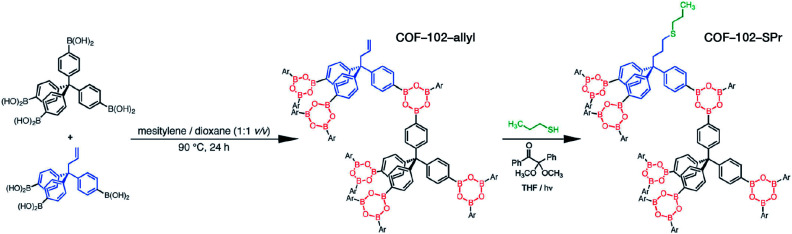
TUF of COF-102-allyl *via* the thiol–ene click reaction. Adapted with permission.^325^ Copyright 2013, The Royal Society of Chemistry.

The concepts of truncation unit and TUF dates back to 2012.^324^ Dichtel *et al.* proposed the truncation unit strategy for the first time to introduce task-specific functional groups into COFs. The tetrahedral monomer (methanetetrayltetrakis(benzene-4,1-diyl))tetraboronic acid that self-condenses to form COF-102 was modified *via* the substitution of one of its four arylboronic acid moieties by the appointed functional group. Thereafter, the resulting triangular triboronic acid molecule was co-condensed with the original tetraboronic acid monomer to afford truncation-unit-containing COF-102. When the incorporated truncation unit is (but-3-ene-1,1,1-triyltris(benzene-4,1-diyl))triboronic acid, the vinyl group is introduced into COF-102, and the covalent attachment of other molecules can be obtained by the thiol–ene click reaction of the vinyl group with the sulfhydryl group (Fig. 53).^325^

The feasibility of this highly inspiring method has been proven by subsequent reports and studies. For example, partly replacing 1,4-phenylenediboronic acid with 4-boronobenzoic acid to synthesize COF-5 can introduce carboxyl groups into COF-5. At room temperature, EDC-activated carboxyl groups can easily react with amino-functionalized ATTO 633 fluorescent dye to endow COF with near-infrared fluorescence properties.^204^ Further, imine-linked B-COF containing the truncation unit of phenylboronic acid can be obtained by the co-condensation of benzidine, 2,4,6-trihydroxybenzene-1,3,5-tricarbaldehyde, and (4-aminophenyl)boronic acid. Due to the high affinity of phenylboronic acid and diol, the resulting B-COF can be used to selectively enrich riboflavin, luteolin, and pyrocatechol for mass spectrometry analysis; further, the limits of detection are as low as femtogram per milliliter.^326^ In addition, covalently doping photoactive hydroxyquinoline–Pt(ii)^327^ and protocatechualdehyde–Fe(iii)^328^ complexes into imine-linked COFs as the truncation units have also been reported.

**Fig. 54:**
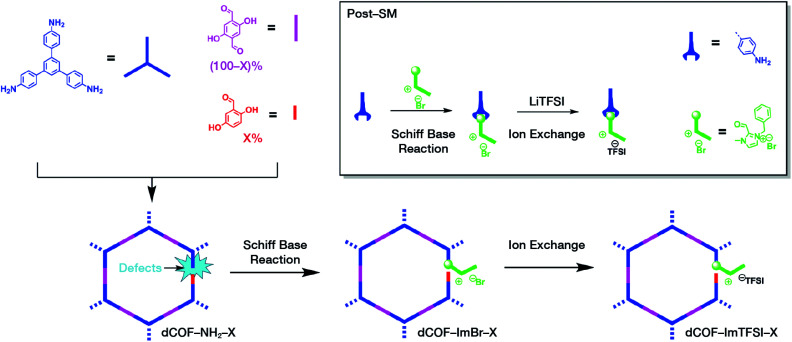
Synthesis and post-SM of dCOF-NH_2_-X with exposed amino groups. Adapted with permission.^329^ Copyright 2020, Wiley-VCH Verlag GmbH & Co. KGaA, Weinheim.

Very recently, Han *et al.* synthesized dCOF-NH_2_-X with exposed amino groups (Fig. 54) using a three-component condensation system with 1,3,5-tris(4-aminophenyl)benzene and 2,5-dihydroxyterephthalaldehyde as the COF monomers and 2,5-dihydroxybenzaldehyde as the truncation unit.^329^ More importantly, the amount of free amino groups could be precisely regulated by changing the amount of truncation unit. As expected, free amino groups can be decorated by ILs with aldehyde groups *via* imine condensation. The resulting materials possess not only well-defined pore channels that provide pathways for ion transport, but also an optimized pore environment containing ILs for higher ion conduction, which can be used as solid-state electrolytes with a wide temperature range from 303 to 423 K. Besides, the authors synthesized dCOF-CHO-X with free aldehyde groups using 5′-phenyl-[1,1′:3′,1′′-terphenyl]-4-amine as the truncation unit, which can be used as post-SM platforms for amino-containing functional molecules. It should be noted that unlike several earlier examples, in this work, the truncation unit itself does not contain reaction sites and the free reaction groups are entirely derived from COF monomers, implying the flexibility and feasibility of this strategy.

In conclusion, by simply adding a truncation unit to the reaction system without changing the crystallization conditions of COF synthesis, the truncation unit can be uniformly doped into the entire lattice. The reasons that the truncation unit can provide possibilities for post-SM are as follows. (i) The truncation unit itself may contain anchoring sites for post-SM as long as these sites do not participate in the condensation reaction to form COFs. (ii) The doped truncation unit results in the failure of linkage formation at the truncation location, thereby inducing well-controlled defects and active anchoring sites from the COF monomers. (iii) The content of the truncation unit was determined by the feed ratio of the two monomers, which can prevent higher contents of truncation units while maintaining higher crystallinity and porosity of the pristine COFs. Therefore, the TUF strategy can effectively overcome the limitation of the BDF strategy.

#### 5.2.5 Post-synthesis linkage transformation

**Fig. 55:**
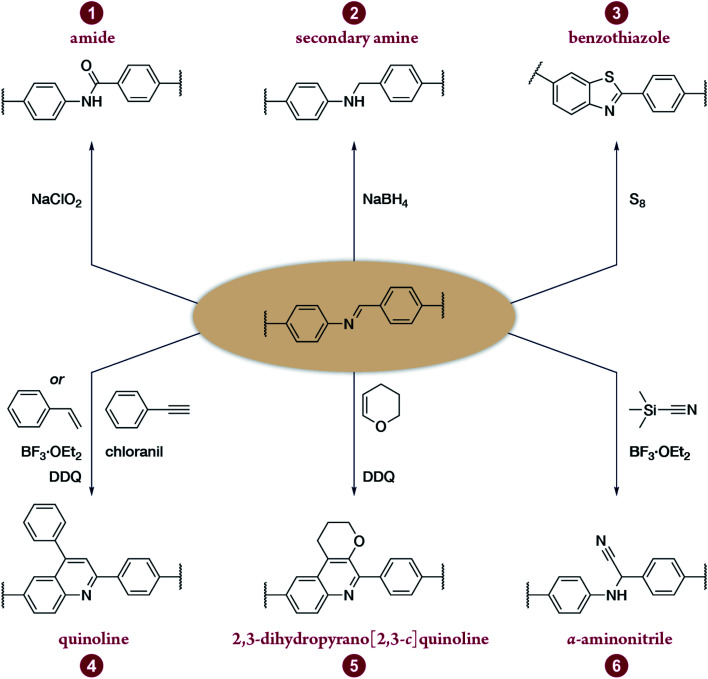
Post-synthesis linkage conversion of C=N-linked COFs.

Essentially, the linkage of COFs is a chemical bond, and it is also widespread in reaction chemistry, providing a unique channel for fine-tuning the pore environment *via* post-synthesis linkage transformation (Fig. 55). More importantly, this approach makes it possible to prepare COFs with new linkages, particularly for some linkages that are extremely difficult or even impossible to be synthesized *de novo*. So far, the diversity of linkage modification has been realized in various Schiff-base COFs.

The oxidation of C=N in COFs was reported for the first time in 2016.^330^ Two imide-linked 2D COFs, namely, TPB-TP-COF and 4PE-1P-COF, were quantitatively oxidized to amides by NaClO_2_ in an acetic acid solution. The additionally added 2-methyl-2-butene was used to remove the hypochlorous acid generated from the reaction system. Both PXRD and N_2_ adsorption measurements of the COF materials indicated that the amidation process did not lead to significant changes in the COF structures. Amide-linked COFs exhibited enhanced chemical stability in acid and base solutions. For example, after treatment in hydrochloric acid (12 M) or NaOH (1 M) solutions for 24 h, the crystallinity of the amide-linked COFs was retained, while the corresponding imine-linked COFs were dissolved or turned into amorphous polymers. The NaClO_2_ oxidation of 3D COFs based on tetrahedral tetraamine and chiral tetraaldehyde monomers^331^ also achieved the conversion of imines to amides, suggesting the generalization of this method.

**Fig. 56:**
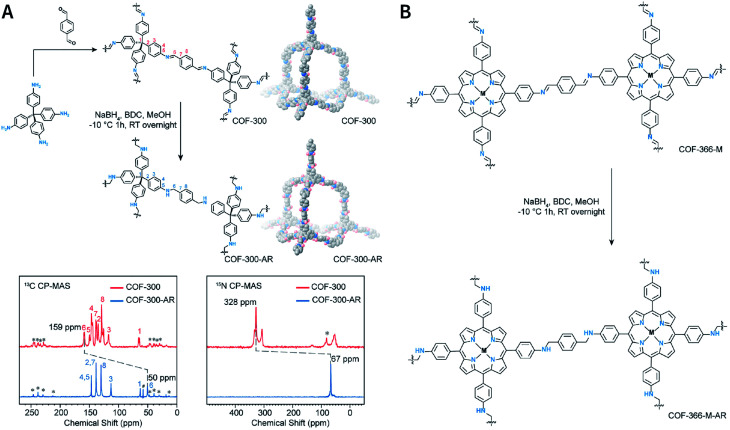
Reduction of imine-linked COFs to amines with NaBH_4_. (A) COF-300 and COF-300-AR. Inset: ^13^C and ^15^N CP-MAS ssNMR. (B) COF-366-M and COF-366-M-AR (M = Cu, Zn). Adapted with permission.^332^ Copyright 2018, Elsevier Inc.

NaBH_4_ can reduce imine- to amine-linked COFs, as demonstrated by COF-300 and COF-366-M (M = Cu, Zn).^332^ For instance, after the reduction of COF-300 with NaBH_4_ in methanol, the ^13^C and ^15^N CP-MAS ssNMR of the obtained COF-300-AR verified the quantitative conversion of imine to amine (Fig. 56). The peak of imine carbon at 159 ppm completely disappeared, and a secondary amine carbon emerged at 50 ppm. The imine nitrogen peak at 328 ppm disappeared, while the secondary amine nitrogen appeared at 67 ppm. As expected, the reduction of the C=N bond significantly enhanced the chemical stability of COF-300-AR. When compared with pristine COF-300, on the silver electrode coated with COF-300-AR, the secondary amine bond provided chemisorption sites for the selective adsorption of CO_2_ by forming carbamates, thereby improving the faradaic efficiency of CO_2_ reduction. Noteworthily, the generated secondary amine linkage can be further modified. For example, the amine linkages underwent an aminolysis reaction with 1,3-propanesultone, affording a sulfonic-acid-functionalized COF.^333^ Moreover, with regard to the applications of COF-300-AR, a recent study confirmed that COF-300-AR can also act as a light-responsive oxidase mimic, which can detect glutathione in HL60 cells when excited by purple light at 400 nm.^334^

**Fig. 57:**
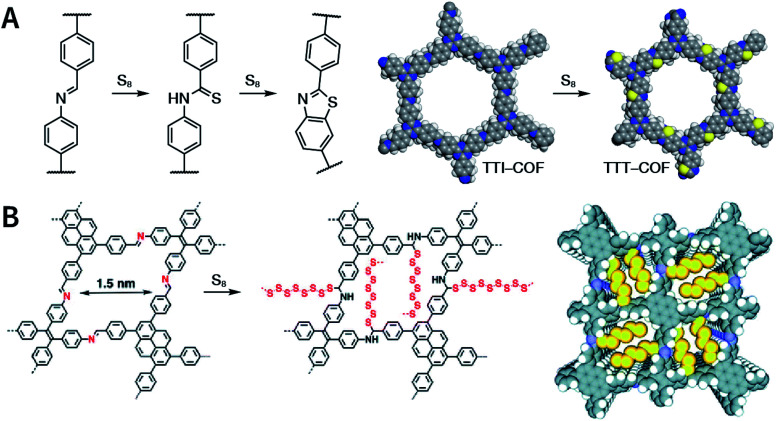
Sulfuration of imine-linked COFs. (A) Conversion of an imine- into a thiazole-linked COF. Adapted under a Creative Commons Attribution 4.0 International License. Copyright 2018, The Author(s).^335^ Published by Springer Nature Limited. (B) Covalent engineering of polysulfide chains on walls for synthesizing energy-storage COF. Adapted with permission.^336^ Copyright 2019, The Royal Society of Chemistry.

Imine-linked TTI-COF reacts with sulfur at higher temperatures to convert the imine bond into a thiazole ring.^335^ During the reaction, first, the imine bond gets oxidized into thioamide and then oxidative cyclization occurs to form thiazole (Fig. 57A). The resulting TTT-COF was stable in hydrazine, NaOH, and NaBH_4_ solutions, while TTI-COF became amorphous under identical conditions, suggesting the contribution of the thiazole ring to the ordered structure of TTT-COF. In addition, when TTI-COF was exposed to the high-energy electron beam of HRTEM, the lattice fringes of TTI-COF gradually decreased with a half-life of 1.22 min; in contrast, TTT-COF was much more stable with a half-life of 2.83 min. This enhanced stability enabled the study of the formation mechanism and crystal defects of COFs by HRTEM. The significance of sulfuration is also reflected in the preparation of energy-storage COFs (Fig. 57B). By exercising reasonable control over the sulfuration temperature, the imine linkage of COFs can be converted into polysulfide-modified thioamide for energy storage.^336^ Polysulfide chains, rather than sulfur guest molecules, can accelerate the redox kinetics and activity, facilitate ion transport, and facilitate charge conduction.

**Fig. 58:**
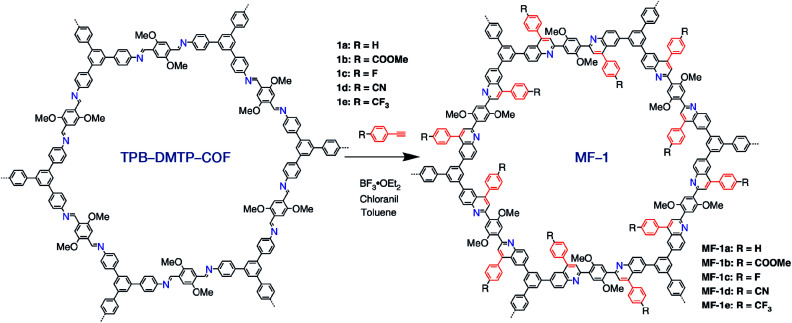
Quinoline-linked COFs formed by aza-Diels–Alder cycloaddition reaction. Adapted under a Creative Commons Attribution 4.0 International License. Copyright 2018, The Author(s).^337^ Published by Springer Nature Limited.

Imine linkage has also been converted to quinoline *via* the Povarov reaction (also known as aza-Diels–Alder reaction).^337^ The reaction of TPB-DMTP-COF, BF_3_·OEt_2_, and chloranil in the presence of toluene with phenylacetylene at 110 °C for 72 h resulted in the formation of deep-yellow quinoline-linked MF-1a COF (Fig. 58). Due to the conversion of dynamic imines to strong quinolines, MF-1a was stable under a variety of extremely harsh chemical conditions, such as strong protonic acid (12 M HCl at 50 °C for 8 h), superacid (98 wt% trifluoromethanesulfonic acid for 72 h), strong base (14 M NaOH in water/methanol at 60 °C for 24 h), strong oxidant (KMnO_4_ in water/acetonitrile for 24 h), and reductant (NaBH_4_ in methanol at 65 °C for 24 h). More importantly, this method also provides a good platform for introducing other functional groups by simply using substituted phenylacetylene for post-SM.

More recently, Dong *et al.* further extended the application scope of this strategy by using phenylethylene instead of phenylacetylene to realize the quinolinization of TPB-DMTP-COF.^107^ Moreover, since olefinic bonds can appear in the ring, when 3,4-dihydro-2*H*-pyran was used for the reaction, the 2,3-dihydropyrano[2,3-*c*]quinoline structure can be introduced into the framework. More importantly, the synthesis can be performed not only gradually through post-SM, but also *in situ* under solvothermal conditions. Therefore, this approach provides an unprecedented new way to build condensed ring-linked COFs. Dong *et al.* used the Strecker reaction for COF post-SM.^107^ With BF_3_·OEt_2_ as the catalyst and DDQ as the dehydrogenated agent, the reaction of trimethylsilane carbonitrile or diethyl phosphorocyanidate with TPB-DMTP-COF converted the imine bond into α-aminonitrile, thereby introducing cyano groups into the framework.

**Fig. 59:**
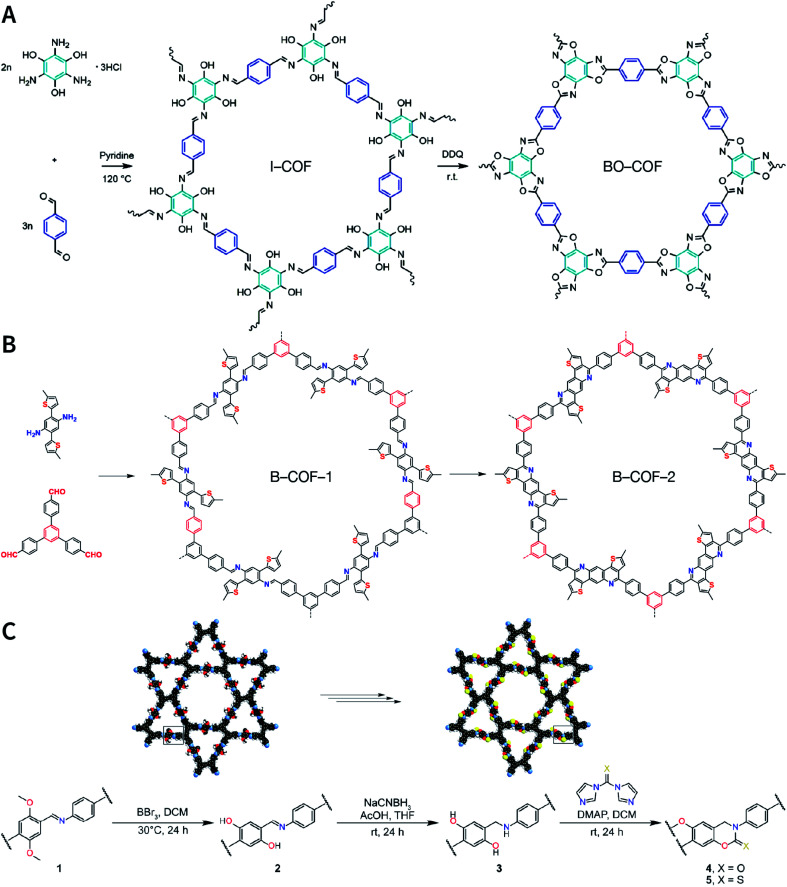
Neighboring-group participation in the post-SM of COFs. (A) Converting unstable imine-linked COFs into stable benzoxazole-linked COFs *via* post-oxidative cyclization. Adapted with permission.^338^ Copyright 2019, American Chemical Society. (B) Construction of fully conjugated B-COF-2 *via* the oxidative cyclization of thiophene and imine. Adapted with permission.^339^ Copyright 2020, American Chemical Society. (C) Preparation of cyclic carbamate- and thiocarbamate-linked COFs *via* multistep post-SM. Adapted with permission.^340^ Copyright 2019, American Chemical Society.

Finally, we emphasize upon the role of neighboring-group participation in the establishment of heterocyclic linkages in COFs. I-COF, a 2D COF formed by the condensation of 2,4,6-triaminobenzene-1,3,5-triol and terephthalaldehyde through Schiff-base condensation, was oxidatively cyclized by DDQ, which was converted into a benzoxazole-linked BO-COF with significantly improved thermal and chemical stability (Fig. 59A).^338^ Further, with regard to B-COF-1, there is a thienyl group in the *ortho* position of the imine bond. With trifluoroacetic acid as the catalyst, B-COF-1 was heated in a sealed tube containing oxygen at 100 °C for 2 days (Fig. 59B). The coupled process of the imine carbon and thiophene β-position was carried out to form a conjugated heterocyclic system.^339^ Furthermore, COF-170 was transformed into cyclic carbamate and thiocarbamate-linked COFs after three steps, namely, demethylation by BBr_3_, imine reduction by NaCNBH_3_, and cyclization by 1,1′-carbonyldiimidazole or 1,1′-thiocarbonyldiimidazole (Fig. 59C).^340^ With regard to these inspiring examples, the hydroxyl and thienyl groups adjacent to the imine linkage were involved in the construction of heterocyclic linkage in post-SM, which finally incorporated benzo[1,2-*d*:3,4-*d*′:5,6-*d*′′]tris(oxazole), thieno[2′,3′:4,5]pyrido[2,3-*g*]thieno[3,2-*c*]quinoline, and 3,4-dihydro-2*H*-benzo[*e*][1,3]oxazin-2-(thi)one heterocyclic structures into the COFs. On the contrary, it is almost impossible to introduce such a complicated heterocyclic system by *de novo* COF synthesis. Notably, for chemistry and materials science concepts, this multistep post-SM process including covalent modification and linkage transformation actually transferred the classic solution of organic chemistry from the flask to the COFs, representing a significant step toward bringing the accuracy of organic solution-phase synthesis to extended solid-state materials.

#### 5.2.6 Post-synthesis monomer exchange

The high crystallinity of most COFs is due to the reversibility of linkages. The reversible condensation reaction allows the monomer to connect and disconnect from the framework under thermodynamically controlled conditions to endow COFs with the self-correcting ability during the formation process, thereby minimizing system energy, maximizing crystallinity, and preventing the formation of amorphous polymers. Similarly, this dynamic reaction also allows COF-to-COF transformation *via* monomer exchange.^341,342^

**Fig. 60:**
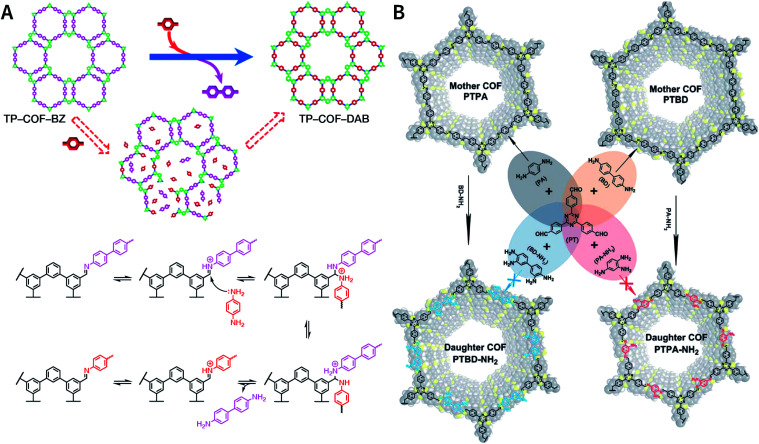
(A) COF-to-COF transformation *via* heterogeneous monomer exchange. Adapted with permission.^343^ Copyright 2017, American Chemical Society. (B) COF-to-COF transformation for the preparation of *de novo* unreachable amino-functionalized PTBD-NH_2_ and PTPA-NH_2_ COFs. Adapted with permission.^344^ Copyright 2018, The Royal Society of Chemistry.

The construction of an imine-linked COF from [1,1′:3′,1′′-terphenyl]-3,3′′,5,5′′-tetracarbaldehyde and benzidine monomers yields TP-COF-BZ with three distinct kinds of pores.^343^ In the presence of acetic acid as the catalyst, TP-COF-BZ was immersed in benzene-1,4-diamine at 120 °C and was almost completely converted to TP-COF-DAB within 4 h (Fig. 60A). Evidently, this COF-to-COF conversion is a heterogeneous process: in the presence of a large amount of benzene-1,4-diamine in solution, benzene-1,4-diamine readily diffuses into the pore and then nucleophilically attacks the imine bond protonated by acetic acid, causing benzidine in this framework to be replaced by benzene-1,4-diamine while retaining its crystallinity. The nucleophilicity of benzene-1,4-diamine is stronger than that of benzidine due to the electron-donating effect of the amino group at the *para* position, and therefore, monomer exchange is difficult to reverse on a macro level. Besides, other impressive examples have also been reported, including 2D-to-2D,^341^ 3D-to-2D,^342^ and 3D-to-3D^342^ COF transformation *via* monomer exchange.

A significant advantage of post-synthesis monomer exchange is that it allows access to *de novo* unreachable COFs. For example, imine-based PTBD-NH_2_ and PTPA-NH_2_ COFs are difficult to fabricate by the direct polymerization of [1,1′-biphenyl]-3,3′,4,4′-tetraamine and benzene-1,2,4-triamine with 4,4′,4′′-(1,3,5-triazine-2,4,6-triyl)tribenzaldehyde, respectively, due to the lack of differential reactivity, but it can be prepared by co-heating PTPA and PTBD COFs with the corresponding monomers *via* monomer exchange (Fig. 60B).^344^

**Fig. 61:**
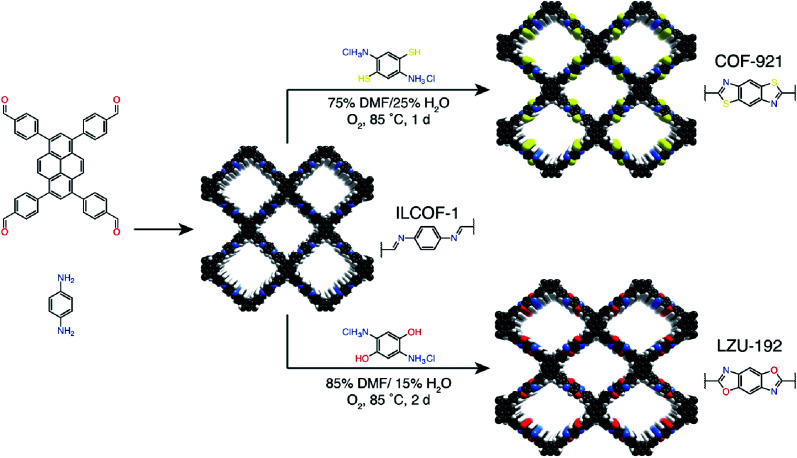
Combining monomer exchange with linkage transformation to synthesize thiazole-linked COF-921 and oxazole-linked LZU-192 COFs. Adapted with permission.^345^ Copyright 2018, American Chemical Society.

Combining monomer exchange with linkage transformation results in the generation of newer linkages (Fig. 61). For example, when ILCOF-1 was treated with 4 equivalents of 2,5-dimercaptobenzene-1,4-diaminium dichloride, benzene-1,4-diamine on the framework was replaced by 2,5-dimercaptobenzene-1,4-diamine, thereby introducing sulfhydryl groups at the *ortho* position of the imine bond. The subsequent oxidation of the material with air at 85 °C in *N*,*N*-dimethylformamide/water by linkage transformation with neighboring-group participation, thiazole-linked COF-921 was generated.^345^ Similarly, when using 4 equivalents of 2,5-dihydroxybenzene-1,4-diaminium dichloride, oxazole-linked LZU-192 (as reported earlier) was generated.

Monomer exchange can also occur between monomers with different types of functional groups.^346^ Imine-linked COF TzBA and terephthaloyl dichloride were immersed in dioxane/mesitylene (2 : 1, v/v) for 2 days at 4 °C, and the [1,1′-biphenyl]-4,4′-dicarbaldehyde on the framework was gradually replaced by terephthaloyl dichloride, leading to the formation of amide-linked JNU-1 COF with irreversible linkage. Due to the formation of the N–H⋯Cl hydrogen bond and the coordinated interaction between O and Au, JNU-1 COF afforded highly selective adsorption capacity for gold recovery, which was not possible in TzBA COF.

#### 5.2.7 Interlayer reaction

The classic electrocyclic reaction has strict requirements on the spatial distance and orientation of the reactant molecules, while regular ordered structures of COFs provide tight constraints on the spatial distribution of monomers. *Via* rational design, electrocyclization reactions between two adjacent layers of monomers become possible.

**Fig. 62:**
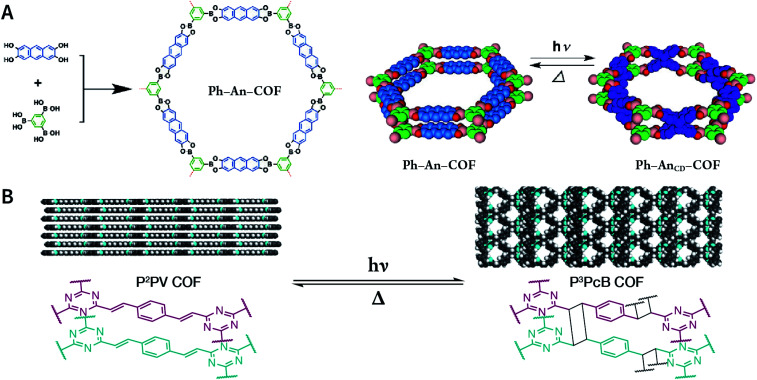
(A) Reversible interlayer [4 + 4] cycloaddition reaction of anthracene-based Ph-An-COF. Adapted under a Creative Commons Attribution Non-Commercial License. Copyright 2015, The Author(s).^347^ Published by Wiley-VCH Verlag GmbH & Co. KGaA, Weinheim. (B) Partially reversible interlayer [2 + 2] cycloaddition reaction of vinylene-linked P^2^PV COF. Adapted with permission.^350^ Copyright 2020, American Chemical Society.

The first example of a reversible COF interlayer reaction is the [4 + 4] cycloaddition reaction of anthracene-based COFs (Fig. 62A).^347^ The boronate-ester-linked 2D COF of Ph-An-COF was obtained by the condensation of anthracene-2,3,6,7-tetraol and benzene-1,3,5-triyltriboronic acid under solvothermal conditions. The AA-stacking structure caused the reactive anthracene to overlap with each other with an appropriate distance of 3.4 Å, which led to 9,10-photodimerization under light irradiation at 360 nm, disrupting the original conjugated system. As this conversion is reversible, the re-aromatization of the anthracene ring upon heating at 100 °C promotes the formation of the original COF. In addition, for anthracene-based IISERP-COF7 with polychromatic light emission, Vaidhyanathan *et al.* observed blue-light quenching due to interlayer [4 + 4] cycloaddition.^348^

Interlayer [2 + 2] cycloaddition of COFs has also been reported. Thomas *et al.* reported changes in the optical properties caused by [2 + 2] cycloaddition based on vinylene-linked V-COF-1.^349^ However, the resulting cycloaddition product was amorphous, and this conversion was not reversible. Partially reversible interlayer [2 + 2] reaction of COFs was reported by Perepichka *et al.* in 2020.^350^ By the alkali-catalyzed aldol condensation of 2,4,6-trimethyl-1,3,5-triazine and terephthalaldehyde, vinylene-linked P^2^PV COF with a layer spacing of 3.4 Å was synthesized (Fig. 62B). When P^2^PV COF was exposed to sunlight, vinylene underwent [2 + 2] cycloaddition, generating cyclobutane rings between the layer with a subsequent increase in the layer spacing to 4.9 Å. The cycloaddition product was partially regenerated by heating at 200 °C in mesitylene for 2 days.

#### 5.2.8 Post-synthesis coordination modification

Transition metal complexes that cannot be incorporated pre-synthetically are possible to be incorporated into COF *via* post-SM. As compared to pre-synthesis coordination modification, the opportunities for post-synthesis coordination modification are much more plentiful. Metal coordination sites can either be a binding site on the monomer or linkages of COFs.^351^

**Fig. 63:**
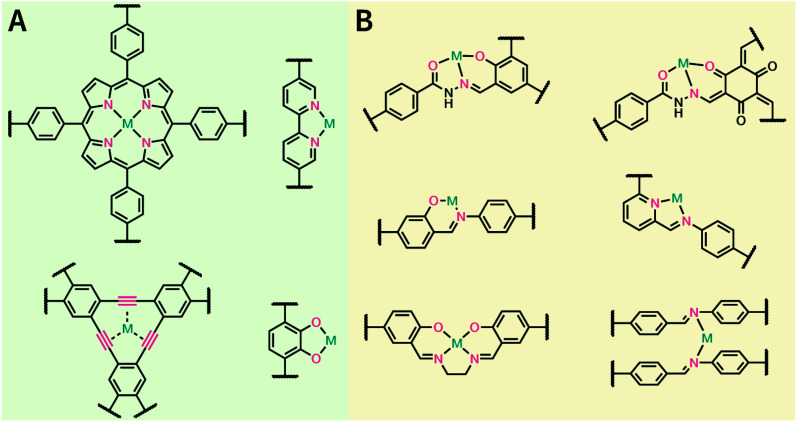
Representative examples of the post-synthesis coordination modification of COFs. (A) Metal coordination sites on the monomer. (B) Metal coordination sites on linkages.

At present, monomers containing coordination sites can be categorized into four types (Fig. 63A): porphyrin,^352–355^ 2,2′-bipyridine,^356–359^ catechol,^360,361^ and 5,6,11,12,17,18-dehexahydrotribenzo[*a*,*e*,*i*][12]annulene.^362^ In general, COF metallization can be readily achieved by simply mixing COFs with metal salts or metal complexes to trigger metal coordination reactions or ligand exchange reactions. Therefore, even metal carbonyl complexes with poor stability can be fixed in the COF pores.^357^

On the other hand, even though there are no chelating coordination sites on the monomers, COF metallization can be performed *via* linkages as the ligands (Fig. 63B), particularly for hydrazine-based linkages with multiple heteroatoms.^363,364^ For imine-linked COFs, only N atoms in the linkage can participate in the coordination process. However, the interlayer distance of ∼3–4 Å of COFs makes the N atoms in the linkages come close to each other, which facilitates the insertion of metal ions between the layers and promotes the formation of coordination bonds.^64,356^ In addition, it is beneficial for imine-linked COFs to fix metal ions by introducing hydroxyl groups,^365–367^ carboxyl groups,^368^ and pyridine^369^ to the monomers, since additional coordination atoms are provided. For example, NiCl_2_ and hydroxyl-containing RIO-12 COF were refluxed in ethanol under basic conditions, and Ni^2+^ was coordinated to the *N*-salicylideneaniline unit in RIO-12 to form a catalyst for the Suzuki–Miyaura cross-coupling reaction.^365^ Zhu *et al.* simply mixed the COFs containing the carboxyl group with the metal chloride solution to fix Ca^2+^, Mn^2+^, and Sr^2+^ on the framework, enhancing the ammonia uptake.^368^ Besides, *N*,*N*′-bis(salicylidene)ethylenediamine (Salen) is an attractive structural unit for coordination chemistry due to its stable planar square structure after chelating metal ions.^370–372^

The post-synthesis coordination modification of COFs provides valuable opportunities to deposit metal or metal oxide nanoparticles in the COF pores. COFs coordinated with metal ions (M^*n*+^@COF) can be further reduced or hydrolyzed to produce M@COF or M_*x*_O_*y*_@COF composite materials, which have been widely studied for a variety of heterogeneous catalytic applications.^373–377^

#### 5.2.9 Post-synthesis ion exchange

For ionic COFs with charges on the monomer, their counter ions can be exchanged by other isoelectric ions, thereby generating new COF materials. For example, EB-COF:Br contains ethidium cations; therefore, the counteranion Br^−^ can be exchanged by polyoxometalate [PW_12_O_40_]^3−^ to afford EB-COF:[PW_12_O_40_]^3−^, thereby improving the proton conductivity.^378^ If the counteranion Br^−^ was exchanged by [Mo_3_S_13_]^2−^, EB-COF:[Mo_3_S_13_]^2−^ can be obtained for efficient photocatalysis.^379^ Furthermore, another type of imidazolium-based cationic COF, namely, Im-COF-Br, can be used as an all-solid-state electrolyte for lithium-ion batteries by exchanging Br^−^ in the framework with the bis(trifluoromethylsulfonyl)imide (TFSI^−^) ion.^380^

In 2019, Zhang *et al.* reported anionic COFs as single-ion-conducting COF solid electrolyte materials.^381^ The treatment of benzimidazole-containing R-ImCOF (R = –H, –CF_3_, and –CH_3_) with *n*-butyllithium in *n*-hexane deprotonated the imidazole ring to form lithium-imidazolate-containing COFs. The imidazolate anions formed loose ion pairs with lithium cations, leading to high lithium-ion conductivity.

#### 5.2.10 Host–guest encapsulation

Host–guest encapsulation is a versatile and effective modification strategy to endow COFs with more functions, which utilizes accessible and permanent pores of COFs to capture guest molecules, including a variety of functional inorganic molecules,^382–386^ organic molecules,^387–389^ and even biological macromolecules.^390–393^

The structural basis of host–guest encapsulation is the pores of COFs, which have unique characteristics.^394,395^ First, the pores of COFs have a precise polygonal reticular structure with well-defined angles. The pore size can be predesigned from micropores to mesopores, which makes it possible to predesign the COF structures according to the size of the guest molecules to meet different needs. Next, morphologically, the pores of 2D COFs are one-dimensional (1D) pore channels instead of a closed cavity, which allows guest molecules to readily enter the pore from the top or sides. Finally, these 1D pore channels are spatially independent and isolated from each other. Moreover, the pore walls can also be pre-decorated *via* pore surface engineering (including pre-SM and post-SM) to establish a tailored interface to precisely regulate the interactions between the framework and guest molecules, facilitating the regulation of the release of guest molecules under different conditions. In conclusion, the pore shape, size, and chemical environment of COFs can be precisely predicted, which are significantly important structural parameters to control the interactions between COFs and guest molecules.

Host–guest encapsulation is the prerequisite of a COF-based drug delivery system, which will be systematically discussed in the next section. Here, we mainly emphasize upon the encapsulation of biological macromolecules by COFs. Due to the large and regular pores of COFs, the controlled loading and release of enzymes on the COF platform allows the enzyme to remain viable under harsh environmental conditions, which is imperative for enzyme catalysis and undoubtedly expands the application ranges of the enzymes.^392,393^ Meanwhile, the chemical modification of the pore walls of COFs can easily regulate the pore environment, thereby improving the interaction and compatibility of COFs with specific enzymes, as well as optimizing the orientation of the active sites of the enzyme, thereby further improving the catalytic activity of the enzymes.^391^

## 6 Nanotherapeutics applications

If we liken COF-based oncotherapy to bread, we have introduced the ingredients (synthesis and nanocrystallization methods of COFs) and tools (functionalization methods of COFs) for making bread. Here, we will introduce the different kinds of methods to make the bread: COF-based oncotherapy examples. Till now, COFs have been used as drug delivery carriers,^221,396–403^ phototherapy reagents,^163,216,223,261,404–410^ and combination therapy platforms^161,162,220,411–415^ for tumor therapeutics (Table 5).

**Table 5:** Summary of COF-mediated cancer therapeutics. Cell lines: human breast adenocarcinoma cell (MCF-7), human breast adenocarcinoma cell (MDA-MB-231), human cervical cancer cell (HeLa), human colorectal carcinoma cell (HCT-116), human embryonic kidney cell (HEK293), human hepatocellular carcinoma cell (HepG2), human non-tumorigenic breast epithelial cell (MCF-10A), human normal liver cell (HL-7702, L02), human pancreatic carcinoma cell (MIA PaCa-2), human renal cancer cell (786-O), human small lung carcinoma cell (A549), immortalized mouse brain capillary endothelial cell (bEnd.3), immortalized renal tubular epithelial cell (HK-2), mouse fibroblast cell (L929), mouse hepatocellular carcinoma cell (H22), mouse mononuclear macrophage leukemia cell (RAW264.7), murine cervical carcinoma cell (U14), murine mammary carcinoma cell (4T1), murine melanoma cell (B16F10), murine renal cancer cell (Renca), and *Mus musculus* colon carcinoma cell (CT26)

COF-based material	Linkage	Shape and size	Therapeutics	Cell line	Tumor model	Ref.
PI-3-COF, PI-2-COF	Imine	Needle shaped morphology of PI-3-COF; spherical nanoparticle-like morphology of PI-2-COF	Drug delivery of fluorouracil	MCF-7	Not applicable	396
TTI-COF	Imine	Elongated morphology with size of several micrometers	Drug delivery of quercetin	MDA-MB-231, MCF-10A	Not applicable	397
TAPB-DMTP-COF	Imine	Monodispersed COF particles with average size of 200 nm	Drug delivery of doxorubicin	HeLa, L929	H22, intratumor injection	221
TpASH-FA	Hydrazone & β-ketoenamine	Nanosheets with thickness of <20 nm	Targeted drug delivery of fluorouracil	MDA-MB-231	Not applicable	398
PEG_*X*_-CCM@APTES-COF-1	Boroxine	Monodispersed COF particles with size of 150–230 nm	Drug delivery of doxorubicin	HeLa	HeLa, intravenous injection	399
PEG_350_-CCM@APTES-COF-1	Boroxine	Thin-platelet morphology with width of 120–150 nm and thickness of 3–5 nm	Brain-targeted drug delivery of pazopanib	Renca, bEnd.3, 786-O, HK-2	Intracranial orthotopic models of brain metastasis from renal cancer	400
F68@SS-COF	Imine	Spherical particles with size of 140 ± 15 nm	Glutathione-responsive drug delivery of doxorubicin	HepG2	Not applicable	401
EDTFP-1	Imine	Nanofiber-like morphology with diameter of 22–30 nm and length of 200 nm	Cytotoxicity of EDTFP-1 COF itself	HepG2, HCT-116, A549, MIA PaCa-2	Not applicable	402
TrzCOF	Imine	Micron-sized rod composed of flakes with size of 40–50 nm	Cytotoxicity of TrzCOF COF itself	B16F10, HepG2, HCT-116, HEK293	Not applicable	403
LZU-1-BODIPY-2I, LZU-1-BODIPY-2H	Imine	Spherical particles with size of about 110 nm	Type II PDT, green LED	HeLa, MCF-7, MCF-10A, HL-7702	MCF-7, intratumor injection	216
PcS@APTES-COF-1	Boroxine	Nanosheets with thickness of 15 nm	Type II PDT, 660 nm laser	CT26	CT26, intravenous injection	404
TphDha COF	Imine	Nanodots with size of about 3 nm	Type I and type II PDT, 638 nm laser	HeLa, MDA-MB-231, RAW 264.7, L929	H22, intravenous injection	163
TphDha COF	Imine	COF particles with hydrodynamic diameter of about 220 nm	PDT, 633 nm laser	MCF-7, HepG2, MCF-10A	MCF-7, intratumor injection	405
TphDha COF	Imine	Core–shell structure with core diameter of 30 nm and shell thickness of 15–30 nm	Type II PDT, 980 nm laser	HEK293, LO2, HeLa, HepG2	4T1, intratumor injection	261
TPAPC-COF	Imine	Spherical particles with size of <1 μm	Type II PDT, 635 nm laser	MCF-7	Not applicable	406
COF-808, COF-909	Imine	Angular particles with size of 100–150 nm	Type I PDT, 630 nm laser	CT26	CT26, intratumor injection	407
CuSe@LZU-1	Imine	Spherical particles with size of 150 nm	PTT, 808 nm laser	L929, HeLa	H22	223
Ag_2_Se@LZU-1	Imine	Spherical particles with size of 150 nm	PTT, 808 nm laser	L929, HeLa	H22	408
Fe-HCOF	β-Ketoenamine	Flower-like rough spheres with size of 2–4 μm	PTT, 808 nm laser	HeLa	U14	409
Py-BPy^+^˙-COF/PEG	Imine	Spherical particles with size of 90 nm	PTT, 808 nm laser, 1064 nm laser	A549	A549, intravenous injection	410
VONc@COF-Por	Imine	Spherical particles with size of 140 nm	Type II PDT, PTT, red LED, 808 nm laser	MCF-7	MCF-7, intratumor injection	220
NCOF-366	Imine	Spherical particles with size of about 100 nm	Type II PDT, PTT, 635 nm laser	4T1	4T1, intratumor injection	411
TP-Por COF	Boronate ester	Nanosheets with thickness of 25 nm	Type I PDT, PTT, 635 nm laser	HeLa, 4T1	HeLa, intratumor injection	162
COF@IR783@CAD	Boronate ester	Nanosheets with size of 200 nm and thickness of 9.5 nm	Drug delivery of *cis*-aconityldoxorubicin, PTT, 808 nm laser	4T1	4T1, intravenous injection	161
PFD@COF_TTA-DHTA_@PLGA–PEG, NM-PPIX	Imine	Spherical particles with size of about 60 nm	Drug delivery of pirfenidone, PDT, 660 nm	CT26	CT26, intravenous injection	412
ICG@COF-1@PDA	Boroxine	Nanosheets with size of 170 nm and thickness of 5.4 nm	Photoimmunotherapy, 808 nm	CT26	CT26, 4T1, intravenous injection	413
COF@ICG@OVA	Imine	Monodispersed spherical particles with size of approximately 100 nm	Immune checkpoint inhibitor treatment, PDT, PTT, 650 nm, 808 nm	CT26, L929	H22, intravenous injection	414
CaCO_3_@COF-BODIPY-2I@GAG	Imine	Spherical particles with hydrodynamic diameter of 319.4 nm	Ca^2+^ overload, PDT, green LED	HCT-116, MCF-7, L-02	HCT-116, MCF-7, intratumor injection	415

### 6.1 Drug delivery and chemotherapy

#### 6.1.1 Principle of chemotherapeutic drug delivery

Chemotherapy is one of the dominant means for clinical treatment of cancer.^416,417^ However, the efficacy of many chemotherapeutic drugs is limited by many factors. (i) Low solubility: to some extent, the low solubility of a large number of chemotherapeutic drugs (*e.g.*, paclitaxel) in water has led to insufficient bioavailability, forcing larger injectable doses to be given to keep the drug concentration in the lesion within the therapeutic range, which undoubtedly increases off-target toxicity. (ii) Low chemical stability: the photostability of platinum-based anticancer drugs is poor. For example, cisplatin should be strictly protected from light during injection, but photohydration and photoredox reactions cannot be completely avoided.^418^ The gradual deepening of the yellow color of a cisplatin solution during intravenous infusion is still an unavoidable phenomenon. Again, this reduces the concentration of active ingredients, and the photoreactive product has even higher side-effects than cisplatin itself. (iii) Non-selectivity: the *in vivo* distribution of conventional chemotherapeutic drugs is not tumor-selective. The toxicity of chemotherapeutic drugs to healthy cells has been criticized, *e.g.*, the cardiotoxicity of anthracyclines such as doxorubicin (DOX)^419,420^ and the toxicity of platinum-based anticancer drugs (*e.g.*, cisplatin) toward the renal and digestive systems.^421,422^ (iv) Drug resistance: the proportion of drug-resistant cells that overexpress efflux transporter proteins increases during treatment, and cancer chemotherapy often struggles to achieve the desired results.^423^

At the level of tissues and organs, nanopharmaceuticals have overcome the challenges faced by conventional chemotherapy drugs to some extent. Among them, the most important one is the biodistribution characteristics exhibited by nanoparticles during *in vivo* circulation, which can be divided into three parts: phagocytosis escape, passive targeting, and active targeting.

First, nanoparticles with surface functionalization can be prevented from being recognized and eliminated by the mononuclear phagocytic system (MPS) and reticuloendothelial system (RES), which increases the circulation time of the active ingredient and therefore extends the drug half-life.^424–426^

**Fig. 64:**
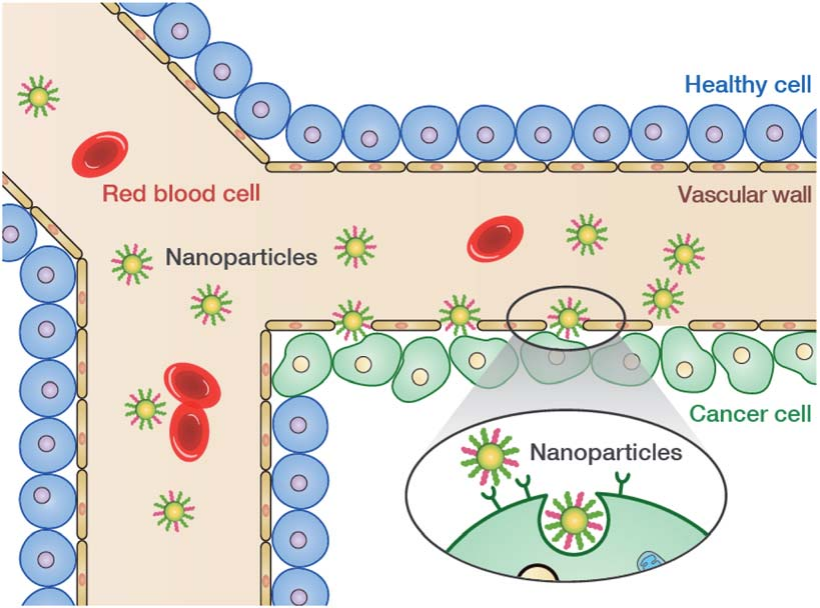
Schematic diagram of the EPR effect (passive targeting) of nanomedicine.

Second, nanoparticles have an enhanced permeability and retention (EPR) effect^427^ that increases the amount of accumulation at the tumor site, which is called passive targeting (Fig. 64). Although passive targeting is not yet described as selective and specific accumulation, it is a considerable improvement over small-molecule drugs. A common explanation for the EPR effect is that tumor cells secrete angiogenesis-associated growth factors, such as vascular endothelial growth factor in response to the rapid growth requirements that are highly dependent on tumor vessels for nutrients and oxygen. At this point, the newly generated tumor vessels are completely different in structure and morphology from normal blood vessels: large endothelial cell gap, missing smooth muscle layer of vessel wall, and deficient angiotensin receptor function. In addition, the lack of lymphatic vessels in the tumor tissue results in lymphatic obstruction. Both situations allow the nanoparticles to accumulate in the tumor tissue and remain in the tumor tissue for a long time without being carried away by the lymphatic circulation. More importantly, the EPR effect can be further enhanced by some pathological and physiological factors, such as bradykinin, nitric oxide (NO), peroxynitrite (ONOO^−^), prostaglandin, and tumor necrosis factor, which stimulate tumor vasodilation.

Finally, in order to further enhance the enrichment of nanoparticles at the tumor site, they can also be functionalized with targeting groups,^428^ aptamers,^429,430^ and antibodies^431^ to promote their interaction with the tumor cells and overexpress receptors in the extracellular matrix, which is called active targeting. However, latest research has shown that the endothelial pathway and trans-cell transportation pathway are far more important than expected for the enrichment of nanoparticles in tumor tissues.^432–434^

At the cellular and molecular levels, nanoparticles are taken up by the cells almost indiscriminately *via* endocytosis, thereby bypassing the limitations of the selective permeability of the cell membrane toward small-molecule drugs.^435^ However, after uptake by cells, nanoparticles get restricted to acidic organelles such as endosomes and lysosomes and are gradually degraded, which limits their subsequent functionalities.^436^ Fortunately, for NCOFs, particularly imide-linked COF, the N atoms in the linkage are alkaline,^437^ causing an increase in the endo-/lysosomal pH, which induces the formation of Cl^−^ ions and water molecules into the endo-/lysosome. When exceeding the endo-/lysosomal self-adjusting ability, endo-/lysosomes swell osmotically and eventually lead to endo-/lysosomal rupture, which release NCOFs into the cytoplasm. This process is known as endo-/lysosomal escape facilitated by the proton sponge effect.^438^ In addition, some nanoparticles with subcellular targeting properties can precisely act on specific organelles to achieve subcellular-targeting therapy.^439,440^ This is beneficial for minimizing drug doses, improving therapeutic benefits, and reducing side-effects.

#### 6.1.2 Model research on drug adsorption and release

As mentioned in the earlier section, the structural basis of COF-related drug delivery is the availability of tunable permanent pores. The loading and release kinetics of small-molecule drugs based on host–guest systems have been extensively studied.

**Fig. 65:**
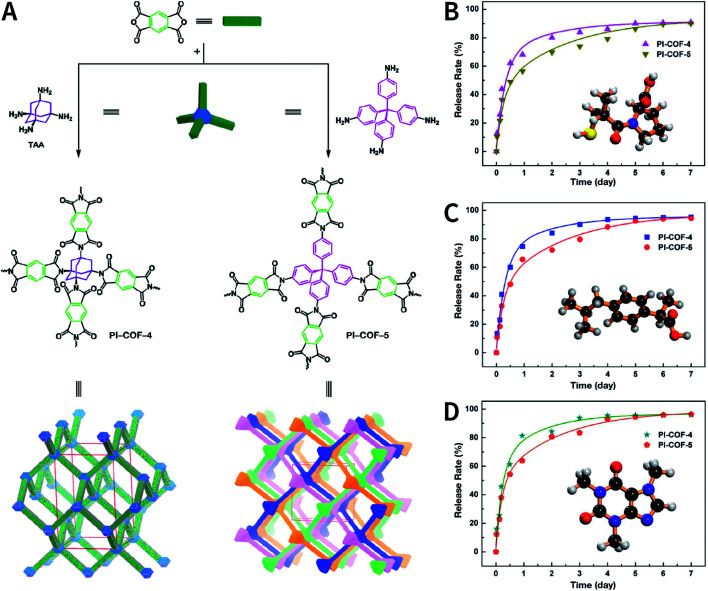
3D polyimide COFs for drug loading and release. (A) Preparing 3D PI-COF-4 and PI-COF-5. (B) Release profile of captopril-loaded 3D COFs. (C) Release profile of ibuprofen-loaded 3D COFs. (D) Release profile of caffeine-loaded 3D COFs. Adapted with permission.^97^ Copyright 2015, American Chemical Society.

In a pioneering study, Yan *et al.* prepared 3D PI-COF-4 and PI-COF-5 (Fig. 65A) by the solvothermal reaction of linear pyromellitic dianhydride with two tetrahedral forms of tetraamines (adamantane-1,3,5,7-tetraamine and 4,4′,4′′,4′′′-methanetetrayltetraaniline) in *N*-methyl-2-pyrrolidone/mesitylene/isoquinoline (10 : 50 : 1, v/v/v).^97^ PI-COF-4 and PI-COF-5 have non- and fourfold-interpenetrated diamond nets, respectively, and their nitrogen adsorption isotherm analyses reveal that their BET specific surface areas are 2403 and 1876 m^2^ g^−1^ with pore sizes of 13 and 10 Å, respectively. Due to their huge specific surface areas, PI-COF-4 and PI-COF-5 can effectively load captopril, ibuprofen, and caffeine model drugs. For example, PI-COF-4 and PI-COF-5 were stirred in ibuprofen *n*-hexane solution for 2 h, and the loading amounts were up to 24 and 20 wt%, respectively. Further, for the drug release process, the release rates of ibuprofen in PI-COF-4 and PI-COF-5 were 60% and 49% at 12 h, respectively, indicating that the drug release is directly related to the pore size and geometry of COFs. For both PI-COF-4 and PI-COF-5, after 6 days, over 95% of the drug molecules were released (Fig. 65B–D).

The adsorption and release behaviors of ibuprofen in other COFs have been extensively studied. Salonen *et al.* found that the equilibrium adsorption capacity of fluorine-containing TpBD-(CF_3_)_2_ COF for ibuprofen in water was 119 mg g^−1^, while the equilibrium adsorption capacity for the more hydrophilic acetaminophen and ampicillin was less than 20 mg g^−1^.^441^ Besides, MICOF@SiO_2_ core–shell material not only exhibited superior adsorption capacity toward ibuprofen, but also showed excellent adsorption capacity for other nonsteroidal antiinflammatory drugs such as ketoprofen, diclofenac, indomethacin, flurbiprofen, and naproxen.^254^ The adsorption of other model molecules in COFs has also been studied, such as sulfamerazine^389^ and Congo red.^442^ These results provide valuable information for understanding the interactions between small molecules and COF pores and for formulating relationships among the molecular structure, hydrophilicity, molecular configuration, and adsorption and desorption behaviors of the molecules in the COF pores.

#### 6.1.3 COF-based drug delivery *in vitro* and *in vivo*

**Fig. 66:**
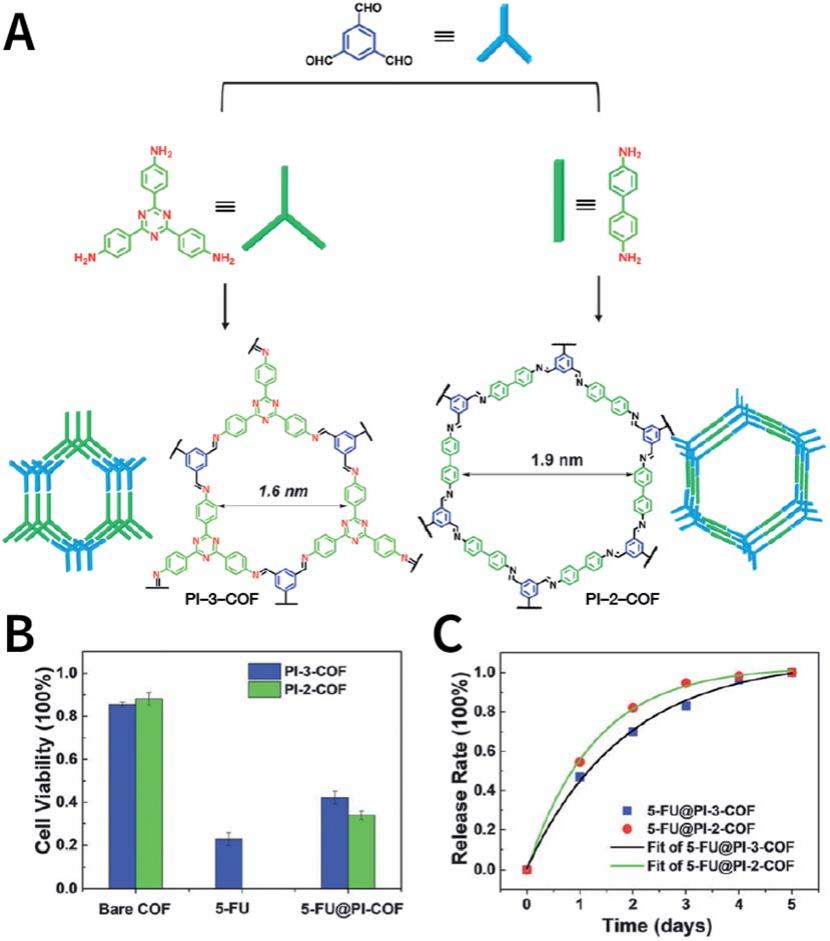
PI-3-COF and PI-2-COF as smart carriers for 5-FU drug delivery. (A) Syntheses and topologies of PI-3-COF and PI-2-COF. (B) Effect of bare COFs, 5-FU, and 5-FU-loaded COFs on cell viability. (C) Drug release profiles of 5-FU-loaded COFs at 100 mg mL^−1^. Adapted with permission.^396^ Copyright 2016, The Royal Society of Chemistry.

In 2016, Zhao *et al.* investigated the potential of imine-linked PI-3-COF and PI-2-COF (Fig. 66A) as drug delivery vehicles and their cytotoxicities *in vitro*.^396^ The antitumor drug fluorouracil (5-FU) was stirred with PI-3-COF and PI-2-COF in *n*-hexane for drug loading. As shown in Fig. 66B, cell inhibition experiments confirmed that PI-3-COF and PI-2-COF had good biocompatibility and no significant inhibition on the proliferation of MCF-7 cells. However, 5-FU@PI-3-COF and 5-FU@PI-2-COF had obvious toxicity toward MCF-7 cells. After a total of 24 h of coincubation, the cell viability was reduced to about 40%. Although the cytotoxicity of 5-FU small molecules was stronger than that of 5-FU@PI-3-COF and 5-FU@PI-2-COF materials within 24 h, the COFs provided up to several days of continuous drug release ability, as per the drug release curve (Fig. 66C). This slow-release feature was not available in small-molecule drugs.

**Fig. 67:**
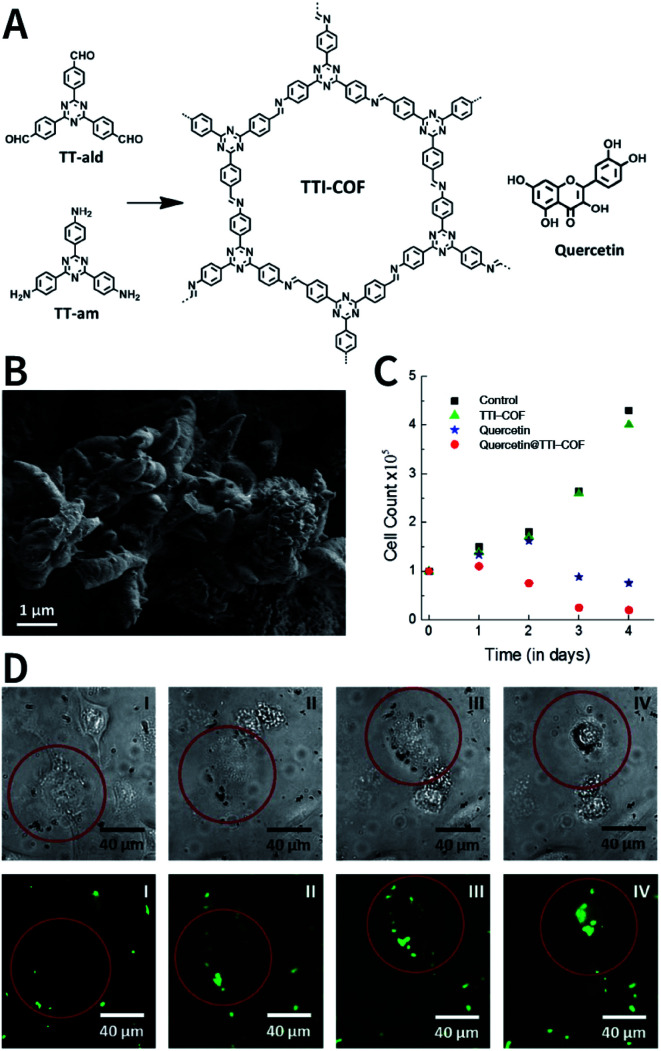
Imine-linked TTI-COF as a drug delivery vehicle for quercetin. (A) Structures of TTI-COF and quercetin. (B) SEM image of TTI-COF. (C) Effect of TTI-COF, quercetin, and quercetin@TTI-COF materials on the number of cells during cell culture. (D) Cell uptake and apoptosis during co-culture with quercetin@TTI-COF. Adapted with permission.^397^ Copyright 2016, Wiley-VCH Verlag GmbH & Co. KGaA, Weinheim.

TTI-COF^397^ was prepared by the imine condensation of 4,4′,4′′-(1,3,5-triazine-2,4,6-triyl)tribenzaldehyde and 4,4′,4′′-(1,3,5-triazine-2,4,6-triyl)trianiline, where the electron pairs on imine nitrogen reversibly anchored the quercetin guest molecules through noncovalent interactions (Fig. 67). Interestingly, although the SEM image of TTI-COF showed that the particle diameter of TTI-COF was even greater than 1 μm, quercetin@TTI-COF could efficiently deliver quercetin to the MDA-MB-231 cells, leading to cell apoptosis. COF-based quercetin delivery was not restricted by Bcrp1 overexpression, which is thought to be the primary cause that limits the cellular uptake of quercetin. Quercetin was unable to obtain its drug release profile due to its susceptibility to oxidation, but quercetin@TTI-COF consistently inhibited cell proliferation over the course of 4 day cell culture and was superior to quercetin small molecules with regard to cell inhibition, indicating slow drug release. In addition, the negligible effect of TTI-COF on cell proliferation was observed, suggesting the biocompatibility of TTI-COF.

In addition to using the principle of host–object envelopment to load drugs into COFs *via* post-SM, Pang *et al.* demonstrated that DOX, a chemotherapeutic drug, could be directly loaded into the pores during COF formation *via* an *in situ* one-pot method.^221^ Briefly, DOX and 2,5-dimethoxyterephthalaldehyde were stirred for 1 h and then 1,3,5-tris(4-aminophenyl)benzene was added to form TAPB-DMTP-COF. In this way, the drug loading of DOX was as high as 32.1 wt%. For *in vitro* experiments, DOX@TAPB-DMTP-COF was effectively taken in by HeLa cells, and it inhibited cell proliferation. For *in vivo* experiments in a xenograft model of H22 cells, an intratumoral injection of DOX@TAPB-DMTP-COF also showed significant tumor-suppressive effects. Notably, the authors believed that the reaction of the amino in DOX with 2,5-dimethoxyterephthalaldehyde resulted in a shift in the main diffraction peak of TAPB-DMTP-COF, possibly suggesting an alteration in the structure of COFs that requires further study.

The above examples demonstrate the feasibility of using COFs as drug delivery vehicles *in vitro* and *in vivo*. However, due to the low dispersibility and inadequate bioavailability of bulk COFs, it may encounter serious defects such as premature clearance and ambiguous targeting when used for intravenous injections. As mentioned earlier, the surface modification of functional ingredients to ameliorate the deficiency of bulk COFs becomes a possible solution.

**Fig. 68:**
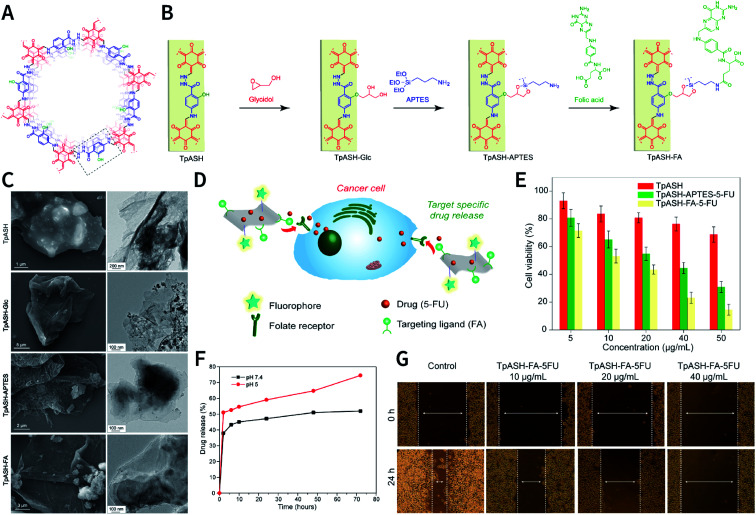
Targeted drug delivery *via* sequential post-SM of COFs. (A) Structure of TpASH COF. (B) Sequential post-SM of TpASH COF to generate FA-coupled TpASH-FA nanosheets. (C) SEM and TEM images of TpASH, TpASH-Glc, TpASH-APTES, and TpASH-FA. (D) COF nanosheets labeled with the targeting group and fluorescent dye for targeted drug delivery. (E) Inhibitory effect of the 5-FU-loaded COF nanosheets on MDA-MB-231 cancer cells. (F) Drug release curves of TpASH-FA-5-FU at different pH values. (G) Evaluation of the inhibitory effect of TpASH-FA-5-FU on cell migration using cell scratch tests. Adapted with permission.^398^ Copyright 2017, American Chemical Society.

Due to the strong chemical stability and abundant functionalization potential of COFs, it is feasible to use multistep post-SM for functionalization. The first report in this regard discussed the preparation of folic acid (FA)-coupled TpASH-FA COF nanosheets for 5-FU-targeted drug delivery *in vitro* (Fig. 68) by the three-step post-SM of TpASH COF.^398^ TpASH COF was prepared by mechanically grinding 2,4,6-trihydroxybenzene-1,3,5-tricarbaldehyde and 4-amino-2-hydroxybenzohydrazide as the monomers and 4-methylbenzenesulfonic acid as the catalyst. The hydroxyl groups on the TpASH COF monomer provided the reaction sites for post-SM. In the three-step post-SM processes, the first step comprised the epoxide ring-opening reaction of glycidol to convert phenolic hydroxyl groups into alcoholic hydroxyl groups to yield TpASH-Glc; the second step involved the conversion of these surface alcoholic hydroxyl groups into amines in the presence of 3-(triethoxysilyl)propan-1-amine (APTES) to afford the amine-functionalized TpASH-APTES. Finally, the amino group underwent a condensation reaction with FA to produce TpASH-FA as the target product. Besides, the amino groups on the surface of TpASH-APTES can also conjugate with the fluorescent dye of rhodamine-B-isothiocyanate (RITC) to perform fluorescent labeling. It should be noted that continuous post-SM led to the weakening of the π–π stacking between the COF layers, resulting in exfoliation and enhanced water dispersion of the COFs. Drug loading was achieved by simply stirring COF and 5-FU in water. When the concentration of TpASH-FA-5-FU was 50 μg mL^−1^, the cell viability of MDA-MB-231 was reduced to 14%, while the cell viability of nontargeted TpASH-APTES-5-FU was approximately 30%. A mechanistic study confirmed that the specific targeting effect of FA on MDA-MB-231 tumor cells enhanced the cell uptake, thereby increasing cell death. In addition, TpASH-FA-5-FU exhibited an inhibitory effect on cell migration.

**Fig. 69:**
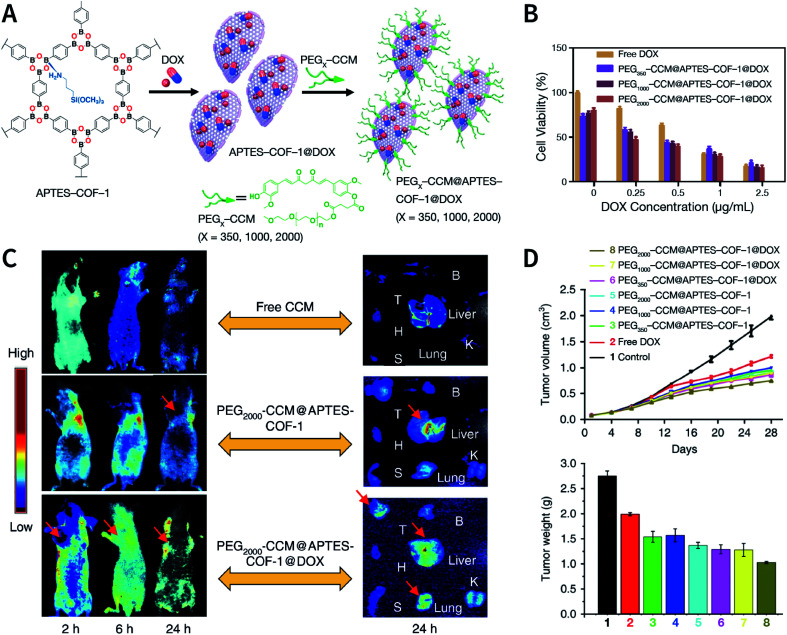
Water-dispersible PEG-curcumin-coated COFs for *in vivo* DOX drug delivery. (A) Synthesis of PEG_*X*_-CCM@APTES-COF-1@DOX (*X* = 350, 1000, and 2000). (B) *In vitro* antitumor experiment. (C) *In vitro* and *ex vivo* fluorescence images of mice after intravenous injections of nanodrugs over a period of 24 h. (D) *In vivo* antitumor experiment. Adapted under a Creative Commons Attribution 4.0 International License. Copyright 2018, The Author(s).^399^ Published by Springer Nature Limited.

Besides targeting groups, polyethylene glycol (PEG) derivatives have been modified onto the COF surface to enhance hydrophilicity and tumor accumulation. As shown in Fig. 69A, by the self-assembly of curcumin (CCM)-modified PEG and amine-functionalized APTES-COF-1@DOX, Jia *et al.* prepared a series of water-dispersible PEGylated COF nanodrugs of PEG_*X*_-CCM@APTES-COF-1@DOX (*X* = 350, 1000, and 2000). A PEG_*X*_-CCM coating not only imparted fluorescence imaging capabilities to the nanodrug, but also significantly improved the drug loading and release kinetics, cell uptake, blood circulation time, and tumor accumulation capacity. The DOX content in PEG_2000_-CCM@APTES-COF-1@DOX was 9.71 ± 0.13 wt% and the encapsulation efficiency was as high as 90.5 ± 4.1%. The *in vitro* experiments indicated that PEG_*X*_-CCM@APTES-COF-1@DOX was broken down in lysosomes, resulting in slow DOX release. Even at very low DOX concentrations (0.25 μg mL^−1^), PEG_*X*_-CCM@APTES-COF-1@DOX significantly inhibited cell proliferation (Fig. 69B). The *in vivo* fluorescence imaging on mice showed that the nanodrugs were mainly distributed in tumor tissues after the injection of nanodrugs into the tumor-bearing mice *via* the tail vein for 24 h. Among them, PEG_2000_-CCM@APTES-COF-1@DOX exhibited the best tumor-targeting ability (Fig. 69C). These results were consistent with those of *in vivo* antitumor experiments (Fig. 69D).

Furthermore, the latest research confirms that PEG_350_-CCM@APTES-COF-1@pazopanib could penetrate the blood–brain barrier of mice and achieve intracranial tumor accumulation in the orthotopic models of brain metastasis from renal cancer.^400^ Due to the brain-targeting characteristic, as compared to the direct drug administration of pazopanib, PEG_350_-CCM@APTES-COF-1@pazopanib nanomedicine more significantly inhibited angiogenesis and tumor growth, protected mice from systemic drug toxicity, and prolonged survival time.

In addition to PEG, Pluronic F68 can also achieve a similar result. For example, Zhang *et al.* prepared Pluronic F68-modified F68@SS-COF for DOX drug delivery.^401^ SS-COF was prepared by the imine condensation reaction between benzene-1,3,5-tricarbaldehyde and 4,4′-disulfanediyldianiline. The disulfide bond enabled F68@SS-COF to decompose in the presence of glutathione, thereby releasing the encapsulated DOX. DOX-loaded F68@SS-COF was confirmed to have a significant inhibitory effect on HepG2 cells.

In most COF-based drug delivery systems, COFs themselves have been reported to be nontoxic or less toxic. However, thus far, two exceptional cases have been reported by Bhaumik and co-workers. EDTFP-1,^402^ obtained by the reflux of 2,4,6-trihydroxybenzene-1,3,5-tricarbaldehyde and 4,4′-(ethane-1,2-diyl)dianiline at 150 °C in *N*,*N*-dimethylformamide for 12 h, exhibited fibrous morphology with a length of about 200 nm and diameter of ∼22–30 nm, as confirmed by its SEM images. The half-maximal inhibitory concentrations (IC_50_) of EDTFP-1 on HCT-116, A549, HepG2, and MIA PaCa-2 cells were 9.89 ± 1.16, 11.88 ± 1.82, 14.38 ± 2.01, and 14.30 ± 1.32 μg mL^−1^, respectively. These data indicate that the toxicity of EDTFP-1 is comparable to that of 5-FU, a small-molecule broad-spectrum antitumor drug. In 2018, Bhaumik *et al.* further found that TrzCOF, with 5′′-(4′-formyl-[1,1′-biphenyl]-4-yl)-[1,1′:4′,1′′:3′′,1′′′:4′′′,1′′′′-quinquephenyl]-4,4′′′′-dicarbaldehyde and 4,4′,4′′-(1,3,5-triazine-2,4,6-triyl)trianiline as the monomers, exhibited similar cytotoxicity.^403^ The IC_50_ values of TrzCOF on B16F10, HepG2, HCT-116, and HEK293 cells were 10.47 ± 2.63, 9.55 ± 2.13, 8.31 ± 1.67, and 14.39 ± 2.25 μg mL^−1^, respectively. At the same concentration, the monomers constituting EDTFP-1 and TrzCOF did not show any activity to induce cell death. Although some mechanistic studies have shown that EDTFP-1 and TrzCOF can lead to cell apoptosis *via* a mitochondrial-dependent pathway, the relationship between toxicity and COF structure is not fully understood. In addition, these studies implied that COFs with certain specific structures may have inherent cytotoxicity. Therefore, it is necessary to evaluate their systemic toxicity and biocompatibility of COFs prior to their applications as drug carriers. In addition, it is regrettable that the authors did not mention the toxicity of COFs to normal cells, which is worthy of more attention.

### 6.2 PDT

#### 6.2.1 Principle of PDT

**Fig. 70:**
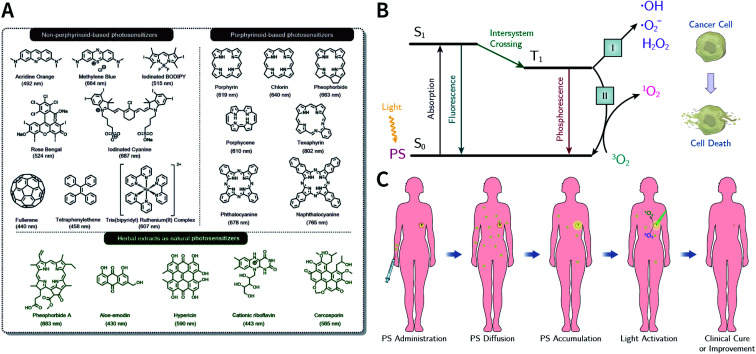
(A) Representative instances of PSs, including non-porphyrinoid-based PSs, porphyrinoid-based PSs, and natural PSs extracted from herbs. Adapted under the CC BY license. Copyright 2020, The Author(s).^14^ Published by Elsevier Ltd. (B) Simplified Jablonski diagram of the PDT mechanisms (type I and type II). (C) Schematic diagram of PDT procedures.

PDT is a novel alternative method for cancer treatment that has shown superior potential for the minimally invasive treatments of various types of cancers, particularly superficial cancers.^443,444^ As shown in Fig. 70A, PDT relies on a nontoxic photosensitizer (PS), *e.g.*, porphyrin,^445^ phthalocyanine,^446^ BODIPY,^447^ and cyanine,^448^ to induce the formation of reactive oxygen species (ROS) under specific wavelengths of light, leading to cytotoxicity.^449^ According to the photochemical mechanism of ROS generation (Fig. 70B), PDT can be divided into type I and type II mechanisms.^450,451^ For the type I mechanism, the triplet excited PS interacts with biological substrates to generate free radicals by transferring electrons. These free radicals subsequently react with oxygen or water to form ROS such as hydrogen peroxide (H_2_O_2_), hydroxyl radical (˙OH), and superoxide anion (˙O_2_^−^). For the type II mechanism, the triplet excited PS directly transfers energy to oxygen to form singlet oxygen (^1^O_2_), which is considered to be the more common ROS in most cases.^452^

The general procedures of PDT^453^ are shown in Fig. 70C. The main advantages of PDT are as follows. (i) Since PS has no obvious toxicity under dark conditions, PDT is highly selective through local light and can kill tumor cells without damaging healthy organs. (ii) The toxicity of ROS to tumor cells is universal and no resistance has been observed; therefore, it can be treated multiple times at low doses to minimize adverse effects. (iii) PDT is a minimally invasive therapy option; even for visceral tumors, the required light can be directed to the affected area *via* fiber optics and other means.^454^ (iv) PDT can be easily combined with other treatments, such as chemotherapy and radiotherapy.

Similar to drug delivery, the improvement effect of nanotechnology on PDT can be mainly reflected in the optimization of tumor accumulation.^455^ Traditional small-molecule PSs are usually organic molecules with wide-range conjugated systems. They are poorly water-soluble and can aggregate easily. After systemic administration, the PS has insufficient accumulation in tumor tissues, making it difficult to meet *in vivo* applications.^456^ Nano-PSs make up for the abovementioned shortcomings *via* the EPR effect and active targeting abilities. On the other hand, improvements in the photochemical properties of PSs by nanotechnology cannot be ignored. The uniform modification of PSs on the nanoparticles prevents PS aggregation at the molecular level, thereby preventing fluorescence quenching and improving the ^1^O_2_ quantum yield.^457^

#### 6.2.2 COFs for PDT

**Fig. 71:**
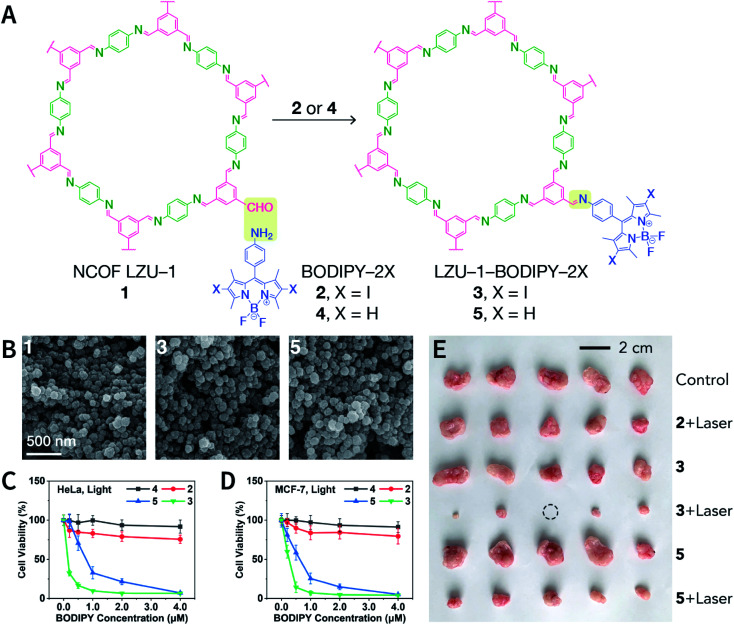
BODIPY-decorated LZU-1 NCOF for PDT. (A) BDF of LZU-1 to yield BODIPY-decorated NCOFs. (B) SEM images of LZU-1, LZU-1-BODIPY-2I, and LZU-1-BODIPY-2H. (C) Phototoxicity toward HeLa cells. (D) Phototoxicity toward MCF-7 cells. (E) Photographs of the tumor tissue obtained after performing *in vivo* PDT. Adapted under the CC BY license. Copyright 2019, The Author(s).^216^ Published by Elsevier Inc.

In 2019, COFs were used for cancer PDT for the first time.^216^ Dong *et al.* used the BDF method to modify two amino-substituted BODIPY PSs on the surface of LZU-1 *via* imine condensation, and they successfully prepared LZU-1-BODIPY-2I containing an iodine atom and LZU-1-BODIPY-2H without an iodine atom (Fig. 71), where the BODIPY contents were 0.136 and 0.155 mmol g^−1^, respectively. SEM images showed that LZU-1, LZU-1-BODIPY-2I, and LZU-1-BODIPY-2H had a uniform size of about 110 nm. Due to the enhanced effect of iodine atoms on intersystem crossing (ISC), LZU-1-BODIPY-2I afforded higher ^1^O_2_ generation efficiency than that by LZU-1-BODIPY-2H. For *in vitro* PDT, under green LED irradiation, when the BODIPY concentration was 0.5 μM, LZU-1-BODIPY-2I almost completely killed HeLa and MCF-7 cancer cells, while LZU-1-BODIPY-2H-induced cell viability was still higher than 50%. The same trend was observed in the MCF-7 xenograft model, suggesting the significant value of the heavy-atom effect at the animal level. Mechanistic studies confirmed that LZU-1-BODIPY-2I entered the cancer cells primarily *via* the energy-dependent endocytosis pathway; then, it was mainly localized at the lysosomes and mitochondria and induced cell death by enhancing the lysosomal membrane permeabilization (LMP) and inducing loss of mitochondrial membrane potential (MMP).

It is also feasible to use the π–π interactions between COFs and PS to adsorb PSs onto the COF surface to prepare COF-based nano-PS. Yuan *et al.* used APTES-COF-1 nanosheets to adsorb phthalocyanine PS to prepare PcS@COF-1.^404^ Because phthalocyanine can be highly dispersed on the surface of APTES-COF-1, PcS@COF-1 exhibits good photodynamic property under laser irradiation at 660 nm, and it can efficaciously reduce the cell viability of CT26 cells to 35% when the phthalocyanine concentration is only 3 μg mL^−1^. The PDT therapeutic effect of PcS@COF-1 has also been confirmed in CT26-tumor-bearing mice.

**Fig. 72:**
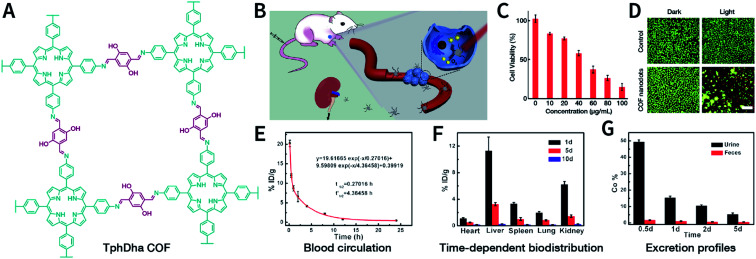
Renal-clearable ultrasmall DSPE-PEG-coated TphDha COF nanodots for PDT. (A) Structure of TphDha COFs. (B) Schematic illustration of COF nanodots for PDT. (C) Cell viability of HeLa cells treated with COF nanodots under 638 nm irradiation for 5 min. (D) Confocal fluorescence imaging of HeLa cells after different treatments. The green channel represents live cells stained with calcein-AM, and the red channel represents dead cells stained with propidium iodide. Scale bar: 20 μm. (E–G) *In vivo* pharmacokinetic and biodistribution profiles of COF nanodots in healthy mice after intravenous injection. Adapted with permission.^163^ Copyright 2019, Elsevier Ltd.

In addition to post-SM, the modularity advantage of COFs allows PS as the monomer to get directly involved in the construction of COF scaffolds. The photosensitizing properties of some porphyrin-based COFs have been reported;^283,458–460^ however, their PDT applications *in vitro* and *in vivo* are still rare. Until recently, Qu *et al.* synthesized ultrasmall porphyrin-based TphDha COF nanodots (Fig. 72A) with the renal-clearable property for *in vitro* and *in vivo* PDT.^163^ First, 4,4′,4′′,4′′′-(porphyrin-5,10,15,20-tetrayl)tetraaniline and 2,5-dihydroxyterephthalaldehyde were used to synthesize bulk TphDha COF in Pyrex tubes; subsequently, ultrasonic exfoliation, surface modification using 1,2-distearoyl-*sn-glycero*-3-phosphoethanolamine-*N*-[methoxy(polyethylene glycol)] (DSPE-PEG), and filtering separation were performed to obtain DSPE-PEG-coated TphDha COF nanodots (size: ∼3 nm). When the nanodots were irradiated by a laser at 638 nm, the nanodots induced ˙OH and ^1^O_2_ production at the same time *via* the type I and type II mechanisms (Fig. 72B). In the dark, the nanodots exhibited marginal toxicities toward HeLa, MDA-MB-231, RAW 264.7, and L929 cells, even at concentrations of up to 200 μg mL^−1^. However, the nanodots obviously inhibited HeLa cell proliferation in a concentration-dependent manner under laser irradiation at 638 nm for 5 min (Fig. 72C and D). Antitumor experiments conducted in H22-tumor-bearing mice further confirmed the excellent PDT effect of these nanodots. Subsequently, biodistribution and pharmacokinetic assays were performed in healthy mice. After intravenous injection of these nanodots, the blood circulation half-lives of distribution and clearance phases were calculated to be 0.27 and 4.36 h, respectively, based on a two-compartment model of the blood circulation curve (Fig. 72E). Further, the nanodots were mainly distributed in the liver and kidneys of healthy mice. Over time, the nanodot concentration in the main organs gradually decreased (Fig. 72F). Research on excretions confirmed that the nanodots mainly existed in urine rather than feces (Fig. 72G); therefore, it was speculated that the nanodots were excreted through the kidneys, which was closely related to the ultrasmall size of the nanodots. Such easily metabolized and excreted nanodots are meaningful for reducing the long-term toxicity of materials.

**Fig. 73:**
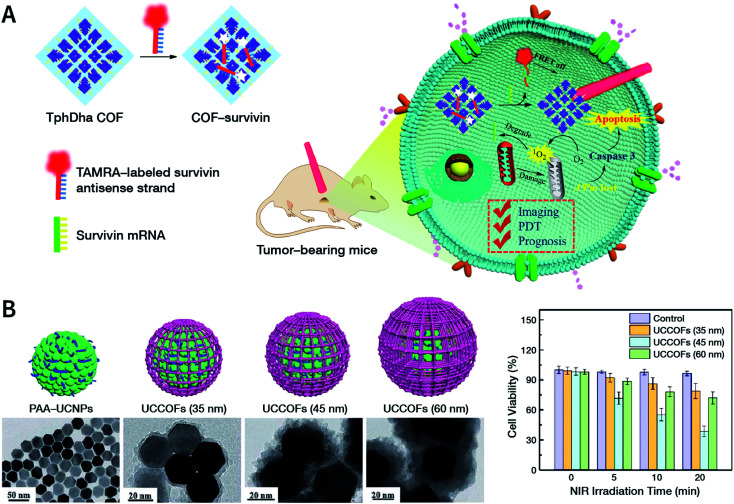
(A) NCOF-based theranostic nanoplatform for cancer diagnosis and PDT by integrating the dye-labeled oligonucleotide onto TphDha COF nanoparticles. Adapted with permission.^405^ Copyright 2020, The Royal Society of Chemistry. (B) UCNP@COF core–shell structure for PDT excited by NIR light at 980 nm. Left: TEM images of PAA-UCNPs, UCCOFs (35 nm), UCCOFs (45 nm), and UCCOFs (60 nm). Right: Cytostatic effect of UCCOFs with different sizes on HeLa cells when excited by NIR laser at 980 nm. Adapted with permission.^261^ Copyright 2020, The Royal Society of Chemistry.

Very recently, Tang and co-workers developed a TphDha-COF-based theranostic nanoplatform (Fig. 73A) by integrating tetramethylrhodamine-labeled survivin antisense strand onto TphDha COF for simultaneous cancer diagnosis and PDT.^405^ The fluorophore was quenched by TphDha COF due to its large plane π-electron system. However, once it penetrated into the cancer cells, in the presence of survivin mRNA as the cancer biomarker, more stable RNA duplexes were formed and divorced from the TphDha COF surface, recovering the fluorescence signal of tetramethylrhodamine and enabling tumor-specific imaging. Furthermore, TphDha COF was irradiated with red light, generating toxic ^1^O_2_ in cancer cells to induce oxidation stress and trigger cell apoptosis through PDT. Therefore, highly tumor-selective PDT became feasible by reasonably combining fluorescence imaging and PDT.

*In situ* growth of TphDha COF on the surface of upconversion nanoparticles (UCNPs) enables PDT excited by near-infrared (NIR) light at 980 nm (Fig. 73B).^261^ Oleic-acid-capped NaYF_4_:Yb,Er,Tm UCNPs (OA-UCNPs) with a diameter of 30 nm were carboxylated by polyacrylic acid (PAA) to obtain PAA-UCNPs. Subsequently, TphDha COF was grown *in situ* under the catalysis of the surface carboxyl group to obtain UCCOFs, and the thickness of the shell was adjusted by changing the reaction conditions. When the UCNP core was excited with a 980 nm laser, the emissions at 541 and 654 nm were absorbed by the TphDha COF shell to produce ^1^O_2_, while the emission at 800 nm was not affected. Further study showed that when the thickness of the shell was 15 nm, the TphDha COF shell gave the best ^1^O_2_ generation capability, leading to the best cytostatic effect of HeLa cells. By loading ^1^O_2_-unstable indocyanine green (ICG) fluorescent dye into the pore of the TphDha COF shell, the *in situ* monitoring of ^1^O_2_ location and therapeutic response could be achieved *in vivo*. This is the first report of COF-based PDT excited by upconversion fluorescence, providing the possibility of deep PDT.

Corrole-based COFs^406,461^ have been successfully synthesized and have shown potential in PDT. Recently, Zhang *et al.* synthesized TPAPC-COF with a distinctive approximately T-shaped 5,10,15-tris(4-aminophenyl)corrole (H_3_TPAPC) molecule and a linear terephthalaldehyde molecule.^406^ TPAPC-COF adopts the staggered AB-stacking form with elliptical pores. The B-band absorption of TPAPC-COF is located at 399 nm, and the extended Q-band absorption is up to 2000 nm, which can be attributed to the huge π-electron delocalization system in the TPAPC-COF layer. When excited by a 635 nm laser, TPAPC-COF exhibited stronger photosensitivity properties than the H_3_TPAPC monomer and induced more efficient ^1^O_2_ production, which may be related to the reduced fluorescence emission and enhanced ISC of TPAPC-COF. For *in vitro* experiments, DSPE-PEG-coated TPAPC-COF effectively inhibited MCF-7 proliferation through PDT.

**Fig. 74:**
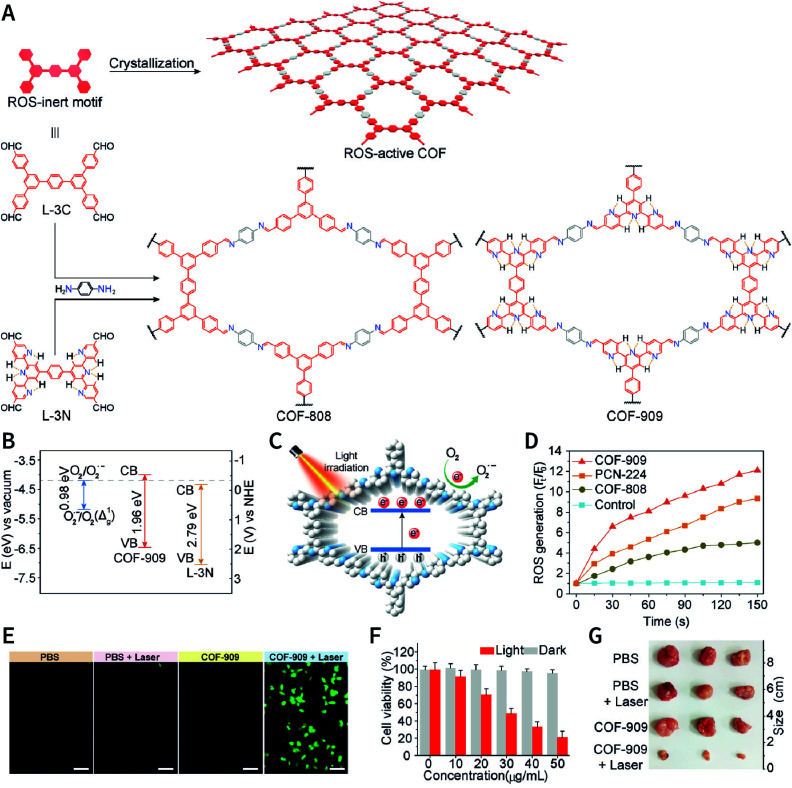
COF-808 and COF-909 for PDT. (A) Syntheses of COF-808 and COF-909 using ROS-inert monomers. (B) Band structures of COF-909 and L-3N. (C) Schematic diagram of the ROS generation mechanism of COF-909 under laser irradiation at 630 nm. (D) ROS generation efficiencies of COF-808, COF-909, and PCN-224, as reflected by fluorescence enhancement. (E) Detection of intracellular ROS mediated by COF-909 under laser irradiation at 630 nm (200 mW cm^−2^). (F) Cytostatic effect of COF-909 on CT26 cells. (G) Tumor images of mice after performing *in vivo* PDT. Adapted with permission.^407^ Copyright 2019, Wiley-VCH Verlag GmbH & Co. KGaA, Weinheim.

All the COF-based PDT systems mentioned above are based on well-known PSs. However, an impressive study showed that COF-Trif-Benz and COF-SDU1 with photodynamic properties could be fabricated from monomers that did not have photosensitive properties at all, although the detailed mechanism has not yet been elucidated.^462^ In 2019, Deng *et al.* synthesized COF-808 and COF-909 with photodynamic properties using inactive 5′,5′′′-bis(4-formylphenyl)-[1,1′:3′,1′′:4′′,1′′′:3′′′,1′′′′-quinquephenyl]-4,4′′′′-dicarbaldehyde (L-3C) and 4′,4′′′′-(1,4-phenylene)bis(([2,2′:6′,2′′-terpyridine]-5,5′′-dicarbaldehyde)) (L-3N) monomers (Fig. 74A).^407^ Although the frontier molecular orbitals of the monomers did not match with those of the superoxide anion, the bandgap of the resulting COFs was precisely narrowed down to provide suitable overlap, which considerably promoted the photodynamic property. In particular, spectroscopic measurements showed that COF-909 had a gap between the frontier orbitals of 1.96 eV and absorbed visible light at 630 nm. However, the bandgap of L-3N was 2.79 eV, making it difficult to excite at the same wavelength (Fig. 74B). When compared with L-3N, COF-909 afforded longer excited state lifetimes, higher separation efficiencies of electrons and holes, and lower charge recombination rates. Therefore, ROS could be easily generated through electron transfer from COF-909 to dissolved oxygen (Fig. 74C). The quantitative determination of ROS showed that the ROS generation ability of COF-909 was even better than that of porphyrin-based MOF PCN-224 (Fig. 74D). At the cellular level, when 630 nm laser was irradiated, COF-909 effectively generated ROS in CT26 cells (Fig. 74E) and caused significant cell death (Fig. 74F). At the animal level, by the intratumoral injection of COF-909 in CT26-tumor-bearing mice, the PDT efficacy was further confirmed (Fig. 74G). Furthermore, in 2020, Qiu *et al.* reported NDA-TN-AO COFs based on non-photosensitive monomers.^463^

### 6.3 PTT

#### 6.3.1 Principle of PTT

PTT is another potential phototherapy method.^12^ It utilizes photothermal agents (PTAs) to convert light energy into heat energy, leading to elevated temperatures at the tumor sites to kill the tumor cells. Because NIR light has a better tissue penetration ability,^464^ the ideal PTA should have high absorption in the NIR region. The remarkable feature of inorganic PTAs,^11,465^*e.g.*, gold nanorods, platinum quantum dots, and graphene nanosheets, is their ability to absorb and manipulate light at the subwavelength scale by supporting coherent electronic oscillation, which is called localized surface plasmon resonance (LSPR).^466^ As energy transfers from light to electron and then from electron to lattice, the lattice transfers the energy to the environment in the form of heat, resulting in the photothermal effect. Furthermore, organic PTAs,^467,468^ such as cyanine and phthalocyanine, have a larger π-conjugated system, which can efficiently absorb NIR light and get excited. When the energy is released through a nonradiative transition, a thermal effect is induced.

#### 6.3.2 COFs for PTT

Till now, COF-based PTT mainly includes two implementation methods. The first one is to combine PTA with COFs and the COF material, in our case, is used as a PTA carrier.^232^ The second is to design and synthesize COFs with light–heat conversion capabilities, *e.g.*, copper(ii) tetraphenylporphyrin-based COFs.^69,469^

In 2019, Pang *et al.* synthesized LZU-1 nanoparticles loaded with CuSe^223^ and Ag_2_Se.^408^ Under an 808 nm laser (1.5 W cm^−2^), the photothermal conversion efficiency of CuS-loaded LZU-1 (200 μg mL^−1^) was 26.3%, while that of Ag_2_Se-loaded LZU-1 (500 μg mL^−1^) was 37.9%. The authors also reported that the photodynamic properties could be attributed to LZU-1, but further cautious confirmation is necessary. Pang *et al.* also prepared micron-sized flower-like HCOF linked by β-ketoenamine.^409^ After metalation with Fe^3+^, the photothermal conversion efficiency of Fe-HCOF (800 μg mL^−1^) was 13.9% under an 808 nm laser (1.9 W cm^−2^).

**Fig. 75:**
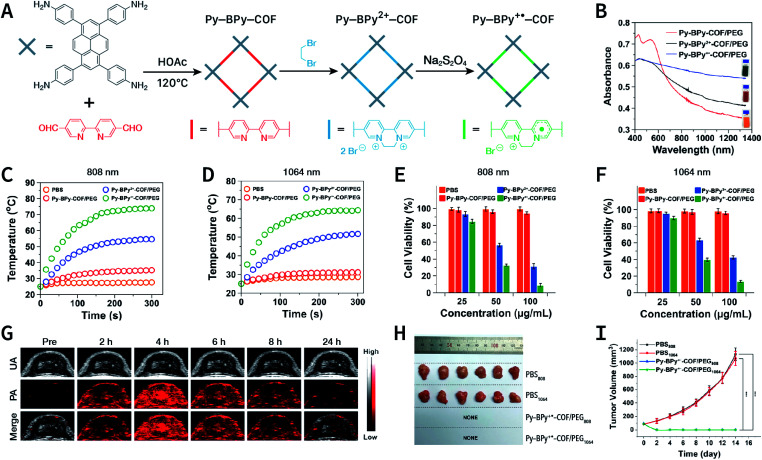
Stable radical cation-containing Py-Bpy^+^˙-COF for PTT. (A) Conversion of Py-BPy-COF to cationic Py-BPy^2+^-COF and cationic radical Py-BPy^+^˙-COF by two-step post-SM. (B) Absorption spectra of Py-BPy-COF/PEG, Py-BPy^2+^-COF/PEG, and Py-BPy^+^˙-COF/PEG at a concentration of 50 μg mL^−1^. (C and D) Photothermal heating curves under laser irradiation at 808 and 1064 nm (1 W cm^−2^) at a concentration of 100 μg mL^−1^. (E and F) A549 cytotoxicity of Py-BPy-COF/PEG, Py-BPy^2+^-COF/PEG, and Py-BPy^+^˙-COF/PEG under laser irradiation at 808 and 1064 nm (1 W cm^−2^). (G) Photoacoustic and ultrasound imaging of the tumor site after an intravenous injection of Py-BPy^+^˙-COF/PEG. (H) Morphology of the tumors at the end of *in vivo* PTT. (I) Tumor growth curves during *in vivo* antitumor therapy. Adapted with permission.^410^ Copyright 2019, American Chemical Society.

Recently, COFs containing free radical cations have been explored for *in vivo* photoacoustic imaging and PTT.^410^ By performing two sequential post-SM processes of quaternization and one-electron reduction, 2,2′-bipyridine-based Py-BPy-COF was converted into cationic-radical-containing Py-Bpy^+^˙-COF (Fig. 75A). Its AA-stacking structure enabled the overlap of redox centers with each other in the COF layers, thereby promoting intercharge transfer through π-coupling multilayers and eventually inducing enhanced NIR absorption and significant photothermal conversion by promoting nonradiative transitions. The absorption spectrum data showed that PEG-functionalized Py-BPy^+^˙-COF dispersion exhibited a broad featureless absorption band in the range of ∼600–1300 nm, where the absorbance was remarkably higher than those of Py-BPy-COF/PEG and Py-BPy^2+^-COF/PEG under the same concentration (Fig. 75B). Under laser irradiation at 808 nm, the photothermal conversion efficiencies were 19.3, 47.2, and 63.8% for Py-BPy-COF/PEG, Py-BPy^2+^-COF/PEG, and Py-BPy^+^˙-COF/PEG, respectively (Fig. 75C). Further, for laser irradiation at 1064 nm, the photothermal conversion efficiencies were 10.4, 40.1, and 55.2% for Py-BPy-COF/PEG, Py-BPy^2+^-COF/PEG, and Py-BPy^+^˙-COF/PEG, respectively (Fig. 75D). Because of the remarkable NIR absorption and photothermal conversion properties, the potential of Py-BPy^+^˙-COF/PEG as a PTA has been confirmed in antitumor experiments *in vitro* and *in vivo* using lasers at 808 and 1064 nm (Fig. 75E–I).

### 6.4 Combination therapy

Nanomaterial-based drug delivery and phototherapy have achieved certain success in preclinical tumor treatments. However, due to the inherent shortcomings of monotherapy (*e.g.*, chemotherapy resistance mechanisms, insufficient nanomaterial accumulation at the tumors, and superficial penetration depth of light) and the complex heterogeneity of tumors, it is difficult to achieve consummate therapeutic effects using monotherapy. In order to improve the treatment effects, clinically, combination therapy—also referred to as cocktail therapy—has been used as a standard method for the treatment of various cancers.^470,471^ In many cases, combining two or more therapeutic approaches not only increases the chances of a cure or long-term remission, but also reduces damage to vital organs and tissues more than that in monotherapy. In the context of nanomedicine, the realization of combination therapy heavily relies on integrating multiple treatments into a single nanoparticle. At this point, because of easy functionalization, COFs have become a fascinating nanoplatform to integrate various treatment strategies.

#### 6.4.1 Combined PDT and PTT

**Fig. 76:**
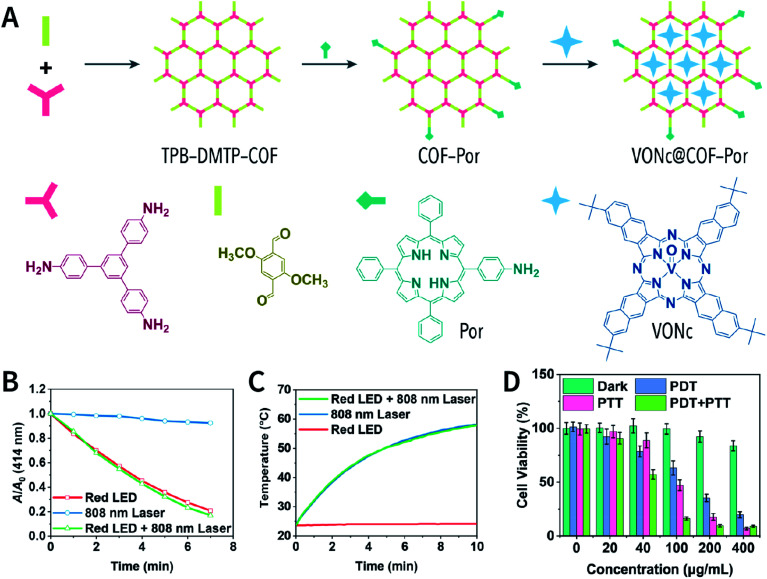
VONc@COF-Por for combinatorial PDT and PTT. (A) Synthesis of VONc@COF-Por based on BDF and host–guest encapsulation. (B) Photodynamic property of VONc@COF-Por under irradiation by a red LED. (C) Photothermal conversion induced by VONc@COF-Por under an 808 nm laser. (D) Cell viabilities of MCF-7 cells treated with monotherapy and combination therapy. Adapted with permission.^220^ Copyright 2019, American Chemical Society.

Modifying the desired PS and PTA into COFs through post-SM is one of the most feasible methods to achieve COF-based combination therapy. For example, Dong *et al.* used stepwise BDF and host–guest encapsulation to modify the PS of 5-(4-aminophenyl)-10,15,20-triphenylporphyrin (Por) and the PTA of vanadyl 2,11,20,29-tetra(*tert*-butyl)-2,3-naphthalocyanine (VONc) into TPB-DMTP-COF NCOF to obtain PDT/PTT dual-functional VONc@COF-Por nanomedicine (Fig. 76A).^220^ VONc@COF-Por maintained the nanoscale spherical morphology of TPB-DMTP-COF NCOF, where the contents of Por and VONc were 0.091 and 0.256 μmol mg^−1^, respectively. When exposed to a red LED, VONc@COF-Por effectively induced ^1^O_2_ production (Fig. 76B). When exposed to an 808 nm laser, the photothermal conversion efficiency of VONc@COF-Por was as high as 55.9% (Fig. 76C). *In vitro* antitumor experiments (Fig. 76D) showed that the IC_50_ value of the combination therapy was 42 μg mL^−1^, which was significantly lower than that of PDT (131 μg mL^−1^) or PTT (93 μg mL^−1^) monotherapy. This intensive inhibitory effect of combination therapy toward MCF-7 could be attributed to the fact that an increase in temperature enhanced PDT-induced lysosomal and mitochondrial damage, but there was no significant change in the intracellular ^1^O_2_ level when the temperature increased. *In vivo* experiments conducted in MCF-7 xenograft models revealed that despite the fact that the antitumor effect could be enhanced by increasing the light intensity and drug dose of monotherapy, it caused irreversible skin damage. However, the combination therapy effectively inhibited tumor growth and delayed tumor recurrence, while minimizing side-effects.

**Fig. 77:**
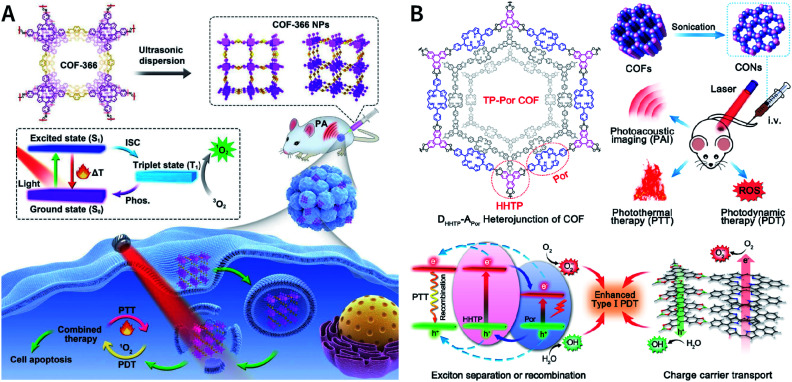
Porphyrin-based COFs for use in combinatorial PDT and PTT. (A) COF-366 for photoacoustic-imaging-guided type II PDT and PTT. Adapted with permission.^411^ Copyright 2019, Elsevier Ltd. (B) TP-Por COF for type I PDT and PTT on hypoxic tumors. Adapted with permission.^162^ Copyright 2019, American Chemical Society.

Generally, the combination of PDT and PTT required two functional components and excitation with two light sources of different wavelengths, which can lead to complicated and cumbersome applications. Recently, a multifunctional phototherapy system using single-wavelength excitation was constructed based on a porphyrin-based COF (Fig. 77A). Chen *et al.* synthesized NCOF-366 (size: 100 nm) by the ultrasonic exfoliation of bulk COF-366.^411^ The porphyrin monomer was regularly arranged in the framework to reduce PS quenching. Upon laser irradiation at 635 nm (1.5 W cm^−2^), NCOF-366 displayed efficient ^1^O_2_ generation. On the other hand, the conjugated structure in the layer broadened the absorption band of NCOF-366, making NCOF-366 (200 μg mL^−1^) yield photothermal conversion efficiency of 15.1% under laser irradiation at 635 nm (1.5 W cm^−2^). Besides, flow cytometry experiments revealed that NCOF-366 (25 μg mL^−1^) caused 70.4% of 4T1 cells undergo apoptosis under laser irradiation at 635 nm (1.5 W cm^−2^, 5 min). Photoacoustic imaging *in vivo* showed that NCOF-366 spread to the entire tumor within 1.5 h after an intratumoral injection. At this time, irradiating the tumor with a laser at 635 nm (1.5 W cm^−2^) for 5 min resulted in almost completely inhibiting tumor growth within 14 days.

Chen *et al.* also reported the donor–acceptor TP-Por COF nanosheets (Fig. 77B) for a combination of type I PDT with PTT to overcome the limited efficacy caused by hypoxia in solid tumors.^162^ Upon laser irradiation at 635 nm, the lamellar structure of TP-Por COF nanosheets was conducive to efficient charge carrier separation and transportation. Subsequently, the electrons reduced oxygen to form ˙O_2_^−^ and the holes oxidized water to generate ˙OH. Both were highly toxic ROS, leading to cell apoptosis and cell necrosis *via* type I PDT. Furthermore, the energy loss due to the inevitable nonradiative attenuation caused a rise in temperature, achieving efficient photothermal conversion for use in PTT. As expected, even under hypoxia, the combination therapy induced by TP-Por COF nanosheets still effectively inhibited the proliferation of HeLa cells. *In vivo* experiments also confirmed this excellent antitumor effect.

#### 6.4.2 Combined drug delivery and PTT

**Fig. 78:**
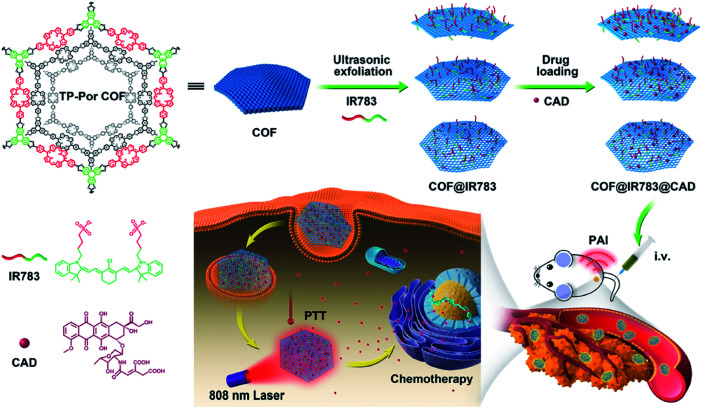
COF@IR783@CAD for chemo-photothermal therapy upon laser irradiation at 808 nm. Adapted with permission.^161^ Copyright 2019, American Chemical Society.

The TP-Por COF mentioned above was also used as a carrier for PTA and chemotherapeutic drug.^161^ COF@IR783 nanosheets (diameter: 200 nm; thickness: 9.5 nm) were prepared by adding IR783 during the ultrasonic exfoliation of TP-Por (Fig. 78). Herein, IR783 not only acted as an organic PTA for PTT, but also as a stabilizer for the nanosheets. Under laser irradiation at 808 nm, COF@IR783 did not produce ROS, but displayed photothermal conversion efficiency of 15.5%. Further loading *cis*-aconityldoxorubicin (CAD) in COF@IR783 achieved chemotherapy. As expected, COF@IR783@CAD inhibited the progress of murine breast cancer both *in vitro* and *in vivo*, as well as had photoacoustic imaging capability *in vivo*.

#### 6.4.3 Combined drug delivery and PDT

**Fig. 79:**
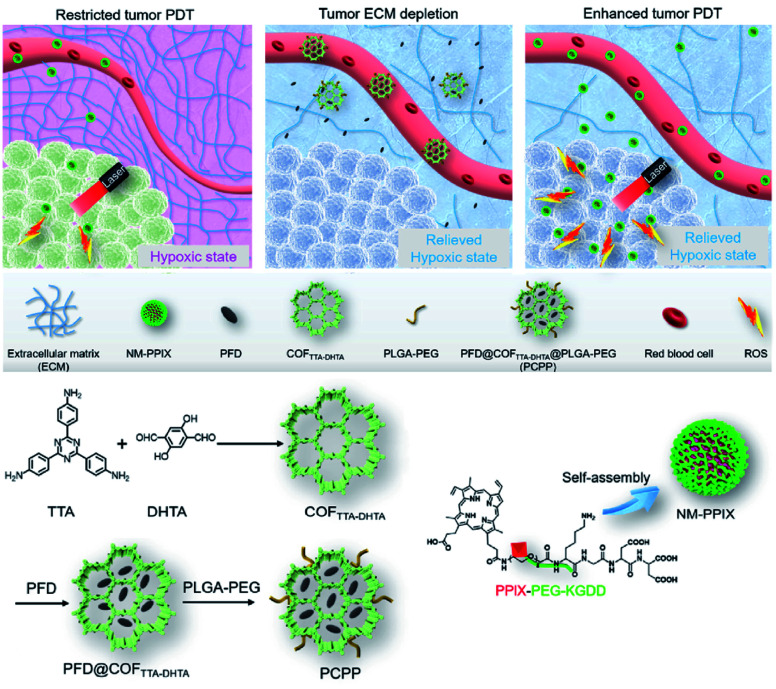
Reconstitution of the extracellular matrix to enhance tumor PDT *via* pirfenidone drug delivery based on imine-linked COF_TTA-DHTA_. Adapted with permission.^412^ Copyright 2020, Elsevier Ltd.

The COF-based combination therapy of drug delivery and PDT was also achieved by a more exquisite strategy.^412^ COF_TTA-DHTA_ with a poly(lactic-*co*-glycolic-acid)–poly(ethylene glycol) (PLGA–PEG) amphiphilic polymer coating was used to deliver the anti-fibrotic drug of pirfenidone (PFD) to the tumor site (Fig. 79). PFD@COF_TTA-DHTA_@PLGA–PEG reduced the content of collagen I and hyaluronic acid in the extracellular matrix, abated solid stress of the tumor, restored vascular function, improved tumor oxygen supply, and ultimately enhanced PDT induced by protoporphyrin-IX-conjugated peptide nanomicelles (NM-PPIX). This is a landmark study that combines tumor physiology with nanomedicine for the first time, providing a promising and readily scalable anticancer strategy for targeting the extracellular matrix.

#### 6.4.4 Photoimmunotherapy

Cancer immunotherapy is one of the most advanced clinical cancer treatment methods.^472^ It aims to use T-cell-activating cytokines, immune checkpoint inhibitors, regulatory T cell depletion, and chimeric antigen receptor to selectively inhibit tumor growth.^473,474^ Cancer immunotherapy cannot directly kill tumor cells, but it can indirectly kill tumor cells by stimulating immune cell activation, proliferation, and differentiation. A large number of clinical trials and clinical practices have confirmed the effectiveness of this method for specific populations, although the side-effects are not less than those of traditional chemotherapy to a certain extent.^475–477^ In fact, the effectiveness of immunotherapy is largely restricted by the limited activation of the immune system and nonspecific off-target activation.^478^ In theory, a therapy that selectively kills cancer cells while activating the immune response of the local host is perfect. The immunogenicity of phototherapy provides a feasible direction for achieving this goal.^479–481^ Phototherapy can efficiently activate antitumor host immunity, realizing the combination of phototherapy and immunotherapy. This combination therapy is also known as photoimmunotherapy.^482,483^

**Fig. 80:**
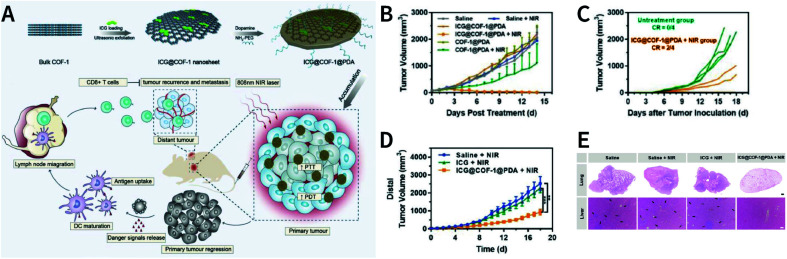
Photoimmunotherapy induced by ICG@COF-1@PDA nanosheets. (A) Preparation of ICG@COF-1@PDA and the photoimmunotherapy principle. (B) Therapeutic efficacy in CT26 colorectal tumor model. (C) Tumor growth curves of the cured mice after the second injection of tumor cells. (D) Abscopal effect in bilateral colorectal tumor model. (E) Lung and liver metastases in a triple-negative breast cancer metastasis model. Adapted with permission.^413^ Copyright 2019, Wiley-VCH Verlag GmbH & Co. KGaA, Weinheim.

Photoimmunotherapy has been proven in different types of nanomaterials.^484^ However, research on COF-based photoimmunotherapy is still in its infancy. ICG-loaded COF-1 nanosheets were surface-modified with polydopamine (PDA) and PEG to afford ICG@COF-1@PDA nanosheets with a size of 170 nm (Fig. 80A). Under laser irradiation at 808 nm, ICG@COF-1@PDA-induced PDT and PTT combination caused immunogenic cell death (ICD) of the tumor cells by triggering oxidative stress and endoplasmic reticulum stress. In the CT26 colorectal tumor model, the combination phototherapy almost completely ablated mice tumors, and there was no recurrence during the 14 day observation period (Fig. 80B). At the end of the treatment, the four cured mice were inoculated with tumor cells again. After additional observation for 18 days, 2 mice remained tumor-free (Fig. 80C), which indicated that ICG@COF-1@PDA-induced combination phototherapy activated systemic antitumor immunity. In addition, antitumor experiments conducted in the bilateral colorectal tumor model showed that this combination phototherapy induced by ICG@COF-1@PDA exhibited the abscopal effect (Fig. 80D). In terms of mechanism, this combination phototherapy upregulated the damage-associated molecular patterns (DAMPs) including HSP70 and HMGB1, promoted dendritic cell maturation, subsequently induced CD8^+^ T cells to infiltrate into distant tumors, upregulated IFN-γ in the distal tumor, and finally slowed the growth of untreated distal tumors. More importantly, in a triple-negative breast cancer metastasis model, ICG@COF-1@PDA-induced combination phototherapy even suppressed lung metastasis and liver metastasis (Fig. 80E).

Combining phototherapy with immune checkpoint inhibitors can further enhance the activation of the immune system, enhancing antitumor therapy. In 2020, Pang *et al.* reported the combination of PDT, PTT, and α-PD-L1 checkpoint blockade therapy.^414^ Using π–π interactions, ICG was adsorbed in TAPB-BTCA-COF, and then chicken ovalbumin (OVA) was coated on the surface of COF@ICG *via* electrostatic interactions. The resulting COF@ICG@OVA had a photothermal conversion efficiency of 35.8% and the ability to generate ROS under laser irradiation at 650 and 808 nm. The combination of PDT and PTT induced tumor-associated antigen production. When further combined with α-PD-L1 therapy, the three-in-one combination therapy not only inhibited the growth of the primary tumor, but also delayed distant tumor growth and cancer lung metastasis.

#### 6.4.5 Combined PDT and ion-interference therapy

Some metal and nonmetal ions (*e.g.*, Na^+^, K^+^, Ca^2+^, Zn^2+^, Mg^2+^, Fe^2+^, Cl^−^, H_2_PO_4_^−^, and HCO_3_^−^) participate in many important processes in cell biology, such as maintaining the osmotic pressure and acid–base balance, activating signal pathways, getting involved in cellular communication, and constituting enzymes.^485^ Their abnormal distribution and accumulation can interfere with these processes and induce irreversible cell damage.^486,487^ In this context, inorganic ions can be effectively exploited against cancers, which is called ion-interference therapy.^488^ However, due to their limitation of short circulation time and regulation of exogenous ions by cells, inorganic ions are relatively difficult to be directly used in antitumor therapy. Therefore, a combination therapy has come into legitimacy.^489–493^

**Fig. 81:**
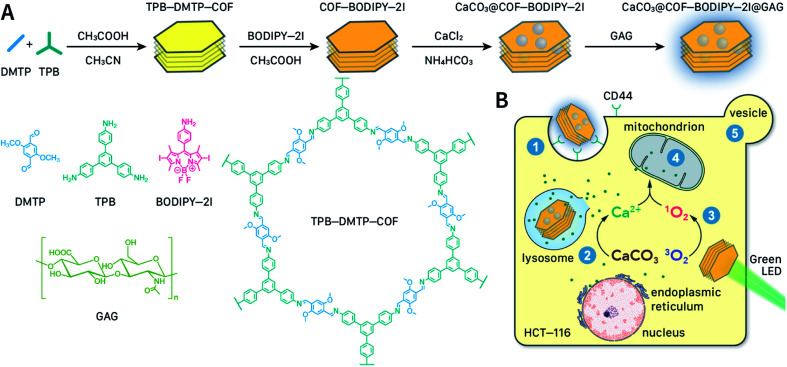
COF-based composite nanomaterial for synergistic Ca^2+^ overload and PDT. (A) Synthesis of CaCO_3_@COF-BODIPY-2I@GAG. (B) Schematic illustration of combination therapy under green LED irradiation. (1) CD44-mediated cellular uptake; (2) CaCO_3_ decomposition in lysosomes; (3) ^1^O_2_ generation induced by BODIPY-2I PS under green LED; (4) damaged mitochondrial function co-induced by ^1^O_2_ and Ca^2+^; (5) tumor cell blebbing due to oncosis. Adapted with permission.^415^ Copyright 2020, Wiley-VCH Verlag GmbH & Co. KGaA, Weinheim.

Very recently, Dong *et al.* reported a TPB-DMTP-COF-based nanoagent, namely, CaCO_3_@COF-BODIPY-2I@GAG,^415^ which comprised BODIPY-2I PS, CaCO_3_ nanoparticle, and glycosaminoglycan (GAG) CD44-target coating (Fig. 81). Under green LED irradiation, the surface-decorated BODIPY-2I could not only generate ^1^O_2_ to directly kill the tumor cells, but also destroy the ability of mitochondria to regulate Ca^2+^. Under these precarious circumstances, Ca^2+^ released into the cytoplasm due to CaCO_3_ decomposition in the lysosomes irreversibly caused intracellular Ca^2+^ overload. As a result, enhanced antitumor efficiency could be achieved *via* the synergistic action of PDT and Ca^2+^ overload. On the other hand, as a specific targeting agent for CD44 receptors on tumor cells in the digestive tract, the GAG coating significantly promoted nanoagent uptake in the HCT-116 cells, consequently achieving more effective antitumor activity against colorectal carcinoma along with a weaker side-effect on normal tissues. Hopefully, the COF-based ion-interference therapy discussed here can be incorporated with other treatment methods to realize efficient synergistic cancer therapy.

## 7 Summary and outlook

In this review, we have provided a detailed summary of the recent advances made in COFs as multifunctional therapeutic platforms in oncology, including the preparation of COFs, reduction in the size of COFs to the nanoscale, introduction of the desired functional groups into COFs, and existing COF-based therapeutics. COFs exhibit several significant advantages in the biomedical fields, such as high bioaffinity and biocompatibility, ordered framework structures, adjustable and open pore structures, and easily modifiable surface and pore walls. These distinct properties are highly advantageous for realizing biomedical applications *in vivo*. Although COF-based therapeutic systems are still in their infancy, their fascinating properties and promising potential for oncology have inspired an increased number of researchers to dedicate their efforts to this promising field.

However, the current challenges and limitations faced by COFs cannot be ignored, which may become obstacles to clinical translation.

(i) Preparing NCOFs with high crystallinity and appropriate size is a huge challenge, which even becomes a major obstacle hindering the fundamental laboratory research of COFs. Despite the great efforts that have been made to develop various nanocrystallization methods, the difficulty of large-scale production and poor batch-to-batch consistency are still the bottlenecks restricting NCOF preparation. Unsatisfactory NCOF materials have questioned the uniformity and homogeneity of drug carriers, as well as the reliability of drug release kinetics studies. This is regarded as one of the most challenging issues facing the entire field of nanomedicine.^494^

(ii) The stability of COFs is a double-edged sword. Since most linkages are reversible chemical bonds, COFs can be broken down into organic small molecules or polymer fragments in the body, which leads to a limited shelf life but reduces the physiological toxicity of COFs. On the other hand, COFs involving irreversible chemical bonds may be difficult to decompose *in vivo*. The enrichment of these exogenous COF particles in the body may cause serious health risks. Therefore, it is necessary to thoroughly evaluate the decomposition of COFs *in vitro* and *in vivo* to balance their stability and shelf life: completing the intended function and degradation at the right time in the right way is the most ideal state.

(iii) The biological safety of COFs has not been studied in detail. Until now, there are only some preliminary *in vitro* results mentioned in the literature, mainly focusing on the cytotoxicity of COFs. Due to their relatively short development history, a thorough assessment of their hemocompatibility, histocompatibility, cytotoxicity, neurotoxicity, and genotoxicity at the cellular and tissue levels are necessary. Furthermore, evaluating their acute toxicity, carcinogenicity, reproductive toxicity, and immunogenicity at the animal level is also a problem that needs to be resolved in the future.^495^ Considering that most of the degradation products of COFs are aromatic compounds, it is still unknown whether they are toxic or not.

(iv) The application of COFs in tumor imaging is limited by the composition of light elements. Magnetic resonance imaging (MRI), ultrasound imaging (USI), computed tomography (CT), and positron emission tomography (PET), which are widely used clinically, rely on high-atomic-number metal or nonmetal contrast media (such as Fe, Ga, Mn, and I).^496^ Unfortunately, as compared to other nanomaterials such as metal oxides and MOFs,^497^ COFs themselves do not contain metals, resulting in their inapplicability in such imaging techniques. To our delight, COFs can be metalized through pre-SM or post-SM, which opens an avenue for imaging applications. In addition, studies on COFs for optical imaging may be a growing research area of COF-based tumor imaging, such as two-photon fluorescence imaging.^498^

(v) Due to the complexity and diversity of cancers, it is necessary to develop an intelligent and versatile integrated system for diagnosis and targeting treatment (theranostics platform) to achieve accurate cancer treatment based on COFs. Although currently reported active targeting groups can partially improve the uptake of COFs by tumor cells, it is urgently needed to study exclusive targeting materials that only identify tumor cells to achieve effective tumor inhibition without any side-effects on healthy cells. Fortunately, the diversity of the functional groups in COFs ensures that multiple functions of COFs can be readily achieved through post-SM.

(vi) Difficulties in the preparation of COF single crystals may lead to unreasonable structural analyses. Structural analysis based on PXRD follows the hypothesis–validation pattern. However, due to the broadening of the diffraction peaks, COFs with different structures may have similar PXRD patterns.^75,499^ This is a serious challenge for the resolution of COFs with complex structures. In addition, the stacking of COF layers may not be simple overlapping or staggered stacking. Some COFs with interlayer slips have been reported,^500–507^ which further increases the difficulty of structural analysis.

Overall, COFs are a new member of the crystalline porous material family. In recent years, studies on COFs have mainly focused on the development of new structures and new synthesis strategies, and the applications of COFs in a large part has concentrated on heterogeneous catalysis and separation, while the study of COFs in the cancer biomedical field is still in its infancy. We anticipate that COFs can become a new growth point for cancer treatment and promote the development of nanomedicine, particularly clinical medicine.

## Conflicts of interest

There are no conflicts to declare.

## Supplementary Material
